# Sharp structural variability of the Gorda slab imaged by a fiber array

**DOI:** 10.1038/s41467-026-76002-8

**Published:** 2026-07-25

**Authors:** James Atterholt, Jeffrey J. McGuire, Andrew J. Barbour, Morgan P. Moschetti

**Affiliations:** 1 Geologic Hazards Science Center, U.S. Geological Survey, Golden, CO USA; 2 Department of Geosciences, University of Arizona, Tucson, AZ USA; 3 Earthquake Science Center, U.S. Geological Survey, Moffett Field, CA USA

**Keywords:** Seismology, Geophysics, Tectonics

## Abstract

Structural variability at the subduction interface imposes stress and strength heterogeneity that affects the behavior of megathrust earthquakes. Characterizing this variability can be challenging because the slab interface is often deep and seismically quiet. Seismic wavefields from intraslab earthquakes can contain high-frequency phase conversions from the megathrust fault that are delayed and distorted by interface topography and velocity heterogeneity. Distributed acoustic sensing (DAS) allows for long-term deployments of dense seismic arrays that can yield many observations of these converted phases at high spatial resolution over large areas. Here, we develop a technique to identify and enhance slab-converted phases in DAS data, and we pick the differential arrival times and relative polarities between the direct and converted phases at short wavelength along the fiber optic cable. We apply this framework to earthquake wavefields measured by a DAS array at the southern end of the Cascadia Subduction Zone, above the locked zone, which suggests kilometer-scale interface roughness and variations in crustal thickness and absolute depth of the Gorda slab. Converted phase wavefields also suggest sharp variability in the presence and position of fluids in the slab and forearc crust.

## Abstract (web summary)

The structure of megathrust faults shapes the behavior of the world’s largest earthquakes. This study introduces a way to image these faults in detail using local earthquakes measured by telecom fibers transformed into sensing networks.

## Introduction

Imaging megathrust fault structure is a fundamental component of understanding Earth’s largest earthquakes. Megathrust roughness expectedly plays an important role in rupture phenomenology, but there is disagreement regarding the nature of this control^1^. Some studies suggest roughness promotes concentrations of normal stress that generate asperity-like slip behavior^2^. Other work suggests rough patches of megathrust faults act as seismic barriers that inhibit the growth of large earthquakes^3^. Beyond roughness, complexity at the interface imposed by subducting sediments and shear further complicate the strength of the interface^4–6^. Characterizing fine-scale structure of the subduction interface is necessary to address this problem, because it allows for the direct comparison of observed slip behavior to megathrust topography and rheology. By extension, these fault characteristics could in part explain the enigmatic along-strike variability in interseismic locking^7^. Characterizing the subduction interface more precisely may thus allow for better forecasting of megathrust earthquake phenomenology.

Several approaches have been taken to characterize subduction zone geometry, each with their own set of strengths and limitations. Controlled-source seismic profiles can yield high-resolution megathrust geometry^5,8^, but these profiles typically lose resolution at depth and are often impractical onshore. Teleseismic receiver functions access megathrust faults at greater depths^4,9,10^, but these are low-frequency measurements that have limited vertical resolution. Earthquake hypocenters and focal mechanisms define fault planes^6^, but in many regions small earthquakes do not rupture the megathrust, instead favoring faults within the slab or the forearc crust^11,12^.

Intraslab earthquakes generate wavefields that produce phase conversions from the slab interface^13^. These converted phases have high-frequency contents and can yield sharp images of the megathrust fault. But there are two significant challenges with using them to map slab geometry. 1. These phases are difficult to identify and classify. 2. Hypocenter depth uncertainty makes it hard to translate these observations to fine-scale interface geometry. Both of these challenges can be ameliorated using dense seismic arrays. Dense arrays allow for the use of array-side moveout and coherence for secondary phase identification, and they allow for structural inference using multiple phase observations from a single event.

Distributed acoustic sensing (DAS) is an emergent fiber optic sensing technique that uses phase interferometry of backscattered light to transform fiber-optic cables into dense arrays of strainmeters^14^. This technique is powerful because it enables meter-scale coverage over tens to hundreds of kilometers, allows for the long-term deployment of dense seismic arrays, and leverages existing telecommunications infrastructure, which is becoming increasingly widespread both onshore and offshore. Recently, DAS was successfully used to image the Mohorovičić (Moho) discontinuity by autocorrelating earthquake wavefields to extract the Moho reflected phase, and array-side coherence and moveout was used for identification and classification^15^. We modify this approach to make it applicable to secondary phases generated by subducting slabs.

In this work, we make the following contributions. 1. We develop an approach that leverages fiber-optic arrays to extract secondary phase arrivals, even those below the noise floor. 2. We apply this approach to identify and pick converted phases from the Gorda slab using a fiber-optic array between Arcata and Eureka, California. 3. We use these observations, combined with three-dimensional (3D) raytracing, to infer absolute slab depth and crustal thickness, fluid variability, and sub-kilometer megathrust geometry. This study has important implications for how a new generation of slab models may be built with fine-scale interface structure using in situ fiber networks.

## Results

### Measuring and classifying slab-converted phases in fiber data

**Fig. 1:**
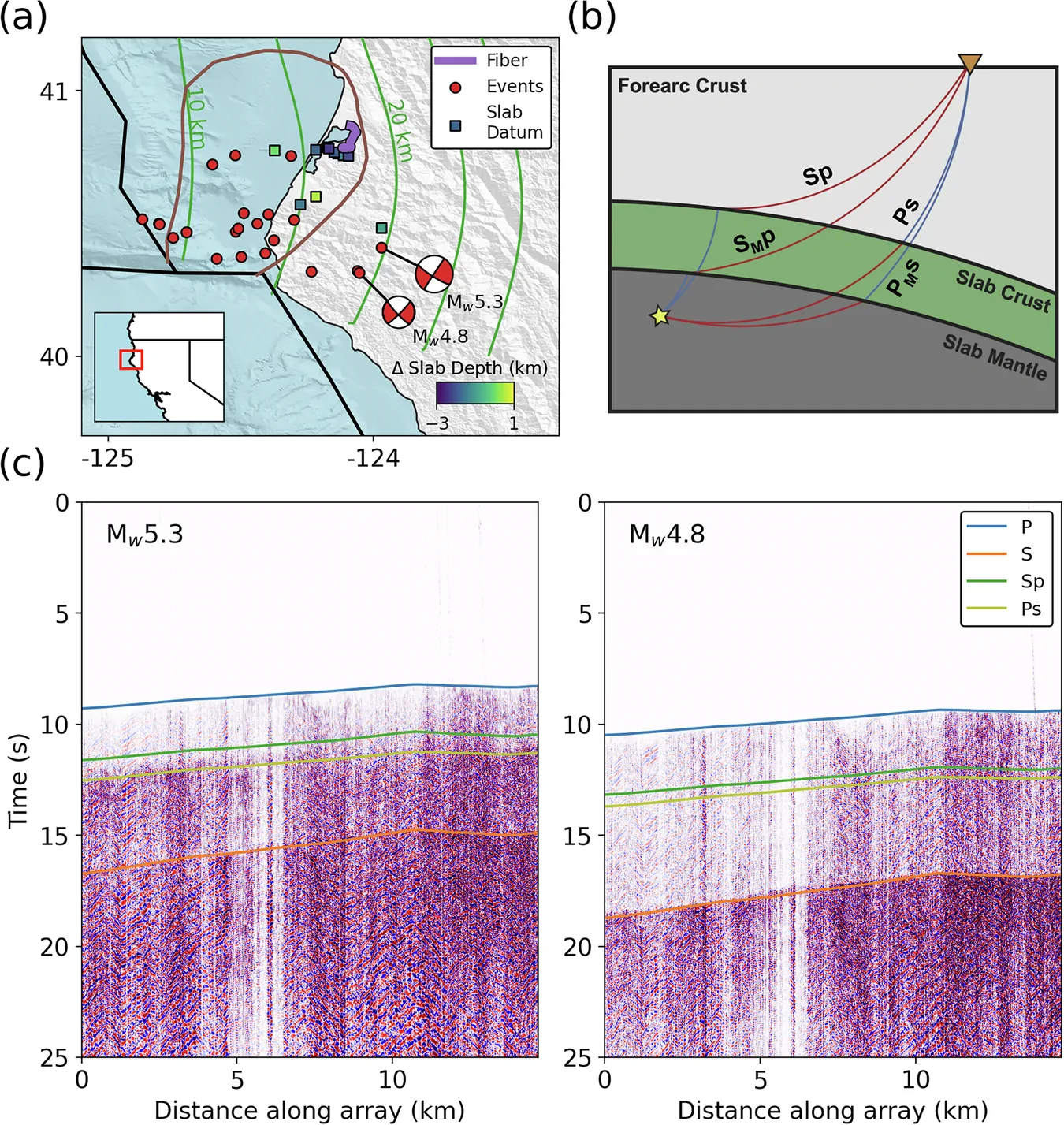
Summary of observations. **a** Map of intraslab earthquakes used in this study. Green contours represent the interface depths of Slab2^29^. Brown contour encompasses zone in which the slip deficit exceeds 20 mm/yr^18^. Black lines are plate boundaries defined in Bird et al.^71^. Also plotted as square points are changes to Slab2 resolved in this study (Fig. 3b) at the average location of the conversion points (which is distinct from the event epicenter as illustrated in 1b) used to make the estimate (see Methods). **b** Schematic showing the phases of interest in this study and their approximate relative conversion points. The star indicates the source, and the triangle indicates the receiver. **c** Earthquake wavefields for ${M}_{w}5.3$ and ${M}_{w}4.8$ earthquakes highlighted in (**a**). Expected arrival times are computed using 3D raytracing assuming conversion points at the Slab2 interface. Time is relative to the event origin time.

In May of 2022, a DAS array was deployed along ~15 km of telecommunications fiber north of Cape Mendocino and above the Gorda slab (Fig. 1)^16,17^. The array traverses an azimuthally diverse path between Arcata and Eureka, California. This array recorded many earthquakes, including a large number of aftershocks from the 2022 moment magnitude (M_w_) 6.4 Ferndale earthquake and the 2024 M_w_7.0 Cape Mendocino earthquake. In this dataset^16,17^, we identified 23 M2.5+ intraslab events (Fig. 1). Most of the events used in this study are within what we deem the locked zone, which we define as portions of the megathrust with slip deficits greater than 20 mm/yr (Fig. 1), which is ~50% of the Gorda-Pacific convergence rate^18^. Some of these events, such as the M_w_5.3 earthquake shown in Fig. 1, generate wavefields that have visually clear, secondary onsets of energy that coincide with the expected arrival times of slab-converted phases computed with 3D raytracing (see Methods). In other cases, intraslab earthquake wavefields show little evidence of phase conversions. For example, we do not observe converted phases in the M_w_4.8 earthquake shown in Fig. 1, which is near the M_w_5.3 event with a similar source mechanism and similar depth.

The examples cited above illustrate several challenges with using DAS data to measure converted phases that we attempt to address in this study. Firstly, converted phases off slab interfaces have historically been difficult to identify across many events, even in array data^13,19^. As shown in Fig. 1 and observed elsewhere, the observability and amplitude of converted phases from similarly located events recorded by the same stations often show substantial variation. The nature of the conversion (S-to-P versus P-to-S; Fig. 1) is also difficult to discern, especially when these converted phases are expected to arrive at similar times. From here on, we refer to P-to-S and S-to-P conversions occurring near the top of the slab as Ps and Sp, respectively. The absence of polarization information in DAS amplifies this problem. Weak observations of these phases are difficult to pick objectively. Picking can be even more difficult in DAS data, because phase arrivals are often dominated by surface waves scattered from the direct phase due to shallow structure, rather than the direct arrival. We use an approach that builds on the method described in Atterholt and Zhan^15^ that alleviates these problems by providing a signal processing framework to amplify weak signals and make precise picks of converted phase arrivals.

**Fig. 2:**
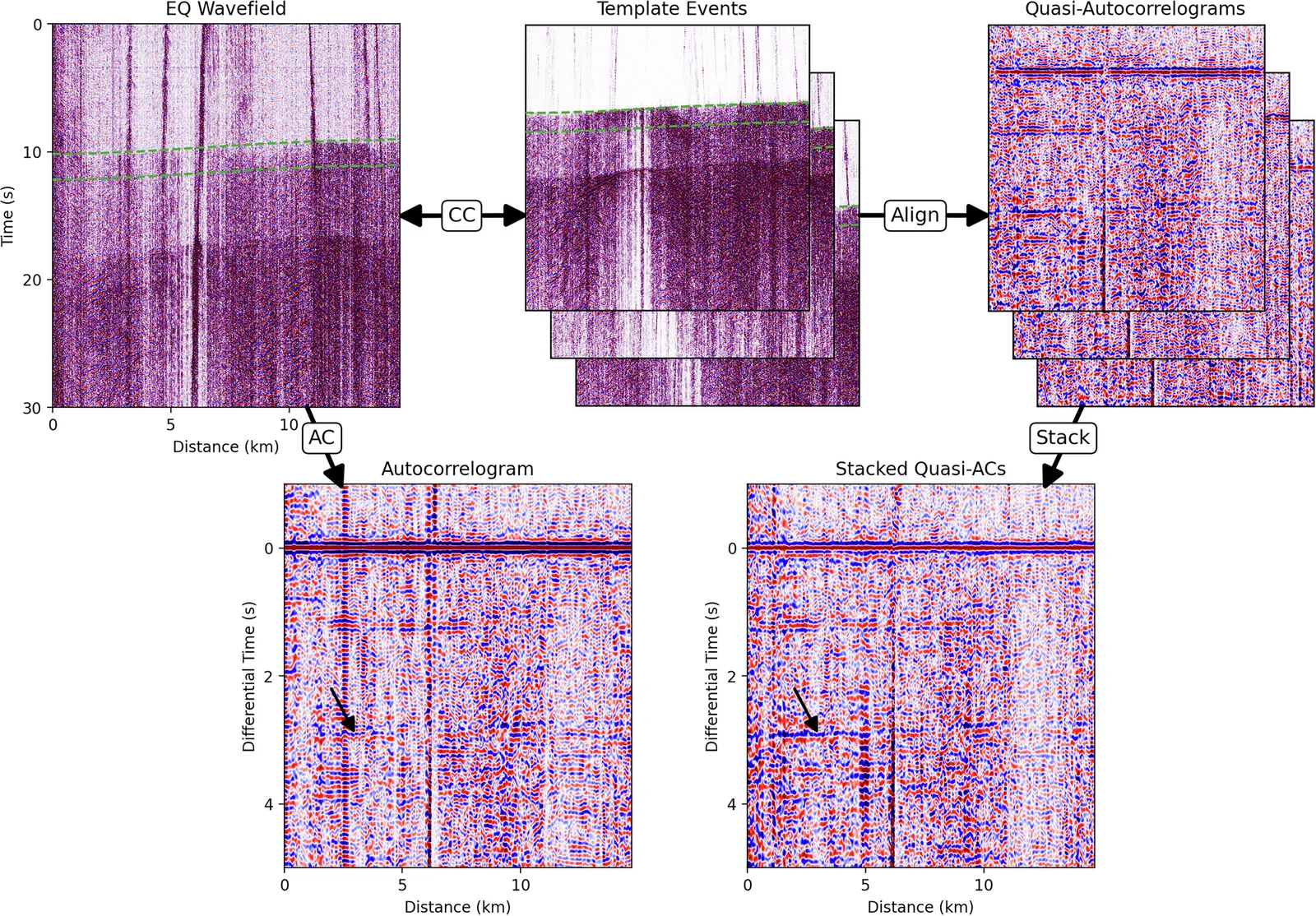
Quasi-autocorrelogram workflow used in this study. Workflow shows the utility of cross-correlating (CC) a noisy earthquake wavefield (nc73947920; top left) with scattered wavefields from template events (top middle) to generate quasi-autocorrelograms (top right). Stacking the quasi-autocorrelograms (bottom right) produces a higher quality converted phase signal than direct autocorrelation (AC) of the earthquake wavefield with its scattered wavefield from the P-wave (bottom left). Note that stacking does not reduce precision, and phases are still resolved above 5 Hz. Green dotted lines indicate the 2 s window used to capture the scattered wavefield from the P-wave. Black arrow in the bottom two plots indicate the converted phase observation.

The framework described in Atterholt and Zhan^15^ uses the observation that scattered surfaces waves that dominate DAS wavefields are generated by array-side, near-surface structure^20^. Thus, the first arriving P wave and secondary phases generate similar scattered wavefields. Correlating the first few seconds of an earthquake wavefield following the P wave with the full wavefield generates peaks at the differential time between the first P arrival and secondary arrivals. The correlogram wavefield is much simpler, and these differential time peaks can be picked precisely using a local maximum (or minimum). We found that for slab phase conversions, the correlogram peaks were generally very weak. In order to amplify weak converted phase signals, we further develop this approach with the understanding that the scattered wavefields, which are generated locally, are expectedly similar between earthquakes as well. We can thus use the first arrivals of many earthquakes, which need not be intraslab, as template scattered wavefields to correlate with intraslab earthquake wavefields. The effectiveness of the template is related to the spatial proximity of the template earthquake to the target event (Supplementary Fig. S1). These correlograms can be aligned by the correlogram peak between first arrivals and subsequently stacked to produce correlogram wavefields with secondary phase peaks with enhanced signal-to-noise ratios (see Methods for details). This workflow is summarized in Fig. 2, which shows how a weak secondary arrival in a noisy earthquake can be retrieved by this methodology.

**Fig. 3:**
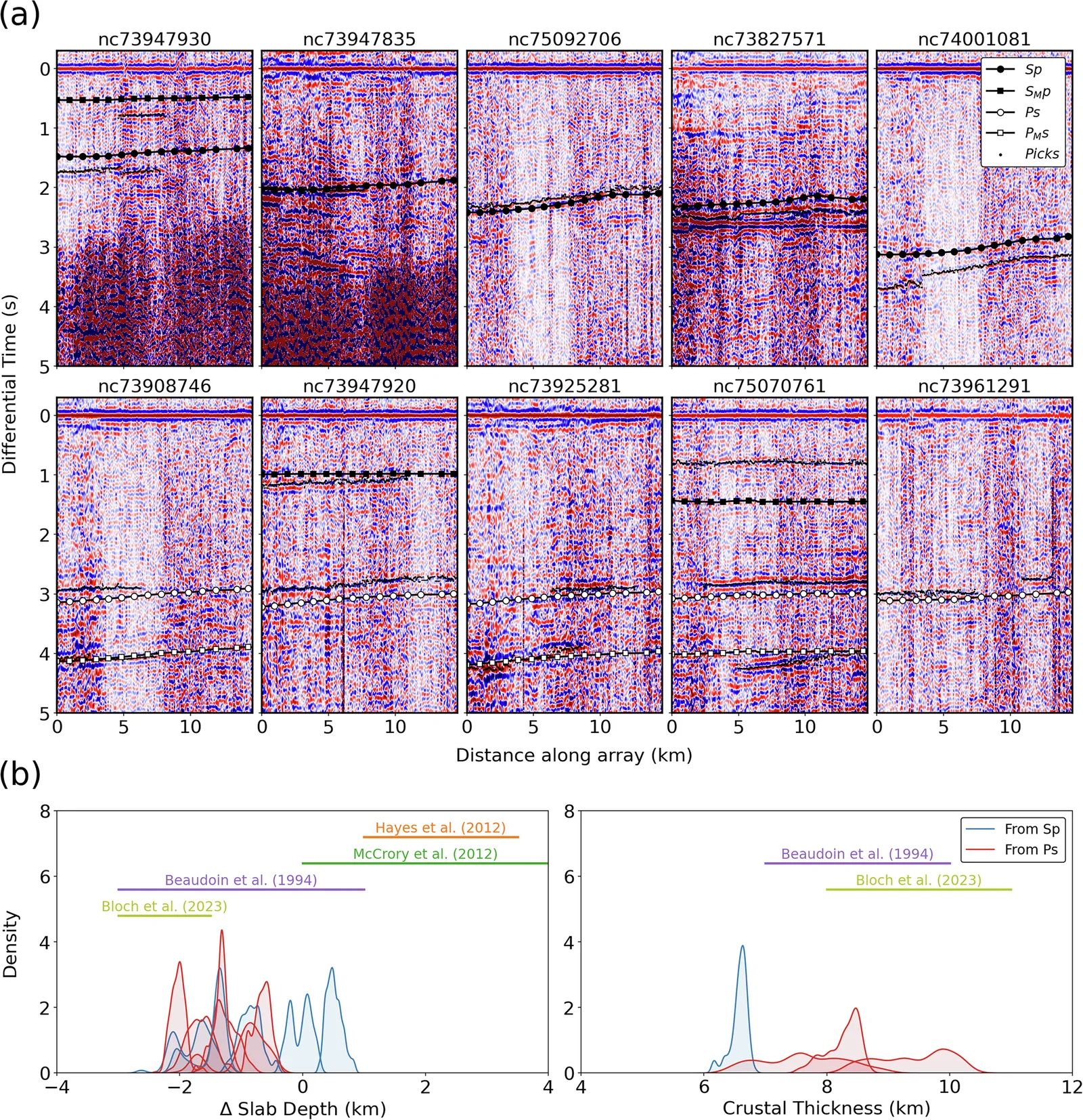
Summary of converted phase observations and inversion results. **a** Stacked quasi-autocorrelograms from 10 of the 11 events with observable phase conversions in their P-wave coda. Black scatter points indicate picks of Ps-type and Sp-type conversions. Expected arrival times from 3D raytracing and the reference model are also shown as lines with markers in the legend. Picked phases are ultimately classified by comparison with the expected phase arrival times. **b** Inversion results (see Methods) for necessary changes (△) in absolute slab depth from the Slab2 model^29^ and, for earthquakes where Moho and interface conversions of the same type were observed, inversion results for crustal thickness. Each distribution is shown as a normalized density and includes inversion results from all observations from an event. Included are estimates of the approximate range of possible values from other slab models in the study region^4,12,26,72^.

We apply this technique to all 23 intraslab earthquakes identified for this study. We find evidence of phase conversions in 11 of the events (Supplementary Table S1). While this number is generally not reported in other converted phase studies^13,19^, this is a high proportion of observations compared to what could be discerned visually with thousands of nearby channels (3 of 23). The precision yielded by our stacked correlogram-based measurement makes the distinction between P-to-S and S-to-P phases clear by comparison with the differential time and the array-side moveout to those expected from the 3D raytracing (see Methods). These observations are summarized in Fig. 3. In several cases, we also observe conversions from the slab Moho, which can be classified by comparing these phase arrivals to expected arrival times computed using an interface that is 7 km deeper than the slab interface. From here on, we refer to P-to-S and S-to-P conversions near the slab Moho as P_M_s and S_M_p, respectively. Once classified, we make picks on the phase differential times by finding a local maximum or minimum within a time window of the expected arrival time of the phase of interest. Whether the maximum or minimum is used is decided by visual inspection for an axis of symmetry in the stacked autocorrelogram peak (or trough).

### Interpreting variations in the shape and amplitude of converted phases

**Fig. 4:**
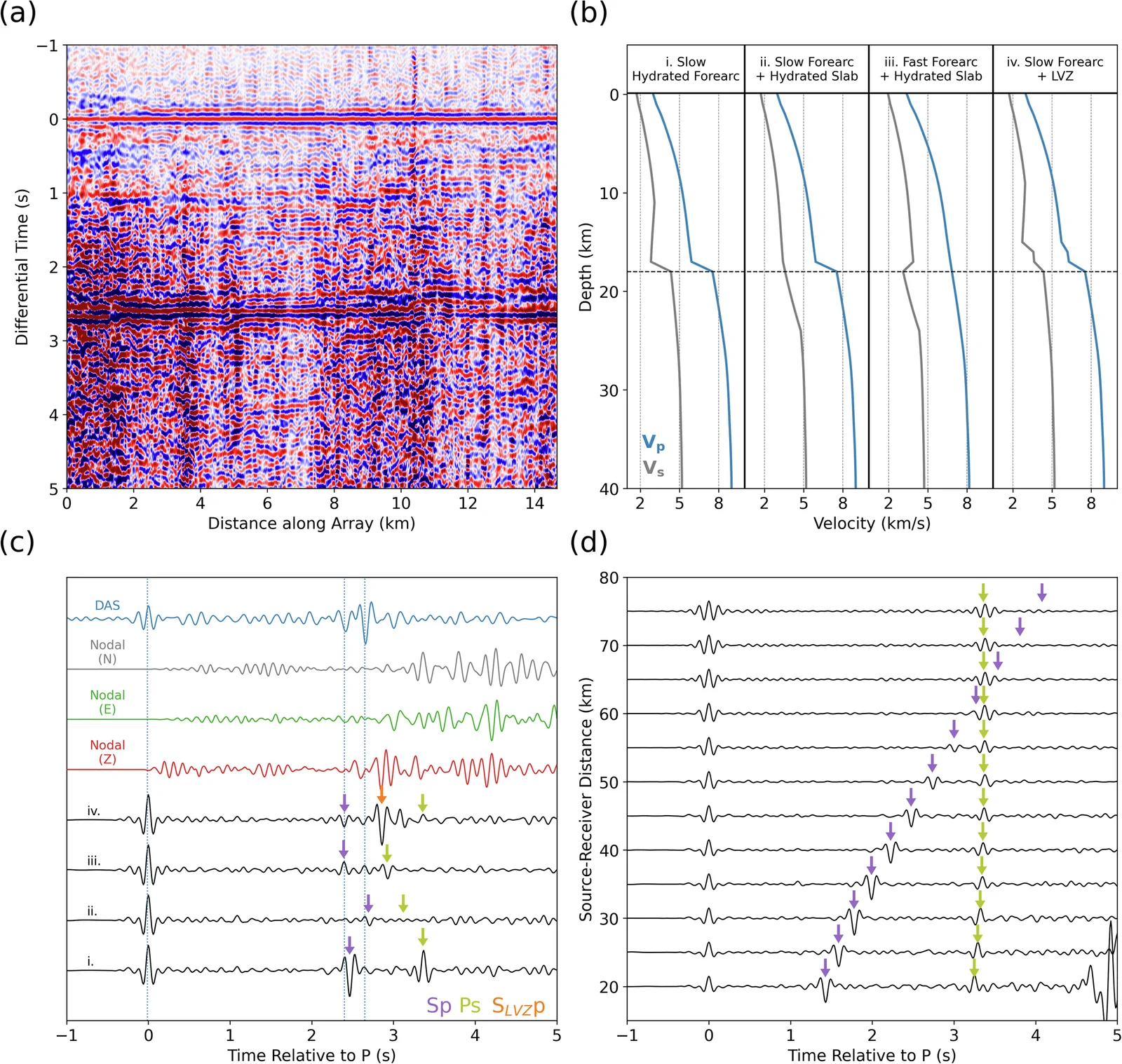
Matching converted phase observations with synthetics. **a** Stacked quasi-autocorrelogram of ${M}_{w}5.3$ earthquake (nc73827571) wavefield shown in Fig. 1c. **b** 1D velocity models used to generate the synthetic waveforms shown in (**c**); these imply different characteristics of the crust and slab, as labeled above each model. Slab Moho is not included for simplicity; horizontal dashed line corresponds to top of slab crust. Vp/Vs ratios are plotted explicitly in Supplementary Fig. S11. **c** Observed and synthetic waveforms. Labels i-iv correspond to north-south synthetic autocorrelograms from strain waveforms generated with models in (**b**) using a source with the same mechanism, distance (45 km), and depth (30.6 km) as the ${M}_{w}5.3$ event. Distributed acoustic sensing (DAS) waveform is the average of the autocorrelogram from 12 to 13 km along-fiber distance. Nodal data show north (N), east (E), and vertical (Z) particle velocities from a seismometer located at 12.5 km along the array. The colored arrows correspond to the computed onset time of phases in the legend. Sp and Ps refer to S-to-P and P-to-S conversions off the interface in (**b**), respectively. S_LVZ_p refers to the phase conversion off the top of the weak zone in model (iv) shown in (**b**). Vertical dotted lines correspond to troughs in the DAS correlogram. **d** Synthetic autocorrelograms from strain waveforms computed using model i for a range of source receiver distances using the mechanism and depth of the ${M}_{w}5.3$ event. Colored arrows correspond the onset time of the same phases used in (**c**).

As demonstrated in Fig. 1, these intraslab earthquake wavefields show strong variability in the amplitude and observability of slab-converted phases. Recognizing that interpretations of this variability are subject to nonuniqueness, we discuss some potential models that could explain this variability. Using the M_w_5.3 intraslab earthquake shown in Fig. 1 as an example, we evaluate how different structural models can be applied to reproduce the range of observations we make in this study. The stacked autocorrelogram of this event is shown in Fig. 4. This particular earthquake is exceptional in that its conversion is very high amplitude and shows two overlapping correlogram peaks with negative polarities relative to the first arrival. This is also the only earthquake for which we have collocated nodal seismometers, and we use those data to improve the confidence in our phase classification.

When trying to model a possible control on conversion efficiency, we take an inference mode approach^21^. That is, we constrain the range of possible explanatory models using prior knowledge about the region. We know that there is evidence for strong variability in fluid content in the vicinity of the Gorda slab^22–24^ and that the forearc crust is likely slower than the subducting slab^23^. We thus model variability in converted phase observations in the context of fluid variability and assume a forearc crustal velocity that is slower than or equal to the slab velocity. Fluids can be incorporated into a velocity model by increasing the Vp/Vs ratio, which we accomplish by reducing Vs. We consider four one-dimensional (1D) models (modified from an average of Guo et al.^22^) that mimic or vary results from prior studies and produce a range of observations (see Methods; Fig. 4). i. A model with slow, hydrated forearc crust^22,23^. ii. A model with slow forearc crust and hydrated slab crust^22^. iii. A model with fast forearc crust^23^ (i.e., a smoother transition from slab to forearc) and hydrated slab crust. iv. A model with slow, hydrated forearc crust and a low velocity upper slab crust^4^.

We can first confirm that the observed converted phases in this earthquake are Sp (Fig. 1), both because of the agreement with the expected differential time and because this conversion produces no observable energy in the horizontal nodal data (Fig. 4). Additionally, the polarity information helps distinguish between models. Only the models with slow, hydrated forearc crust (e.g., model i) produce an Sp phase with the opposite polarity as the first arrival, indicating these models best fit the M_w_5.3 data. We observe, however, that a slow forearc crust with a hydrated slab substantially reduces the amplitude of the converted phases. Hence, by changing the location of fluids within the subduction zone (e.g., model ii), we can reproduce the observation of the absence of converted phases in the M_w_4.8 earthquake shown in Fig. 1. A faster forearc crust with a hydrated slab (e.g., model iii) is capable of producing an Sp phase with the same polarity as the first arrival and a Ps phase with the opposite polarity. This is the case for most of our observations, which are all west of the M_w_5.3 Sp conversion point. Finally, the M_w_5.3 produces two vertically polarized peaks with the same polarity and variable differential time. One way to fit these peaks is with a narrow (<1 km) zone at the fault interface with seismic velocities that are higher than those of the forearc crust (e.g., model iv). This is within the range of expected thicknesses for subducted marine sediments^25,26^ or finiteness of the megathrust fault due to shear^4,6^, but these layers are generally expected to have lower velocities than the overlying forearc crust^4,27^. Additional work can help determine the nature of this boundary; however, this suggests that the high frequency content of our measurements allow for imaging narrow layers at depth and opens new avenues for testing of causal mechanisms for down-dip and along-strike variations in fault locking^18^.

To add further complexity to this analysis, we also notice that the source-receiver distance places a strong control on the amplitude of the Sp conversion (Fig. 4). In particular, for the M_w_5.3 hypocenter and slab depth, assuming a slow, hydrated forearc crust model (model i), the Sp phase is the highest amplitude phase for the first 50 km. Near this source-receiver distance we observe the critical angle of reflection and the conversion disappears. We expect, then, that for earthquakes more than 50 km away from the array, we would be much more likely to observe Ps rather than Sp. This matches our observations well and makes comparing the amplitudes of Sp between events challenging. This provides an additional phase classification metric for when the expected arrival times of Sp and Ps are coincident. This also offers an alternative explanation for the lack of observable Sp phase in the M_w_4.8 earthquake. The M_w_4.8 event is farther away from our array than the M_w_5.3 event (~46 km versus ~55 km). The distance range between 46 and 55 km encompasses a the sharp decay in amplitude of Sp observed in our synthetics shown in Fig. 4d.

The phase conversion observed in the M_w_5.3 earthquake is exceptional compared to the other converted phase observations (Fig. 3). All other observations of Sp have positive relative polarities, and all observations of Ps have negative relative polarities. This would suggest that the other observations are more consistent with phase conversions generated by a model of a fast forearc and hydrated slab (model iii; Fig. 4). The explanation for this difference may be that the M_w_5.3 earthquake is located to the east of all other intraslab earthquakes used and outside the high slip deficit patch that encompasses most of the events and phase conversion locations in this study^18^ (Fig. 1). Previous studies have observed a correlation between slab hydration and interface locking^22^, expectedly because the slab is dehydrated as it transitions into a ductile regime. The results of this study support this observation, and relatedly support the conclusion that, within the locked zone, the pore fluid pressure in the slab crust is elevated. This may lead to a lower effective stress on the megathrust^28^. With a more extensive array aperture, it may be possible to observe a change in the polarity of phase conversions, consistent with a transition from model iii to model i, within a single event wavefield. This would provide a short-wavelength constraint on the downdip position of slab dehydration.

### Inverting for megathrust structure and evaluating uncertainty

To invert for a modified slab geometry, we use expected arrivals from 3D raytracing for a range of slab depths to define a linear interpolation function that maps slab interface depth and slab Moho depth to phase differential time. We can then search this interpolant for the slab parameter depths that best fit our observations (see Methods). Owing to the variable quality of the autocorrelograms, we use two approaches to maximize the information gained from the full set of observations. For all autocorrelograms, we combine all the inverted slab interface and Moho depths into a distribution of solutions that together can fit all the data. This approach prioritizes the high temporal frequency phases that were confidently identified and classified using the spatial density of DAS to infer slab properties with high precision (~half the period of the correlation peak) and a measure of uncertainty. For the highest quality autocorrelograms, we can interpret slab structure at high spatial frequency with observations from a single event. This provides a window into sub-kilometer slab structure at a depth and location that is inaccessible by offshore active source experiments.

We use the first of these approaches to infer the absolute slab depth relative to the Slab2 model for Cascadia^29^ in the form of a set of distributions of slab depth measurements, one for each converted phase instance. The absolute slab depth distributions, relative to Slab2, are shown in Figs. 1 and 3b. These show that there is some variability in how well the Slab2 model captures our data, ranging from being 1 km too shallow to 2.5 km too deep. We can compare these results to previous studies by subtracting Slab2 from those models in a narrow spatial window encompassing the measurements made in this study. We find that our results are consistent with other measures of slab depth in the region^4,26^. The majority of the observations suggest a shallower slab geometry. The modified slab depths as measured by the Sp conversions strongly trade off with event depth (Supplementary Fig. S7; see Methods), which have higher uncertainties for events offshore. However, those measured with Ps conversions are far less sensitive to source depth and provide strong evidence that the slab model near the array is too deep. This imprecision in the megathrust depth propagates into simulations and inversions that require a slab model; moreover, the megathrust depth defines the absolute distance from megathrust slip to population centers at the surface, which has direct implications for ground motion amplitude^30^. The thickness of the slab crust is another important parameter because it serves as an architectural baseline with which to evaluate compositional differences between the slab crust and the slab mantle^22^. In some places, phase conversions of the same type from the slab interface and slab Moho are observable within the same event wavefield. In these cases, the conversion points are close enough to estimate the thickness of the slab crust using a single event, thus largely eliminating uncertainty due to event location. The distributions of possible crustal thickness from individual events are generally wide, but span between 6 and 10 km (Fig. 3b). These values suggest thicknesses greater than expected given measurements of pre-subducted oceanic crust^31^, but these results match other thickness estimates of the portion of the slab above the slab Moho^6,23^. These large measured thicknesses could be due to subducted sediments, the altering of material in the forearc crust at the interface, the underplating of slab material, or a combination of these processes, producing an apparently thicker crustal section. Overall, our results are most consistent with slab models that are slightly shallower than Slab2 and produce Moho-to-interface thicknesses that are larger than presubducted oceanic crust^4,26^.

**Fig. 5:**
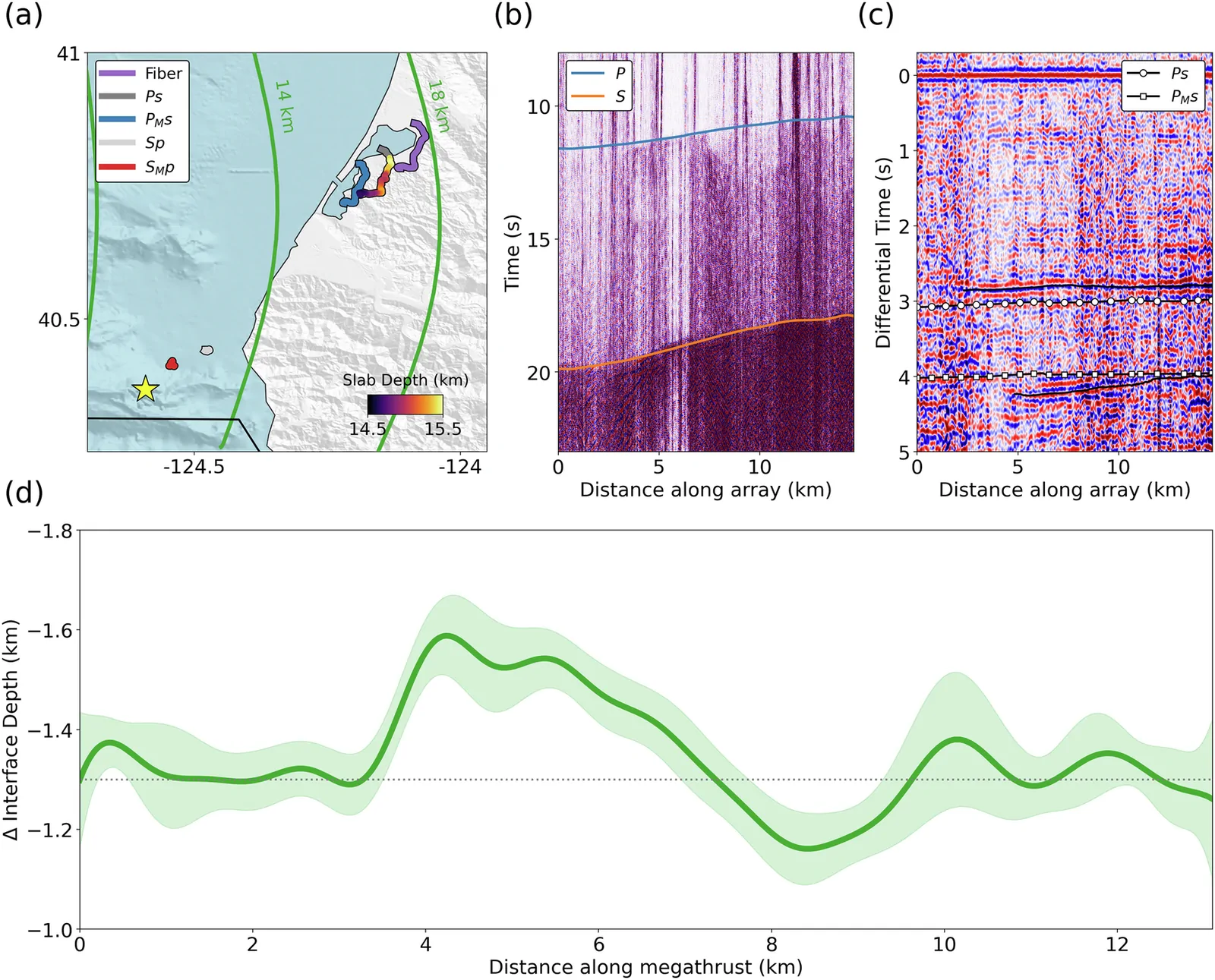
Inversion of short wavelength megathrust topography. **a** Map of earthquake epicenter (yellow star; nc75070761) and conversion points of different phases to slab structure at depth computed using the reference model. Included is the estimate of slab topography computed using the Ps conversion. **b** The earthquake wavefield for the event plotted in (**a**). **c** Stacked quasi-autocorrelogram with observed and computed arrival times for earthquake wavefield plotted in (**b**). **d** Transect, from north to south, along the Ps profile in (**a**), of necessary changes (△) of locally smooth slab geometry from Slab2^29^ needed to fit the Ps picks shown in (**c**). A negative value indicates depths shallower than Slab2. The differential time picks ranged from 2.7 to 2.9 s.

We next use very high-quality, determined visually, observations of Ps interface conversions to infer short wavelength topography over transects of the megathrust. Ps conversions are preferred for this application over Sp conversions because, for an individual event, they are sensitive to longer segments of structure at the slab interface (Fig. 5a). We again search a linear interpolant to determine slab depth, but here we interpret the measurements at each channel as being sensitive to narrow and distinct spatial envelopes at the slab interface as determined by 3D raytracing (see Methods), rather than combining them into a distribution. Together, these measurements provide a window into the short-wavelength topography of the megathrust fault at depth (Fig. 5). Though there are subsequently discussed tradeoffs with velocity structure and limitations of the raytracing approach, the results suggest that the megathrust over this segment has kilometer-scale bumps on the order of hundreds of meters in amplitude. In particular, the results point to the possible presence of a bump ~300 m high and 3 km wide. For comparison, we perform this inversion on another event with a well-resolved, but lower quality observation (Supplementary Fig. S2). We find that, although the conversion profiles are spatially separated, there is a similar bump in both profiles. For one, this suggests that this bump is not due to shallow structural effects because it is recorded by two events on different segments of the fiber (6–10 km along fiber for nc75070761 and 8–14 km along fiber for nc73947920). This is because raypaths from different events converge as they approach the array, so shallow structure beneath the fiber is sampled in common by both events and would impose a similar signature in each. This also suggests that the bump is elongated in the down-dip direction and may have a significant footprint on the slab. Resolving this feature requires precise measurements of relative differential times with sensitivity across the bump. Other Ps observations, while not inconsistent with this feature, are not extensive enough to corroborate it (Supplementary Fig. S3). Similar structures have been observed in high-resolution, offshore active source profiles as well. For example, an active source profile off the coast of southern Oregon shows a narrow deepening of the plate interface^5^ with a similar wavelength and amplitude to the bump observed in this study (Supplementary Fig. S4). This shows that these types of structures are present in images made using independent techniques and suggests that the bump we observe in our study is not unusual in Cascadia.

The profile shown in Fig. 5 has sensitivity to a narrow portion of the locked zone of the southern Cascadia megathrust (Fig. 1). More extensive observations within the locked zone and comparable observations outside the locked zone are needed to determine if and how structural variability in this region may be connected to megathrust locking. This is possible with a larger array aperature and would provide insight into the structural controls on megathrust locking. This inversion makes clear that, with a sufficiently accurate velocity model, fiber arrays can be used to image sub-kilometer megathrust topography at deeper depths than are accessible with standard techniques. The high precision of these measurements has historically been difficult to achieve, even with high frequency converted phase measurements, because high uncertainty in phase picks and hypocenter location uncertainty necessitate dense array observations to resolve short-wavelength changes in relative depth. The ability of DAS systems to record complicated strain wavefields that make clear peaks in correlograms and to measure wavefields from individual earthquakes over many kilometers means that this level of precision is feasible for large portions of megathrust faults.

### Challenges and uncertainties

In this study, we make observations of secondary amplitude peaks and attribute them to conversions related to slab structure. There are some potential sources of nonuniqueness that require assumptions to resolve. The first assumption is that these phases are related to slab structure, rather than other structures at similar depths. This assumption is well-justified. The slab is a first-order structural feature that imposes sharp impedance contrasts at depth. We also have estimates of the approximate depth of the slab and slab crustal thickness, and 3D raytracing suggest that these conversions all happen within a few kilometers of either the expected slab interface or slab Moho (Fig. 3). We also assume that the effects of seismic anisotropy, which could produce small shifts in Ps arrival time on a single horizontal component, are minimal. When we observe a secondary phase from the interface, we assume it is the direct conversion rather than a multiple within the slab crust. This is justified by the expectation that, while not always true, energy partitioning generally causes amplitudes of multiples to be smaller than the direct conversion^32^. We can easily distinguish between slab interface and slab Moho phases, because S_M_p arrives much earlier than Sp, and P_M_s arrives much later than Ps. This requires the assumption that the errors in Slab2 are not very large (lower than the slab crustal thickness), which is reasonable given the model’s estimated errors^29^ and is consistent with the proximity of expected arrival times from Slab2 to many of the phases observed in our correlograms (Fig. 3 and Supplementary Fig. S5).

The classification of the sense of conversion, Ps or Sp, is sometimes more challenging. In many cases, the expected arrival times of these two phases are very different, and the classification is easily made by evaluating which expected arrival time more closely matches the data. If the expected arrival times are similar, the array-side moveout of the observed phase compared to the expected phase arrivals may be used to aid in classification. This would be a stronger constraint for longer arrays (ours is ~15 km). If the expected moveout is also similar, source-receiver distance can also be used to determine whether a phase is unlikely to be Sp, because Sp is inferred to be weak at source-receiver distances beyond 60 km (Fig. 3). These methods of distinction cover all cases included in this study, but in future studies, additional methods for distinguishing between phases could be used. These could include using a priori knowledge of the geology and seismic velocities to anticipate the expected phase polarity, using collocated 3-component measurements to observe the phase polarization (Fig. 4), or using source-side beamforming from nearly colocated earthquakes^13^.

An additional challenge is determining the nature of the converting boundary at the slab interface with precision. In this study, we use high-frequency measurements with sub-kilometer wavelengths. We can theoretically image multiple boundaries near the slab interface and within the slab crust if there is finite separation at depth on length scales greater than the seismic wavelength and both boundaries efficiently produce converted phases. The slab crust is overlain by sediments of variable thickness (0–2 km)^25^, and the interface expectedly has finite width from accretion and alteration. We observe convincing evidence of multiple phase arrivals of the same type from nearby boundaries in the largest intraslab earthquake in our dataset (Fig. 4). The frequency of this conversion is >6 Hz, and the corresponding wavelength of the converting S-wave is <750 m, which corresponds to the approximate length-scale of sensitivity. This earthquake is the only event in our dataset during which there was a collocated 3-component nodal array, and we can confirm both phases are Sp conversions using phase polarization. The phase classification, relative polarity, and phase differential time information place a strong constraint on models attempting to reproduce these data. One way to fit these data is to construct a model that emplaces a low velocity zone at the slab interface, with lower velocities than the slab and higher velocities than the forearc crust. In this model, the variable differential time would suggest large changes in thickness of this weak zone over a narrow length scale (several kilometers). Determining the nature of this layer is challenging, and there are no comparable examples of this in this dataset. This layer suggests that with the right dataset, fiber arrays can be used to study layers at depth with thicknesses on the order of those of megathrust fault damage and alteration zones. In general, when looking at receiver function studies that image both the top of the oceanic crust and a shallow layer interpreted to be the subduction interface^4^, our results are more consistent with the interface depths.

Other sources of nonuniqueness in this study are due to hypocenter location uncertainty and velocity model uncertainty. Different phase differential times have different sensitivities: P-Sp differential times are more sensitive to the path near the source, and P-Ps differential times are more sensitive to the path near the receiver. For this reason, P-Sp differential times are highly sensitive to uncertainty in the hypocentral location, with the depth uncertainty being particularly important. This form of uncertainty is variable, and while hypocenter depths from the Northern California Seismic Network (NCSN)^33^ catalog are generally reported as being less than 1 km (Supplementary Fig. S6), the expected depth uncertainties are possibly underestimated^34^. The uncertainty in hypocentral depth for events with Sp conversions can, to first order, be mapped directly into the uncertainty for the slab depth (Supplementary Fig. S7). Relatedly, P-Ps differential times are highly sensitive to the uncertainties in the Vp/Vs ratio in the forearc crust. We perform our inversion using 3D raytracing using a velocity model with a well-resolved Vp/Vs ratio, but this model has poor resolution in the shallowest ~5 km^22^. The Vp/Vs ratio and the slab depth trade off with each other, but very large perturbations to the Vp/Vs ratio are necessary to account for the differences in slab depth we resolve with our inversion. For example, we resolve a large change in the absolute slab depth in Fig. 5 (~1.3 km). To get this change in the absolute slab depth, the Vp/Vs ratio would need to be inaccurate by ~10% throughout the entirety of the shallowest 5 km of the crust (Supplementary Fig. S8). The roughness measured on the megathrust fault in Fig. 5 is of lower amplitude than the absolute change in slab depth (~300 m). To produce this peak, there would need to be a sharp-gradient inaccuracy in the relative Vp/Vs ratio along the array. This inaccuracy would likely need to be kilometers deep and narrow (Supplementary Fig. S8). This is conceivable, but given that our observations can be verified across events and at different channels (Supplementary Fig. S2), we prefer the interpretation that the most prominent features are indeed slab topography. This is also indirectly supported by the observation that shear velocity heterogeneity in the shallowest 1 km beneath this array is not correlated with the perturbations in the Ps differential times that inform the bump^35^. Uncertainty in absolute slab depth can map into uncertainties in relative slab depth, because the absolute slab depth controls the locations and incidence angles of the conversion points. However, these effects are minor and do not change the results of this study (Supplementary Fig. S5). These tradeoffs show that short-wavelength slab topography, which may be important for the next generation of slab models, is best resolved with accurate and precise 3D velocity models with sensitivity to the full crustal section. In some cases, uncertainty can arise due to the picks themselves. While picking correlation peaks is highly precise, for the weakest differential time peaks, for example Sp in event nc73947930 in Fig. 3, there is some ambiguity in whether we are picking the true correlation peak or an adjacent side lobe. In these instances, there is generally a clear candidate for the true correlation peak based on the amplitude of the peak and the symmetry of the side lobes about the peak. While the relative differential times within the event are minimally affected, this ambiguity can lead to uncertainty in the relative polarity of the secondary phase and the absolute differential time, generally by half the period of the secondary phase.

The inversion for slab topography was performed using a linear interpolation function defined by computing phase differential times for several slab interfaces, offset by absolute values. This inversion is an approximation, performed for computational reasons, that fails to account for ray-bending effects caused by short-wavelength interface topography. Ray-bending has a smoothing effect on the differential time measurements. For bumps that cause shallowing of the slab interface, rays are preferentially directed to the bump to minimize the travel time, resulting in a bias in our inversion towards an artificially wider bump. For bumps that cause a relative deepening of the slab interface, rays preferentially avoid the bump, resulting in a bias in our inversion towards an artificially lower amplitude bump. The effects of these biases are mild, and examples are shown in Supplementary Fig. S9. With greater computational resources, these biases could be avoided by performing an iterative inversion for the full interface, rather than inverting for slab depth for each channel individually. Additionally, the time-minimization geometric raytracing that we use is an approximation that does not include finite-frequency effects. To partially account for the finite bandwidth of the wavefield, we smooth the inverted interface by the wavelength of the converted phase in the neighborhood of the conversion. But this approach, which is a pragmatic simplification of commonly used methods in tomography^36^, can be inaccurate in the presence of strong structural heterogeneity. In some cases, structural complexity sensed over a finite bandwidth can shift the distribution of sensitivity away from the time-minimization path. This problem is especially acute when the interface topography varies at scales similar to the converted phase wavelength of 750 m^37^. For this reason, we are careful to only interpret structures that are several multiples larger than this scale. Using Sp and Ps finite-frequency kernels would produce a more accurate result^38^. Frechet kernels for Sp and Ps typically have high and uniform amplitude over scales of a few kilometers^39^. The computational expense of computing these kernels at the frequencies used in this study is large and we do not yet have an available high-resolution starting velocity model.

### Broader implications

This study was performed using a relatively short fiber deployment (~15 km). There are several scientific problems that could be pursued if the methods developed in this study are applied to a larger aperture array. As is shown in Fig. 1, the expected locked zone of southern Cascadia is coincident with our array, and we measure roughness on the megathrust within the locked zone (Fig. 5 & Supplementary Fig. S2). A larger array could help relate roughness to megathrust locking by allowing for the imaging of transects of interface topography within locked and creeping sections. The results shown in Fig. 4 suggest a connection between the locked zone and the hydration of the slab, but the analysis is limited by the fact that few observations are outside the locked zone. The connection between slab hydration and megathrust locking could also be further clarified by more extensive and diverse measurements of phase conversion polarity and amplitude. These outstanding problems could be addressed in this region following an expansion of the fiber array used in this study that leverages a larger portion of the Middle Mile Broadband Initiative fiber (Supplementary Fig. S10)^40^.

Moreover, there are thousands of kilometers of onshore and offshore fibers in subduction zones that could potentially be transformed into DAS arrays, several of which are already being sensed or have been in the past^41–44^. This technique can be applied wherever there are intraslab earthquakes and structural discontinuities at the slab interface, which is not unique to southern Cascadia. These phases have been observed in Alaska^45–48^, Mexico^49,50^, Japan^51–54^, New Zealand^55,56^, and other segments of Cascadia^57,58^. This means that repeatable high-frequency measurements of the slab interface can be made at depth in subduction zones with diverse geology and phenomenology. In regions with observed megathrust earthquakes or slow slip events, this technique could resolve structure in deeper parts of subduction zones at high frequencies, like that shown in Fig. 5, towards better understanding how fine-scale structure affects slip dynamics. High-frequency measurements made over long, down-dip subduction transects can inform how the subducting oceanic stratigraphy and the finite fault interface, as were observed in Figs. 3 and 4, evolve throughout subduction^59^. Anchoring subduction interface depth using high-frequency converted phase measurements enabled by DAS, as shown in Fig. 3, can minimize inaccuracies in simulations and inversions that rely on slab interface depth as a fundamental parameter. In any case, this study shows that fiber-optic arrays can play an important role in the future of subduction zone science by providing a window into fine-scale slab structure at depth.

## Methods

### GorDAS array and earthquake observations

In May of 2022, a DAS array was deployed along ~15 km of telecommunications fiber between Arcata and Eureka, California. This array was deactivated in September of 2022 and was subsequently redeployed three days after the December 20, 2022 M_w_ 6.4 Ferndale earthquake and remains operational. The data were initially recorded at 250 Hz with 2 m channel spacing and 8 m gauge length. The sampling rates were changed to 100 Hz and 5 m channel spacing in early 2023. This array has recorded over 200 M2.5+ earthquakes from the NCSN catalog^33^ within a distance of 100 km. We visually evaluated each of these earthquake wavefields, and we selected 60 earthquakes with clear P-wave onsets and little traffic noise as template events to be used in the subsequently described methodology. We also selected 23 earthquakes with observable P-wave onsets and hypocenter depths >10 km below the Slab2 interface^29^ as candidate wavefields for converted phase observations (Supplementary Table S1). We chose 10 km to ensure the events were deep enough such that the observability of converted phases could be expected. That is, the absence of converted phase observations for these events would also be meaningful. At these depths, the converted phase onsets should be well-separated from the first arrival, even in the presence of depth uncertainty of the event hypocenter and Slab2 interface.

### Quasi-autocorrelogram stacking

In this study, we build on the approach for picking differential times between DAS-measured phases developed in Atterholt and Zhan^15^. In their study, it is recognized that the first arriving phase and secondary phases generate similar scattered wavefields due to shallow heterogeneities near the array. Thus, correlating a window around the first arriving phase with the earthquake wavefield generates correlation peaks at the differential times between the first arrival and secondary phases. In their study, they apply this technique to the Moho reflected phase, but we show that this technique is generalizable to phase conversions as well. The correlation-based approach produces highly precise differential time picks along a well-sampled spatial axis from a single event, avoiding relative event location uncertainty. Moreover, even if the polarization of the incoming converted wave is oriented at an unfavorable angle relative to the cable azimuth, the locally generated scattered energy will likely include some energy that propagates along the cable azimuth and be well recorded^60^.

Since the slab phase conversions in our dataset are often weak, we apply a stacking procedure to improve the signal-to-noise ratio of these correlation peaks. Noting that the scattered wavefield is generated locally^20^, distinct events should generate similar scattered wavefields. After applying spectral whitening and bandpass filtering the data between 1 and 10 Hz, we take a 2 s window around the computed first arrival, which is shifted by visual inspection if necessary, of each of our template events and candidate events and use these as scattered wavefield templates that we correlate with the candidate earthquake wavefields. We specifically window around the first arrival to amplify the correlation peaks with the P-wave, even if the P-wave is weak or emergent. The choice of 2 s balances our intention of capturing the full complexity of the scattered wavefield associated with the first arrival while minimizing the contribution of later, stronger scattered waves. We then align these correlograms by the peak associated with the correlation of the direct P arrivals, corresponding to the zero-phase peak in an autocorrelogram. To achieve this alignment, we visually identify zero-phase peak and manually choose a narrow time window that encompasses this peak. We then pick the zero-phase peak times by selecting a maximum (or minimum) within this window. If the correlation between P-waves is weak, we do not use the template. If this zero-phase time is symmetric around a negative peak, we assume this is due to source radiation differences and multiply the correlogram by negative one. We term these aligned correlograms “quasi-autocorrelograms,” because they approximate an autocorrelogram using similarly scattered wavefields from distinct events. We stack these quasi-autocorrelograms to produce what is generally a much clearer picture of the differential phase and relative polarity of secondary arrivals (Fig. 2).

### Computing expected arrival times and synthetic waveforms

We use our converted phase measurements to infer absolute and relative slab depth. Using an accurate 3D model is thus important for correcting for lateral heterogeneities in velocity structure when computing expected arrival times. We use the model from Guo et al.^22^, because this model resolves high spatial frequency velocity structure in our study area with both offshore ocean bottom seismometers and onshore permanent and temporary network stations. We apply a raytracing approach similar to that of Gong and McGuire^13^, which employs the pseudo ray bending method employed in TomoDD^61,62^ in a grid-search approach for the conversion point that minimizes the travel time of Ps and Sp converted phases. That is, for each source, we compute the P and S travel times from the source location to a grid of points with 10-meter spacing on the Slab2 model^29^, and we also compute the P and S travel times from each grid point location to the station locations. The grid is computed within a narrow spatial window selected using expected arrival times computed over a coarser grid with 1-km spacing. The grid points that minimize the travel time of the P-wave to the grid plus the S-wave from the grid and the S-wave to the grid plus the P-wave from the grid yield approximate Ps and Sp converted phase travel times, respectively. This technique is generalizable to other phase conversions, and we use it to determine the expected arrival times of slab Moho conversions by applying the same workflow to grid points on an interface 7 km deeper than the Slab2 interface.

For the synthetic waveforms, we are most concerned with determining how variant models produce first-order differences in converted phase waveforms, and thus 1D synthetics are sufficient for our purposes. Each model is defined using 1 km-thick layers, a uniform attenuation structure (${Q}_{\alpha }=2{Q}_{\beta }=1000$), and a delta source time function. We parameterize four velocity models that are a modification of a 1D average velocity model for the region^22^ and an assumed Vp/Vs ratio of 1.73. i. Slow hydrated forearc: Vp is increased by 10% in the slab and decreased by 12% in the forearc crust; Vp/Vs is increased to 2.1 at the base of the forearc crust and decreases linearly in the overlying 7 km of crust until it returns to 1.73. ii. Slow forearc + hydrated slab: same as (i), but Vp/Vs is instead increased to 2.1 at the top of the slab and decreases linearly in the underlying 7 km of the slab until it returns to 1.73. iii. Fast forearc + hydrated slab: original 1D model is preserved, but the Vp/Vs ratio is increased to 2.2 at the top of the slab and decreases in the same way as (ii). iv. Slow forearc + low velocity zone: same as (i), but Vp of a kilometer-thick layer above the slab interface is decreased by only 7% and the increase in Vp/Vs ratio begins above this layer. Though slightly exaggerated, these Vp and Vp/Vs contrasts are of the same order as observed in refraction results and travel-time tomography^22,26^. These models and their Vp/Vs ratios are shown in Fig. 4 and Supplementary Fig. S11, respectively.

For these synthetics, we use the Frequency Wavenumber Summation method^63^ to generate waveforms and the TauP raytracing software^64^ to determine travel-times for converted phases in our 1D model. We use the autocorrelation approach described above on the synthetic data for direct comparability with the DAS autocorrelogram. We additionally use PyKonal^65^, an Eikonal equation solver that employs the Fast Marching Method^66^, to perform travel-time sensitivity tests of the Ps (Sp) phase to simple variations in slab interface morphology and velocity heterogeneity. For these tests, we determine Ps arrival times by defining a model with P velocities below the interface and S velocities above the interface such that the time minimization path is equivalent to the Ps path through a standard model. For the Sp arrival times, we do the same but with S velocities below the interface and P velocities above the interface. For the Sp test, we ensure the source-receiver distance is sufficiently low such that the transmitted phase is the first converted Sp arrival. The model we use is the same 1D average model perturbed to generate the synthetic waveforms^22^ with an initial Vp/Vs ratio of 1.73.

### Slab depth inversion

To invert for slab depth, we compute the expected P-Sp and P-Ps differential times for slab interface depths that are equal to, 3 km shallower, and 3 km deeper than the Slab2 model for each event. We use these computed differential times to define a linear interpolation function between absolute slab depth and phase differential time at each conversion point. Inversions for absolute slab depth are performed for each differential time measurement by mapping the observed phase differential time into absolute slab depth using these linear interpolants. When looking at slab topography using a single earthquake wavefield, as shown in Fig. 5, we apply a spatial smoothing corresponding to the expected wavelength of the phase conversion at the slab interface. This spatial smoothing is accomplished by fitting a Gaussian process to the data with a prescribed length scale equal to the wavelength, which we define to be 750 m (assuming a shear wave velocity of ~4.5 km/s near the interface and a frequency of 6 Hz). That is, we impose a spatial correlation length equal to the expected wavelength, consistent with the intrinsic resolution of the converted phase. Prescribing this length scale allows the Gaussian process to pool information across nearby picks, reducing the posterior uncertainty below the per-pick uncertainty. The standard deviation on the picks, which constrains the Gaussian process error, is assumed to be 1/4 the period of the correlation peak (assuming a frequency of 6 Hz) for the best constrained picks (nc75070761) and 1/2 the period of the correlation peak for the more poorly constrained picks (nc73947920).

## Supplementary information

Supplementary Information

Transparent Peer Review file

## Data Availability

The waveform data are available via the USGS data release of McGuire et al.^16^. Maps included in this publication are made with Natural Earth data. Topography used in maps is the global Earth relief dataset from the Institute of Geophysics and Planetary Physics (IGPP)^67^.

## Code availability

Code used for pseudo-raybending technique is available at https://zenodo.org/records/5547889^68,69^. The Frequency Wavenumber Summation method^63^ implementation is available at https://github.com/ziyixi/pyfk^70^. TauP is available through Crotwell et al.^64^. Pykonal is available through White et al.^65^.
