# Supplementary material for: Sharp structural variability of the Gorda slab imaged by a fiber array
Source: Nat Commun. 2026 Jul 25;17:9072. doi: 10.1038/s41467-026-76002-8 (PMC13518939; doi:10.1038/s41467-026-76002-8)
Supplement: Supplementary file 1 [file 41467_2026_76002_MOESM1_ESM.pdf]

Supplementary Materials for Sharp Structural Variability of the Gorda Slab Imaged by a Fiber Array

J. Atterholt[1*], J. J. McGuire[2], A. J. Barbour[2], and M. P. Moschetti[1]

[1]U.S. Geological Survey, Geologic Hazards Science Center

[2]U.S. Geological Survey, Earthquake Science Center

Corresponding author: James Atterholt (atterholt@arizona.edu)

*Now at: University of Arizona, Department of Geosciences

Table of Contents:

- Figures S1-S11 – Pages 2-12
- Table S1 – Page 13

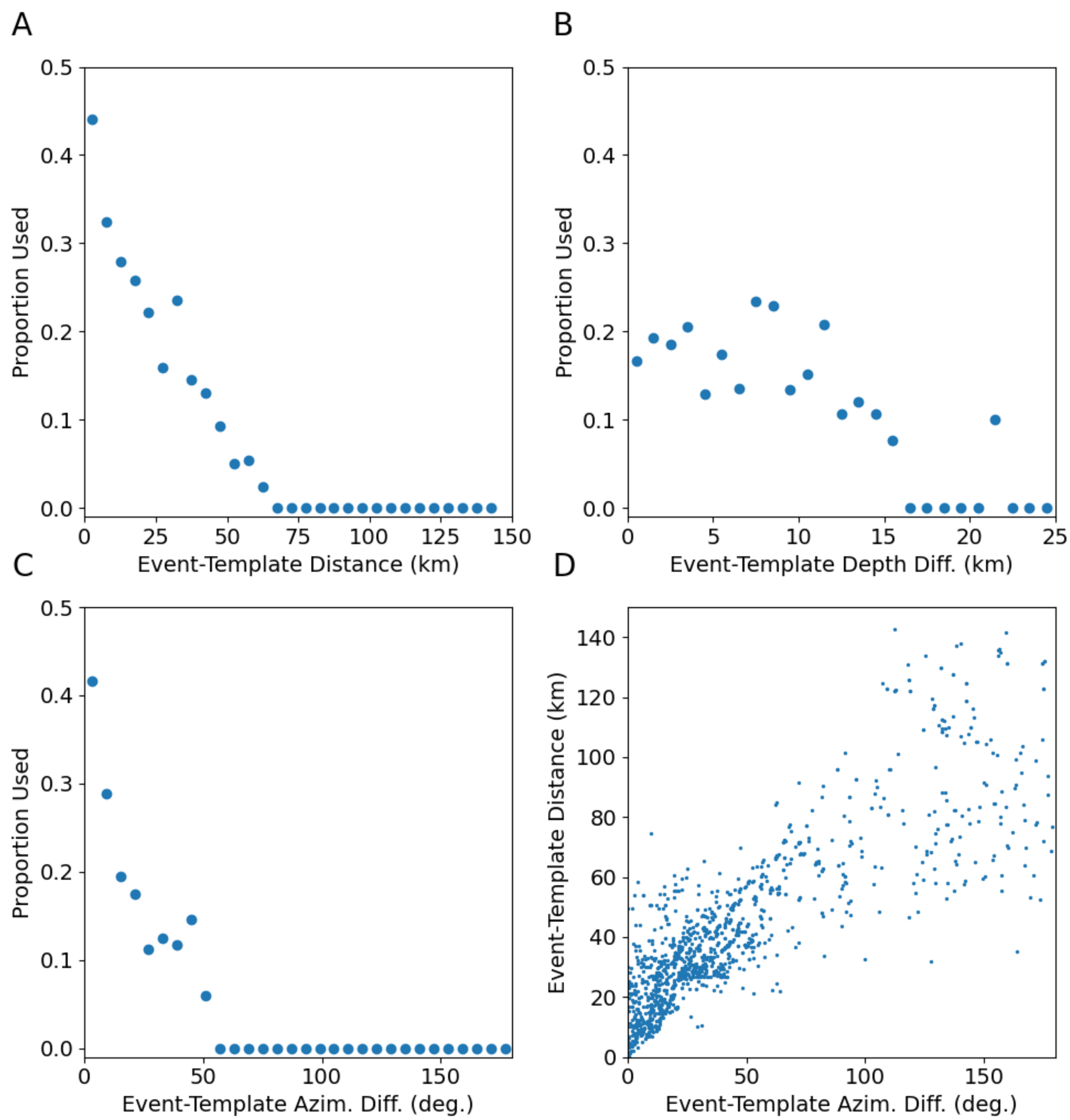

Figure S1. Evaluation of controls on quality of scattered wavefield templates. Whether a template was used for pseudo-autocorrelogram stacking or not was determined by visual inspection of the amplitude and continuity of the zero-phase correlation peak corresponding the correlation between first-onset P-waves. All 60 templates were tried on each candidate event. Panels A-C show the proportion of templates used for stacking as a function of (A) the lateral distance between event and template, (B) the difference (diff.) in event depth and template depth, and (C) the difference in the source-array azimuth (azim.) between the event and template. D. Scatterplot of lateral distance (from A) against the difference in the source-array azimuth (from C). The strong correlation between these two parameters explains why their respective trends in stacking proportion are similar.

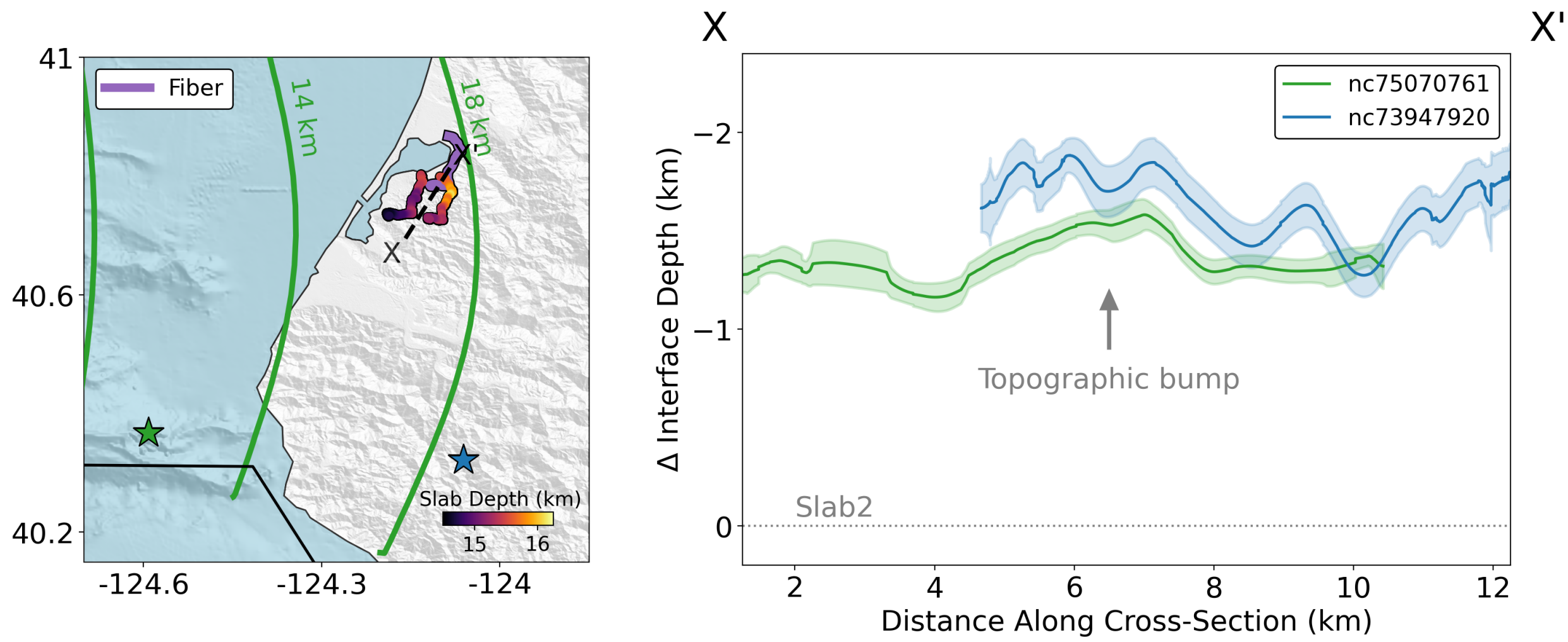

Figure S2. Comparison of megathrust topography profiles. Plot shows the slab interface inversion results using P-Ps differential times from different events. Colored stars on the map are epicenters of events used. The color of the star is the same as the color of the slab profile generated from the event wavefield. Results from each event are projected onto the same vertical plane and represented in cross-sectional view of change (Δ) in interface depth versus distance. There is a clear correspondence between the topographic bump between 4 and 8 km, suggesting both that this feature is not a site effect and that the feature is elongated in the down-dip direction.

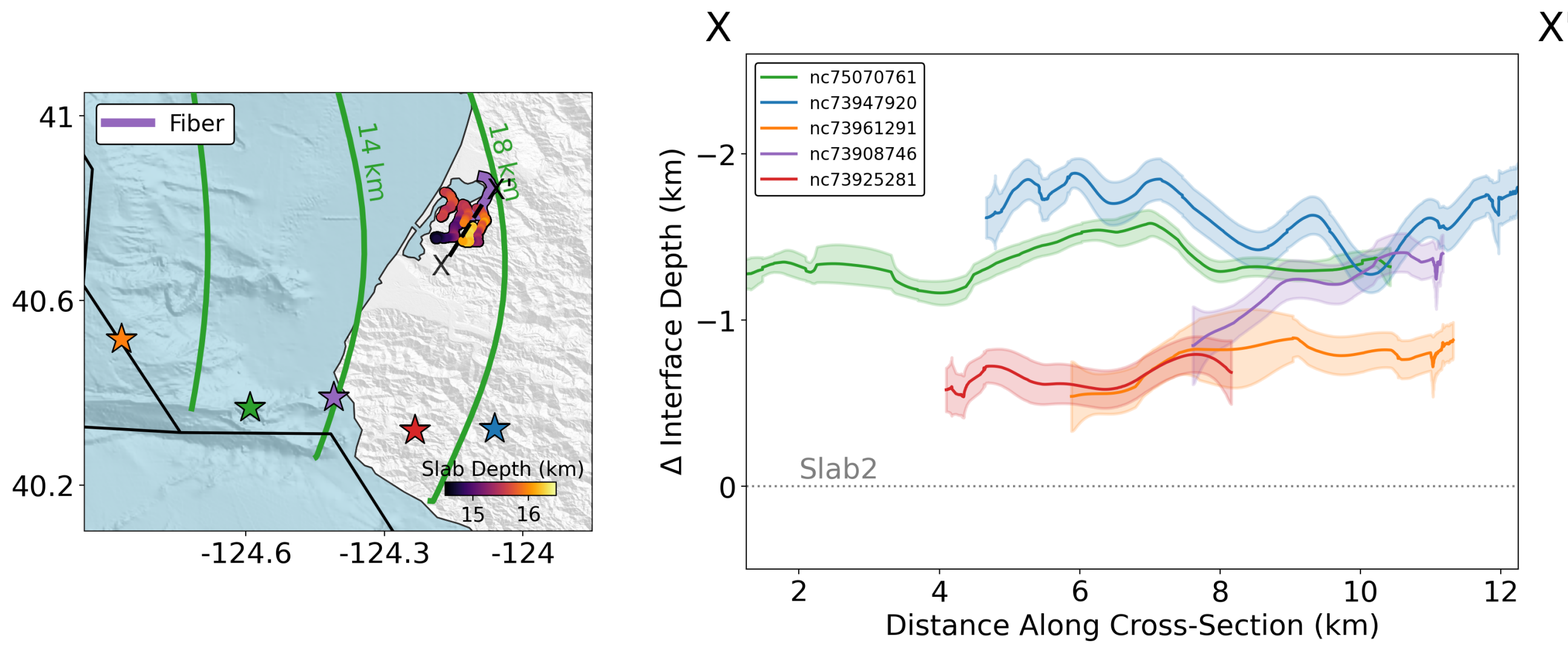

Figure S3. Comparison of profiles from all Ps observations. Same as Figure S2, but all Ps conversions represented in Figure 3 are included. Profiles from nc73961291, nc73908746, and nc73925281 are too short or distant to corroborate the bump resolved in nc75070761 and nc73947920. The error bars for these curves are computed for relative slab depths within each event profile and not for the larger absolute slab depth error, which in part accounts for large differences in the change in interface depth between profiles.

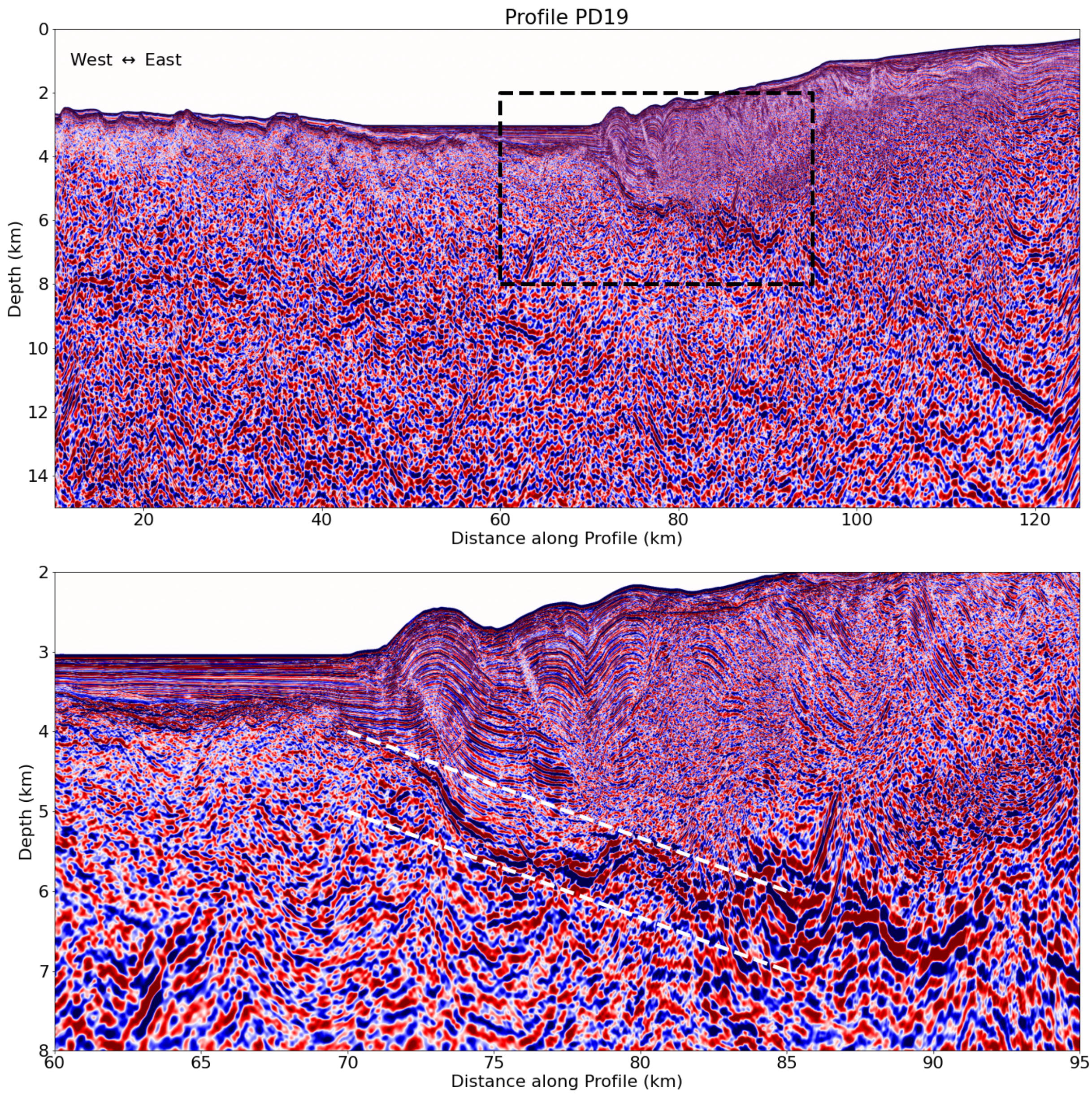

Figure S4. Short-wavelength interface roughness in seismic reflection data. Top: west-to-east depth-migrated reflection data from Profile PD19 in Carbotte et al. (2024)[5]. Black dotted rectangle outlines zoomed-in section shown in the bottom plot. Bottom: Zoomed-in view of the reflector associated with the plate interface in a zone of high interpreted interface roughness. Parallel white dotted lines, which are 1 km apart, bound a pronounced depression in the plate interface. Amplitude and wavelength of this depression are ~0.6 km and 6 km, respectively.

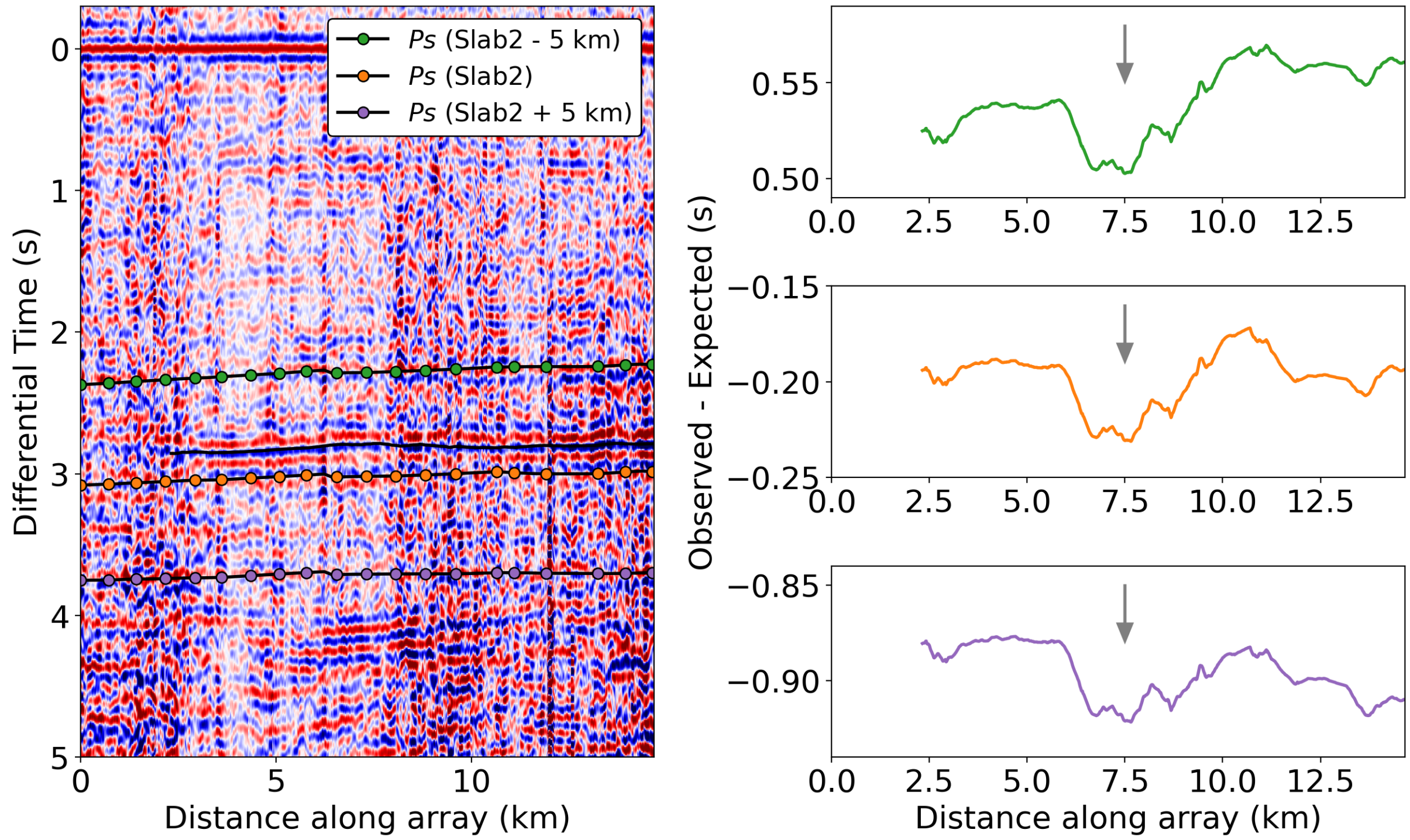

Figure S5. Representation of the difference between the correlogram peak picks and the expected arrival times for Ps conversions from Slab2, Slab2 made shallower by 5 km, and Slab2 made deeper by 5 km, computed using the 3D raytracing technique described in the paper. Left: the correlogram for nc75070761 with the expected arrival times of Ps for different slab depths plotted. The black line indicates the picks of the correlogram peaks. Right: representation of the differences between the Ps picks and the expected arrival times for Ps for the different slab depths. Colors correspond to the model changes indicated in the correlogram legend. The vertical scales of these plots are the same, but shifted. Note that the narrow, negative dip in differences between observed and expected indicated by the gray arrow is present regardless of the absolute Slab2 depth being used to compute the expected arrival times.

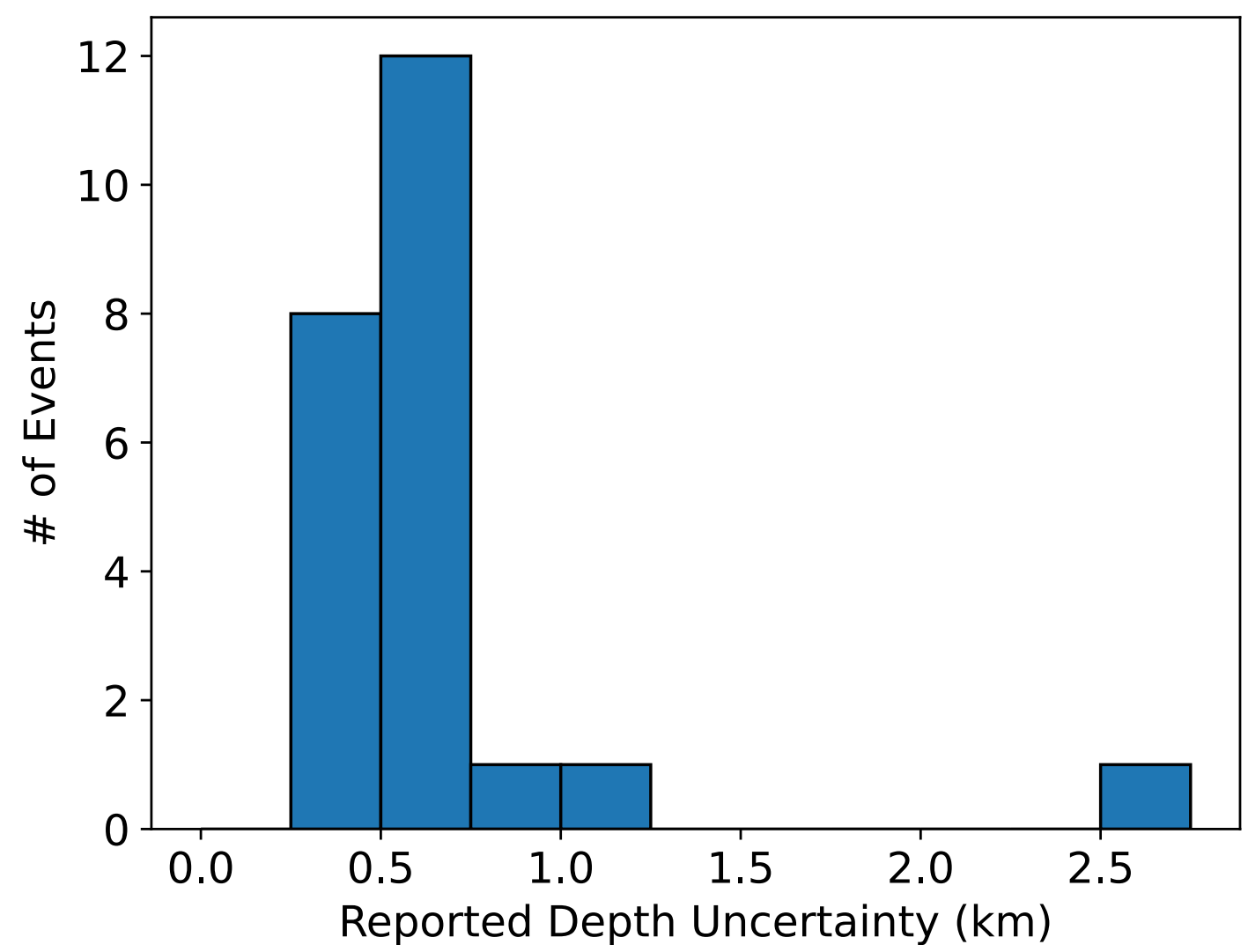

Figure S6. Depth uncertainties for intraslab earthquakes. Distribution of hypocenter depth uncertainties reported for the Northern California Earthquake Data Center[33] hypocenter locations for the intraslab events used in this study (Table S1).

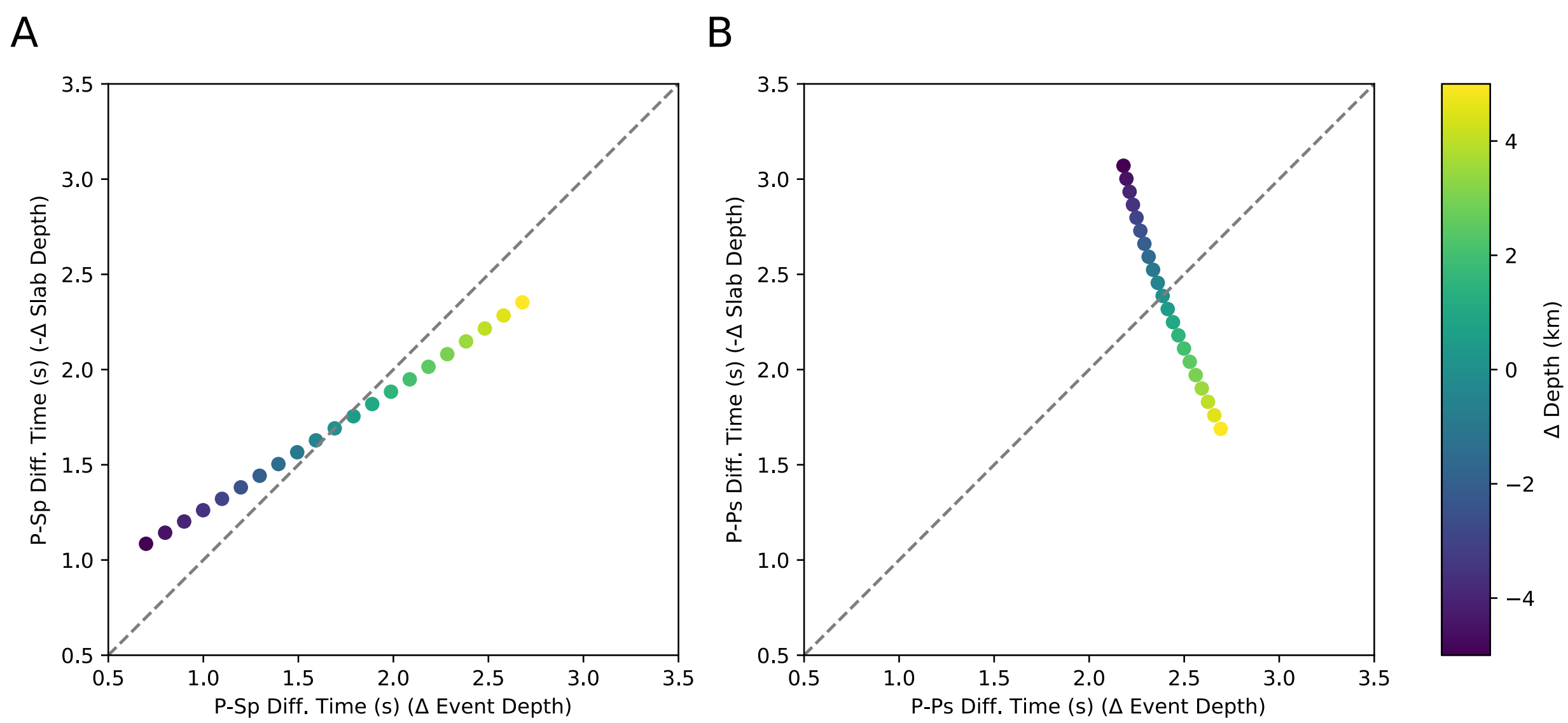

Figure S7. Comparison between the changes in differential time due to equivalent changes in event depth and slab interface depth. A. Compares the P-Sp differential times due to increasing hypocenter depth and due to decreasing slab depth by equal amounts. The axes represent the change in differential time due to changing (Δ) depths, and the colorbar represents the size of the change. This plot shows that for P-Sp differential (diff.) times, hypocentral depth and slab depth trade off strongly with each other. B. Same plot as in A but for P-Ps differential times. This plot shows that estimates of slab depth made using P-Ps differential times are not as strongly affected by hypocentral depth uncertainty as P-Sp differential times.

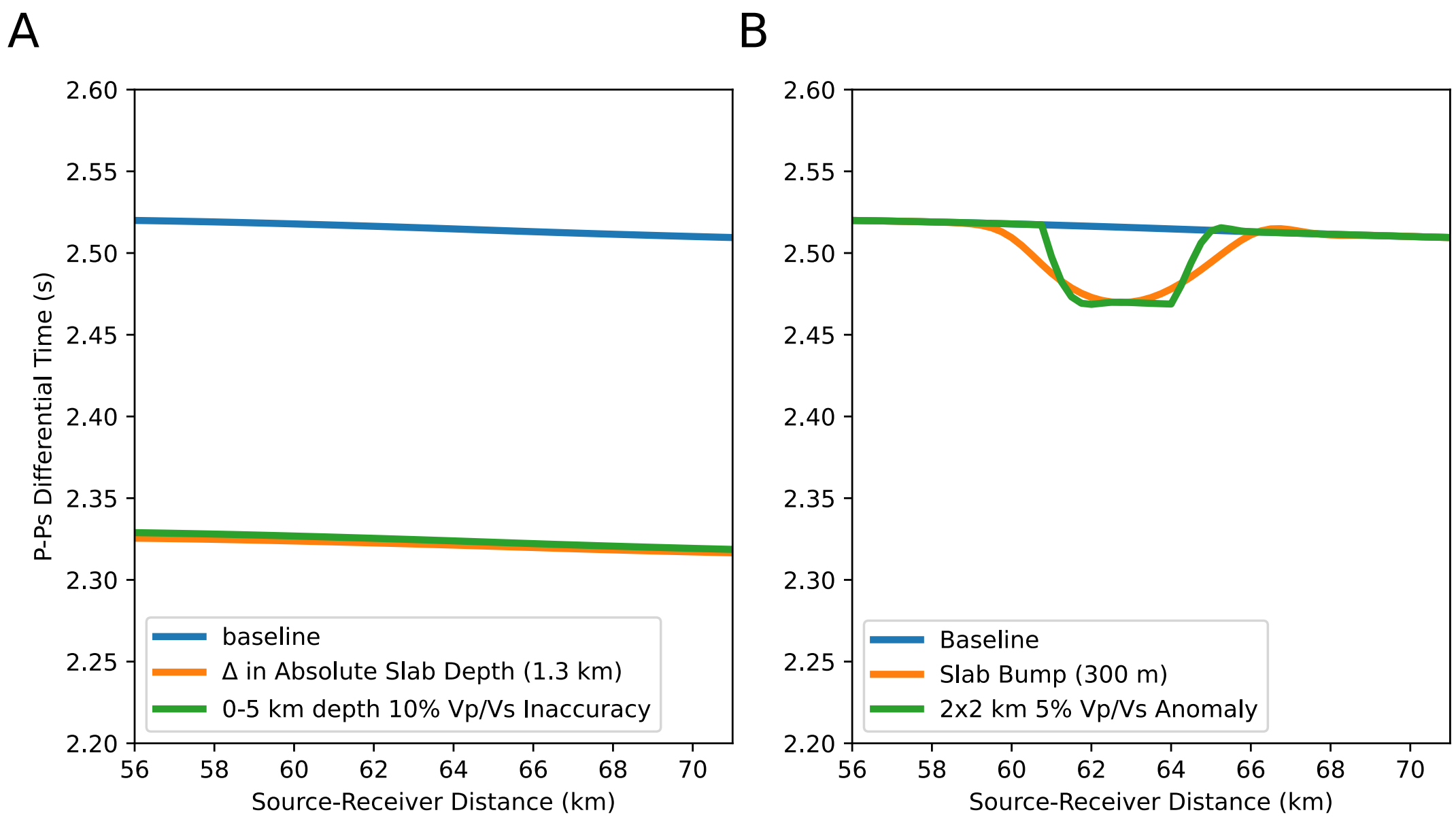

Figure S8. Sensitivity of absolute and relative slab depth estimated from P-PS differential times due to uncertainty in the V_p/V_s ratio of the 3D velocity model used for raytracing. A. Changes in P-Ps differential time due to a change (Δ) in the slab model comparable to what is observed in Figure 5 versus the effect of underestimating V_p/V_s of 10% through the the top 5 km of the crust. B. Changes in P-PS differential time due to a relative change in slab depth comparable to that observed in Figure 5 versus the effect of underestimating in V_p/V_s of 5% in a narrow and shallow portion of the crust.

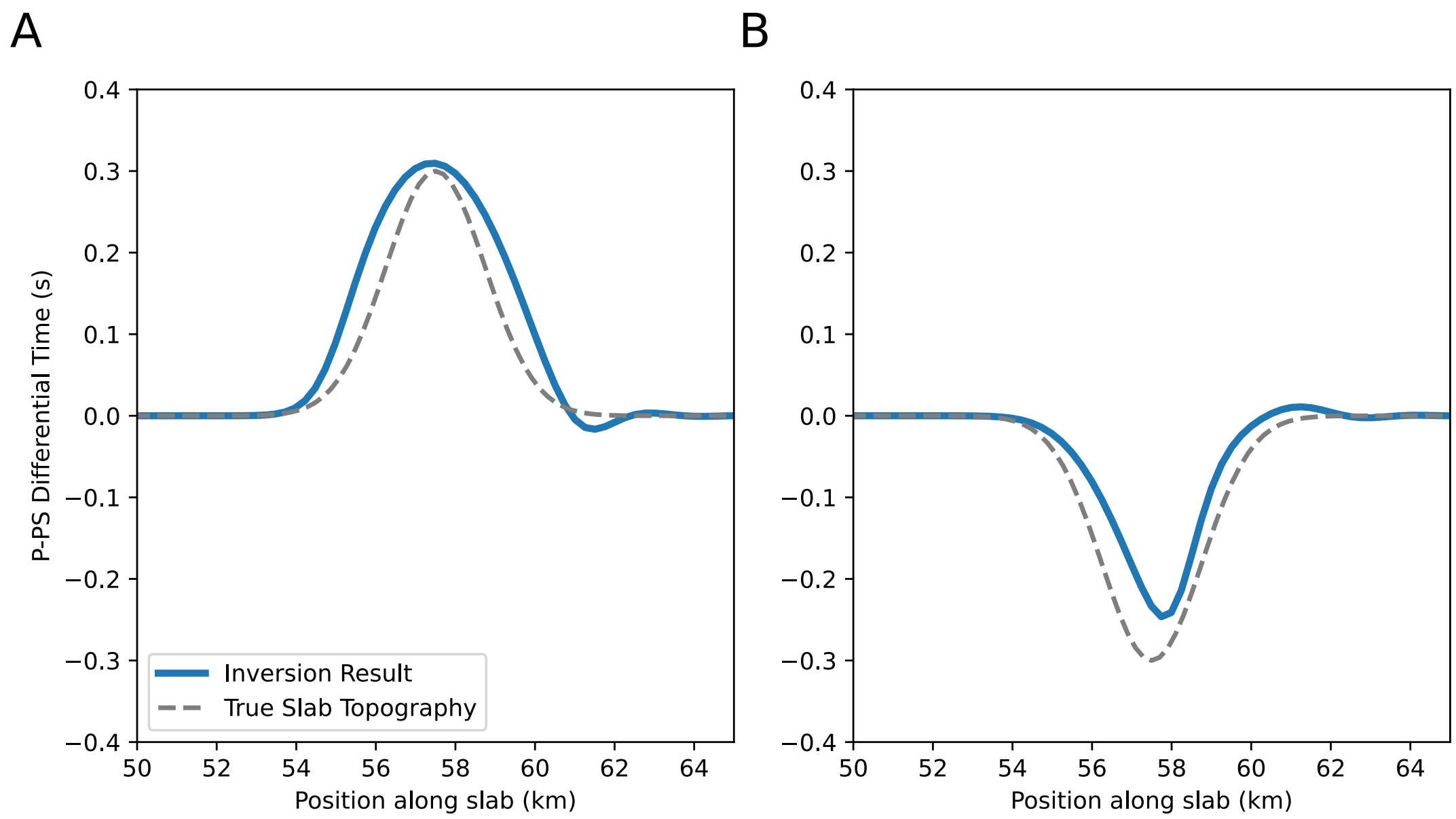

Figure S9. Test of sensitivity of the functional space inversion described in methods to positive and negative slab bumps. A. Inversion result for positive slab bump compared to true geometry. Notice how the inverted bump is slightly wider than the true bump. B. Inversion result for negative slab bump compared to true slab geometry. Notice how the inverted bump is slightly lower amplitude compared to true slab geometry. For these tests, the source depth was set to 26 km and the source-receiver distances ranged between 55 and 75 km.

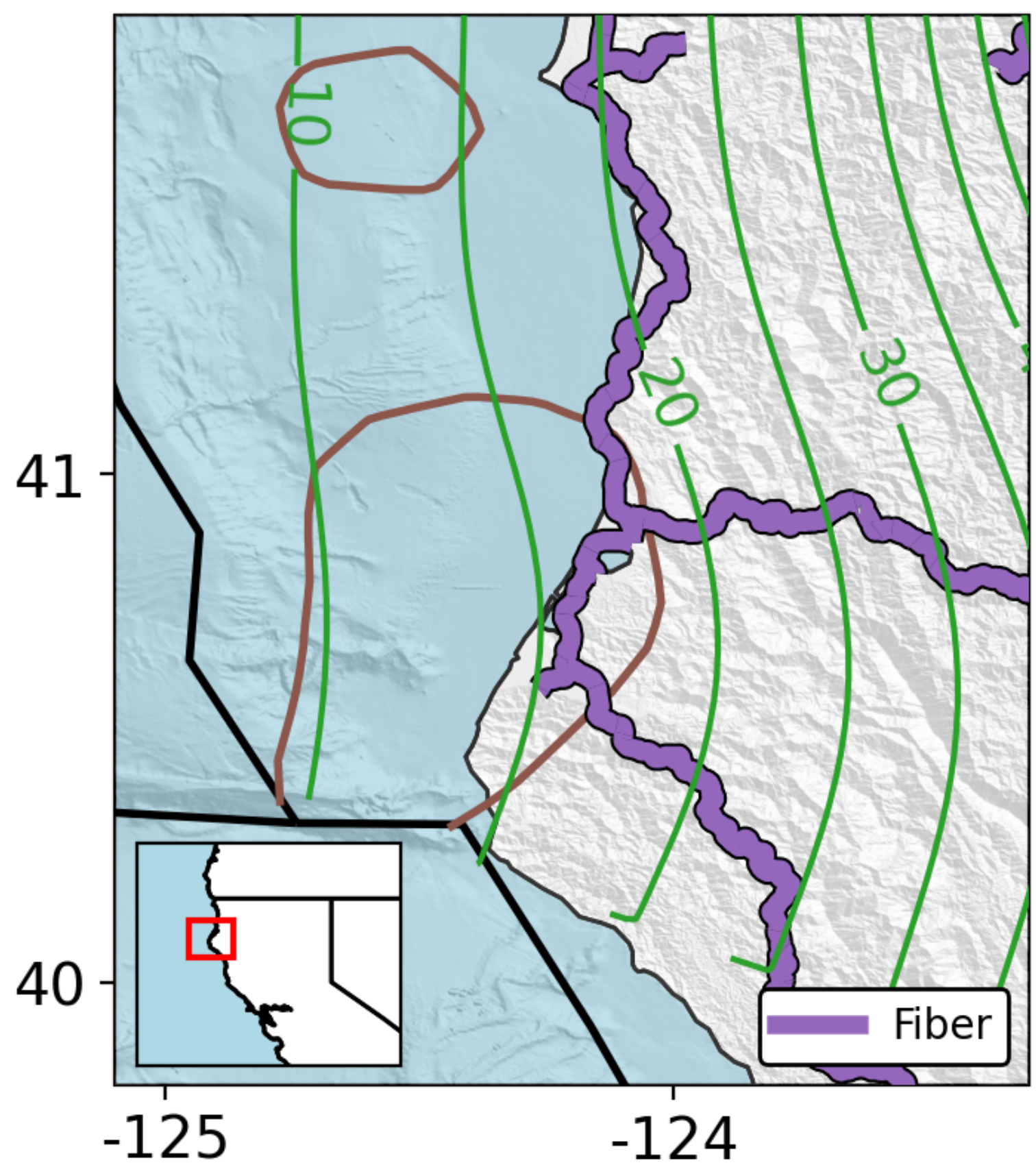

Figure S10. Regional coverage of Middle Mile Broadband Initiative fiber. Purple curve shows fiber geometry from the Statewide Network Map associated with the initiative[40]. Green contours represent the interface depths (km) of Slab2[29]. Brown contour encompasses zone in which the slip deficit exceeds 20 mm/yr[18].

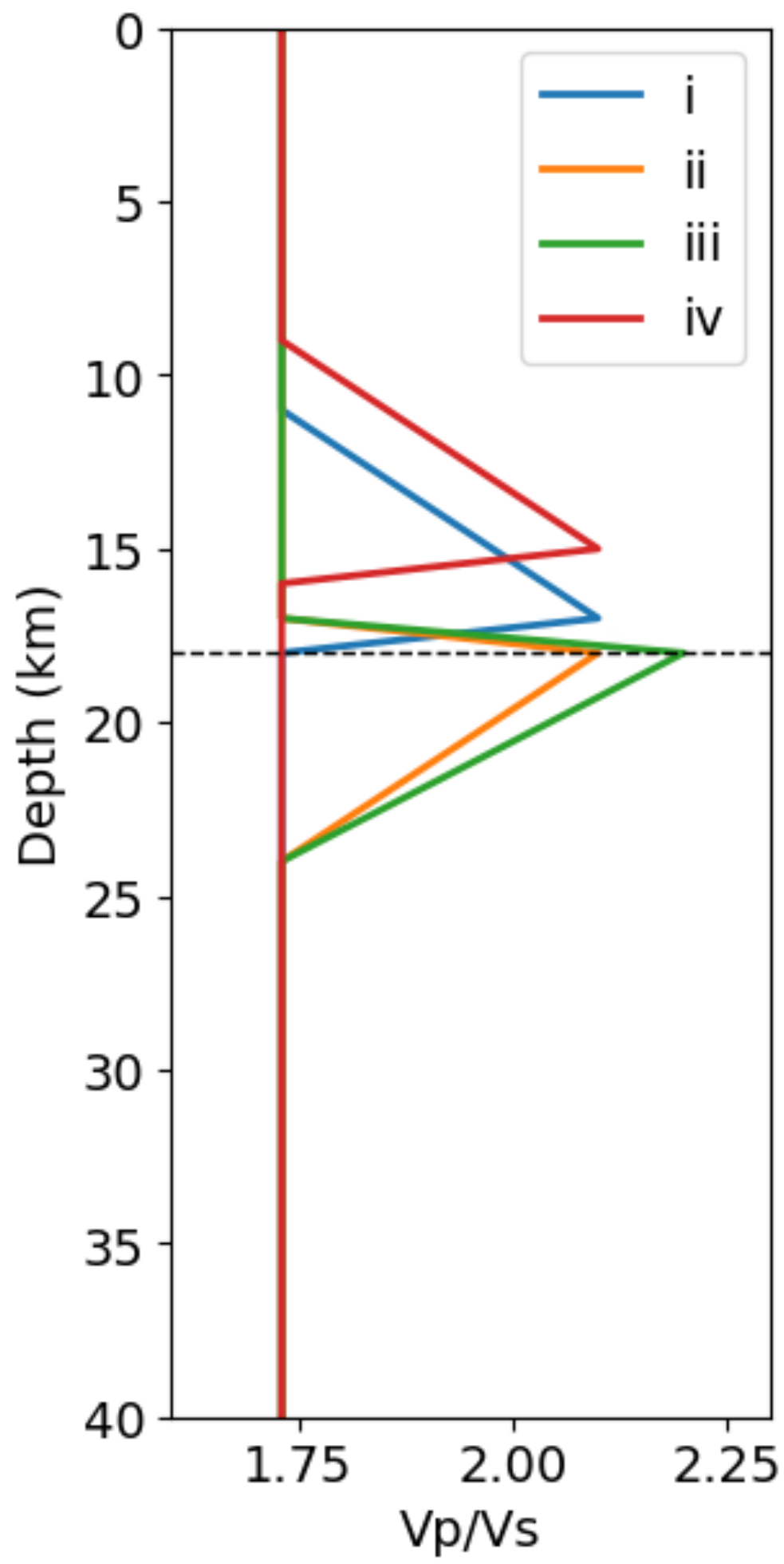

Figure S11. Vp/Vs ratios for models used in waveform synthetics. Legend indicates the corresponding 1D Vp and Vs models shown in Figure 4.

ID	latitude	longitude	depth (km)	magnitude	time
nc73824006	40.3745	-124.4985	23.07	2.87	2022-12-24T05:13:07.200000+00:00
nc73825241	40.501	-124.809	20.66	2.88	2022-12-26T12:20:54.970000+00:00
nc73827571*	40.409	-123.971	30.63	5.35	2023-01-01T18:35:04.510000+00:00
nc73838976	40.5355	-124.39733	24.7	3.7	2023-01-28T23:56:58.670000+00:00
nc73842546	40.540165	-124.489	23.88	2.88	2023-02-06T09:53:42.790000+00:00
nc73908746*	40.38983	-124.4095	29.86	3.88	2023-07-04T14:58:49.570000+00:00
nc73925281*	40.318333	-124.23434	31.93	4.07	2023-08-17T21:08:00.860000+00:00
nc73937486	40.72283	-124.607	21.19	2.63	2023-09-16T13:11:14.050000+00:00
nc73943846	40.501	-124.4385	29.09	4.22	2023-09-30T17:16:47.830000+00:00
nc73947930*	40.755	-124.31133	26.33	2.93	2023-10-16T14:15:42.130000+00:00
nc73947920*	40.321667	-124.061	31.59	2.98	2023-10-16T13:04:06.960000+00:00
nc73947835*	40.756	-124.52084	23.63	4.03	2023-10-16T10:31:25.060000+00:00
nc73947830	40.315666	-124.05483	31.06	4.83	2023-10-16T10:20:49.740000+00:00
nc73961291*	40.515835	-124.87067	18.68	2.8	2023-11-11T03:58:15.110000+00:00
nc73992726	41.499	-124.2015	35.68	4.33	2024-01-23T05:26:10.410000+00:00
nc73998256	40.470665	-124.51933	25.05	3.95	2024-02-03T20:14:14.570000+00:00
nc74001081*	40.4375	-124.37617	29.04	2.74	2024-02-11T10:39:31.880000+00:00
nc74040296	40.447	-124.755	21.39	3.1	2024-04-24T13:35:34.790000+00:00
nc75055336	40.467834	-124.70567	24.95	2.96	2024-08-30T17:13:03.020000+00:00
nc75070761*	40.367332	-124.59167	25.93	3.43	2024-10-05T16:24:35.160000+00:00
nc75082686*	40.497665	-124.806	18.98	3.1	2024-11-04T16:41:29.700000+00:00
nc75092706*	40.514	-124.29867	26.3	2.59	2024-11-30T02:24:33.480000+00:00
nc75096151	40.4815	-124.51083	25.15	3.58	2024-12-05T20:00:21.180000+00:00

Table S1. Source parameters for all intraslab earthquakes used in this study, organized by date. 'ID' is the event identification code from the NCEDC catalog[33]. Asterisks mark events for which converted phases were observed.
