# Supplementary material for: Sharp structural variability of the Gorda slab imaged by a fiber array
Source: Nat Commun. 2026 Jul 25;17:9072. doi: 10.1038/s41467-026-76002-8 (PMC13518939; doi:10.1038/s41467-026-76002-8)
Supplement: Supplementary file 2 [file 41467_2026_76002_MOESM2_ESM.pdf]

nature portfolio

Peer Review File

Sharp Structural Variability of the Gorda Slab Imaged by a Fiber Array

Corresponding Author: Dr James Atterholt

This file contains all reviewer reports in order by version, followed by all author rebuttals in order by version.

Parts of this Peer Review File have been redacted as indicated to remove third-party material

Version 0:

Reviewer comments:

Reviewer #1

(Remarks to the Author)
The manuscript presents an innovative approach to imaging slab structure using DAS recordings of crustal seismicity and autocorrelation-based methods. The authors develop a correlation-stacking strategy to enhance weak converted phases and apply a 3D ray-based inversion to infer slab interface and Moho depths. The study addresses a crucial and challenging topic in subduction zone seismology. I commend the authors for their creative use of DAS and careful handling of difficult-to-observe conversions. The manuscript is well-written but technically overambitious. Several critical methodological and interpretational issues need to be addressed to enhance the robustness and clarity of the work.

Major comments:
1. Justification of ray-based inversion strategy: The authors apply a pseudo-bending raytracing method combined with a grid search to determine likely conversion points for Ps and sP phases. While this is an efficient and commonly used technique, the method assumes conversion occur at travel-time-minimizing points, which may not reflect the distributed nature of scattered energy or the complexity of curved interfaces. I recommend clarifying how interface curvature and dipping slab geometries might influence this assumption and whether the authors consider finite-frequency or waveform-based alternatives.
2. Velocity-depth trade-offs in differential phase inversion: The inversion for slab interface and Moho depths relies on travel-time differences between direct and converted phases. This strategy removes some source side uncertainty but still suffers from a velocity-depth trade-off, especially when crustal and slab velocity are uncertain and very laterally. If the velocity model in inaccurate, the conversion point prediction can be off, even if timing fits are good. The manuscript would benefit from sensitivity tests or uncertainty bounds showing how slab depth estimates shift with reasonable variations in assumed Vp and Vs, particularly above the conversion interface.
3. Identification of converted phases: A key methodological step involves interpreting peaks in correlograms as slab interface or Moho conversions. Given the complexity of scattered wavefield in DAS and the potential for interference from crustal multiples or site-specific scattering, I recommend more discussion (or synthetic validation) of phase identification reliability. How do the authors distinguish between slab-top Ps, slab-Moho Pms and crustal reverberations in their stacked results?
4. Synthetic modelling strategy: The authors use four simplified 1D models to test how different velocity structures affect converted phase time. These models are insightful and highlight the importance of Vp/Vs contrasts. However, I suggest including observational support for these gradients. Also, could any of the models represent a locked interface or shear-zone structure, how would this appear in the waveform?
5. Stacking pseudo-autocorrelograms: The manual picking of peaks may introduce potential bias. Also, the scattered wavefield can change with source radiation, takeoff angles, and paths. This introduces uncertainty in stacked interpretations unless source effects are modeled or corrected for.

Minor comments:
1. It would be helpful to include a schematic summarizing how the inversion functional space was constructed and searched. Elaborate on the inversion method and discuss its potential uncertainties and limitations.
2. Since DAS records horizontal strain or strain rate along the fiber axis, the data represent a single-component measurement. Please discuss how this directional limitation might affect the autocorrelation results compared to data from traditional triaxial seismometers. In particular, how might this influence the detectability, polarity, or amplitude of the converted phases and scattered wavefields used in your analysis?

Specific comments:
1. P3 L102-103, “Picking can be even more … dominated by scattered surface waves …”: Are you referring to earthquake-generated surface waves or those from ambient noise? Why surface waves rather than scattered body waves?
2. P5 L162-163, “… because this conversion produces no observable energy in the horizontal nodal data …”: Why doesn't this conversion produce energy in the horizontal component? Did you compare the horizontal component projected along the direction of the cable?
3. Figure 1(a): The legend for the solid squares is missing from both the figure and the caption.
4. P7 L245-246, “… they have much wider sensitivity to …”: what does ‘wider sensitivity’ mean? why?
5. P8 L276-277, “The first assumption is … at similar depths.” What about anisotropy?
6. P11 L414, “we take a 2 s window”: Why is the template window selected around the first arrival rather than starting after it? Does this mean the window includes the direct P-wave arrival itself? What is the rationale for using a 2-s window, and how sensitive are the results to the choice of window length?

Reviewer #2

(Remarks to the Author)
I read 'Sharp Structural Variability of the Gorda Slab Imaged by a Fiber Array' by James Atterhold and co-authors with great interest. The authors use local earthquake waveforms recorded at a fibreoptic cable deployed in Northern California to derive various structural parameters of the subducting slab crust at a smaller spatial scale than was previously possible. They first enhance signal-to-noise ratio of secondary phases using the pseudeo-autocorrelogram technique, then perform manual phase association and arrival time picking guided by sophisticated forward travel time modeling, and ultimately model the subsurface slab structure first in terms of slab surface depth and then in terms of spatial variability of the slab surface depth.

The work leaves me with fascination and a renewed sense of the great capabilities that fiberoptic sensing facilitates. I think it is valuable contribution to Nature Communications in the sense that it implements a variety of valid and correctly applied specialized processing techniques to the DAS data and gives impulses along various tracks of research that can build on top of this manuscript.

The work lacks some more detailed discussion of previous work, especially regarding the nature of the here imaged plate interface. Processing of some additional seismograms for the small-scale interface structure (Figs. 5 and S2) would greatly benefit the manuscript and illustrate the applicability of the method better. I know that this may be a lot to ask, but to my impression the demonstration of the capability of the method to deliver consistent and reliable results hinges on the comparison of the image of the (about) same structure using various, independent earthquake waveforms.

Finally, minor but important specifications regarding their phase nomenclature and spatial smoothing are required before publication.

* Important clarifications and specifications:

I find the phase nomenclature slightly confusing: A lowercase 's' appears to denote a direct, an uppercase 'S' a converted S-wave. No such distinction is made for 'P', where uppercase is used irrespective of conversion histroy. Perhaps the authors want to denote the direct P-phase with a lower-case 'p' as well? Moreover, the Moho conversion is denoted with a subscript 'M', but no such letter is given for the top of crust conversion. Perhaps a subscript 'T' (top) or 'C' (crust) would be appropriate?

In line 475 the authors explain how they arrive at the spatial smoothing parameter, yet the smoothing that is actually applied is not given. As this parameter is very important to interpret Figures 5 and S2, I would like to ask the authors to provide some numbers regarding which spatial smoothing was actually applied.

The notion of subducted sediments has been brought forward long ago, most recently e.g. by Han et al. (2016), Carbotte et al. (2023), Bloch et al. (2023). I think it would be important to introduce this concept of a more complex subduction interface already in the introduction of the article. Some formulations in the introduction are too vague (see general comments, below), and I think it would be important to introduce this issue early on, as it comes up in the discussion and can help to streamline this part. The discussion regarding the qualitatively different 'smooth' and 'rough' bathymetry might also be of relevance (e.g. Han et al. (2016), Carbotte et al. (2023))

In a related issue, the authors already compare their larger-scale slab model with other studies in Fig. 3b. Yet, I think there are more comparisons that are possible and perhaps meaningful. Carbotte et al. (2023) imaged similar horst-and-graben structure similar to the one that the authors are imaging in their closest profiles PD19 and TD1918. It would be interesting to see if the lateral and vertical dimensions agree, by also plotting their data in figure 5 / S2. The picked bathymetry is openly available (Carbotte et al, 2024). Bloch et al. (2023) provide Receiver Function station measurements for the two nearby stations BK.ARC and BK.JCC. How does the depth of the two provided interfaces (b and t) compare to the depth of the found interface? Can we learn something from this comparison about the nature of the here imaged interface?

The authors process fine-grained slab structures using secondary arrivals from two earthquakes. I personally find Figure S2 much more appealing than figure 5d, as I think it delivers a good sense of the capabilities of the method, comparing

independent data. For how many more earthquakes are the authors able to compute a short-wavelength slab structural image? What is the 'worst' data quality that can still be processed? These are important tests for the more wide-spread application that the authors are suggesting in the discussion section. Also a comparison to data from Carbotte's (2023) profile PD19 and TD1918 data would be interesting and meaningful (see above).

* Additional issues worth considering:

1. A-priori slab structure

The authors assert in various places that the Slab2 model is too deep in the area, yet they plot it prominently in most of their figures. I understand that it is important to be consistent with the processing (e.g. in Fig. 5d it is mandatory to reference Slab2, as this is the parameter they inverted for). Yet, to guide future studies, would the authors suggest to use another, better suited background model?

2. Structural imaging of the Moho

The authors note in various places that they are also able to identify the Moho conversion. Is it possible to extend their analysis of the small-scale structures also to the Moho interface? I understand that this is a lot to ask, and likely goes beyond the scope of this study. Still, it would be good to know how far the authors have come and what would be needed to also construct a Moho model. One could use the Moho-model by Bloch et al. (2023) or Beaudoin et al. (1994) as a starting point to compute synthetic PmS and smP (old phase nomenclature, please revise) arrivals. I would expect to see a much smoother image than in Fig. 5d.

* General comments:

l. 44-46. This last sentence of the paragraph is a bit unspecific to me. If space permits, can the authors give examples of what 'megathrust earthquake phenomenology' they are hoping to be able forecast through their findings?

l. 65 'enables meter-scale coverage over large distances': I think it would be important to be more specific regarding the state of the art realized in this paper. Over how 'large' distances are the authors able to achieve a meter-scale coverage? I think it is about 10km, judging from Fig. 5.

l. 75 'Arcata, CA and Eureka, CA.' I think it would be good for outside-US readers to write out the 'California' abbreviations at least once.

l. 117: 'We can thus use the first arrivals of many earthquakes as template': Are the many template earthquakes not intraslab-earthquakes? It would be good to specify 'many' as 60, as suggested in the caption of Figure S1.

l. 133: 'One' -> Once

l. 228: 'evidence that the slab near the array is too deep': it is the slab model (ref. 28) that is too deep, not the slab.

l. 242: Other explanations would be the addition of subducted sediments, as the authors asserted previously, or underplating of slab material (Sammis & Bostock, 2021; Bloch et al. 2023)

l. 257: 'this bump is not present due to shallow structural effects' -> the bump is present, so delete 'present': 'this bump is not due to...'

l. 312: 'It also suggests' -> Reference of 'it' is unclear. Please write out.

l. 390: '100 km spatial window' -> Do the authors mean within 100km distance?

l. 418 'can be easily automated' -> I assume devil's in the detail here, and so would omit the 'easily'

l. 433: 'the pseudo ray bending method employed in TomoDD'. I would ask the authors to give a reference to the code used. Is it the same ray tracer used by 'simul' family (Eberhart-Phillips et al, 2024)?

l. 461 - 465: The last two sentences are abbreviated beyond understanding. I would like to ask the authors to be a bit more verbose in their explanations here.

* Figure comments:

Fig. 1c) It would be good to plot the predicted 'sP' and 'PS' arrivals in as dotted lines, so the reader has a better chance to judge the data quality themselves.

Fig. 3a) The lines for sP and smP are indiscernible in the correlograms. I suggest using different colors / shades for each phase. The dotted, semi-transparent lines are fine, though. What is the solid black line in the correlograms? In the legend, I only see two black lines (PS and PmS), but on the correlograms I see three or even four. I would ask the authors to make the phase association clearer in the figures. It might be a good idea to place the labels directly next to the phases lines.

3b) I overlooked the legend at first sight. I think it would be important to place it in the frist subfigure as well and adapt the correct and complete phase nomenclature (pmS, SmP, and so on). The figure is missing a y-axis label.

Fig. 4b) The abbreviated velocity model titles are misleading. I suggest to refrain from using the ambiguous term 'crust' (it is unclear whether this refers to the forearc or slab crust), but use 'forearc' and 'slab' or 'oceanic' and 'continental' instead. Also, please provide vertical grid lines as to make the comparison of velocity models easier.

* References

Eberhart-Phillips et al. (2024) Simul2023: a flexible program for inversion of earthquake data for 3-D velocity and hypocenters or 3-D Q doi.org/10.5281/zenodo.5746046

Sammis & Bostock (2021) A Granular Jamming Model for Low-Frequency Earthquakes https://doi.org/10.1029/2021JB021963

Carbotte et al., (2024), Depth to top of subducting Juan de Fuca/Gorda/Explorer plate crust and plate boundary fault within the offshore Cascadia seismogenic zone, 42°N to 50°N. Marine Geoscience Data System (MGDS). doi:10.60521/331666

Reviewer #3

(Remarks to the Author)
This review is for manuscript by "Sharp Structural Variability of the Gorda Slab Imaged by a Fiber Array" by Atterholt et al. The study analyzes DAS seismic data recorded by a 15 km segment of fiber optic cable originally deployed for telecommunication. It analyzes seismic records of 11 intraslab earthquakes in Cascadia subduction zone off the coast of Northern California. It identifies converted waves (PS, sP) from the slab interface and slab Moho. The technique used to improve visibility of these converted phases is a slightly modified version of a technique the authors have previously published about a different seismic phase. The dense spatial sampling of DAS data over a 15 km length allows to map the differential travel times (between converted phases and direct P wave) to continuous changes in topography on the slab interface which maybe useful to further study the role of roughness of slab interface in subduction zones in nucleation of megathrust earthquakes. The study also makes interpretations on amplitudes of the converted waves by comparing with synthetics calculated with different 1D velocity models that may reflect different state of the forearc crust and slab near the interface.

I recommend major revisions. The objectives and the results are noteworthy and significant, though some results (based on differential times) feel more reliable than others (based on amplitudes which may depend on lot more factors like attenuation and 3D structure) but I have nothing concrete against anything. The method is similar to the method authors have previously published (Atterholt and Zhan) but the observations and findings are new and different in context and study area (subduction zone vs southern CA).
The study is kind of vague and silent on many technical details which kind of make me slightly doubt the reliability of results.

Major comments -

- The inversion part is the most confusing and vague part of the study.
L467-472: I didn't understand what functional space means. My interpretation is that they have calculated differential times for 3 different slab depths and are simply linearly interpolating the observed differential time to a slab depth measure.
L473-476: There is some smoothing applied to the results along the array but it doesn't say what the wavelength of the smoothing is. Figure 5d: Some uncertainty (shading) is shown around the results but I didn't find anywhere how it was calculated.
Figure 3b, L211-212: I didn't understand how measurements from individual channels are turned into these distributions. There is no y-axis on the plots. What does "slab parameters" mean here? For the other studies that are cited in Figure 3b, how did you get the ranges? Did you extract the slab depth at your conversion points from the models in these studies and are plotting the range?

- Figure 4c: The comparisons of waveforms is not really fair as the DAS waveform is actually a pseduo-autocorrelogram whereas the synthetics are strain synthetics. For apples-to-apples comparison, you should correlate the P-wave with the entire waveform for the synthetics and show that.
In this figure, the DAS waveform is aligned with the peak of P-wave packet at t=0 but the synthetics are aligned with the first onset at t=0. This is inconsistent and may make the reader doubt the reliability of the results.
L637-638 Please show the actual waveform you are using to make the measurements instead of a 1 km average. Otherwise it paints a false picture of quality of measurements
L169: You are drawing conclusions about the M4.8 event here using waveforms for M5.3 event. Given you have shown that composition of the wavefield could depend on source-receiver distance (Figure 4d) and those two events are at different distances, maybe you should also show similar synthetics for the M4.8 event in the electronic supplement. I don't think the

results will change but you should do it to dispel all doubts.
L172: "for most of our observations", L190-191: The synthetics shown are for an event that is an exception. You should also show synthetics for an earthquake that represents most of your observations in an electronic supplement.
For the nodal seismometer waveforms, all three components must be shown.

- If possible and considered useful by authors, I would like to have a short discussion added on the consequences of ignoring finite-frequency effects. Even though you are saying your observations are high-frequency (> 6 Hz) and wavelength is <750 m (L302), this interpretation is probably meaningful for direct body waves. Your measurements are correlations (P waves and remaining wavefield) and stacks of correlations across different earthquakes. Correlations are known to result in sidelobes instead of a simple wave packet if the assumptions are not satisfied making it difficult to precisely pick an onset. The coda not only contains contribution of heterogeneities at the receiver position, but also at the source position that may be different for earthquake pairs with different locations and radiation patterns. Some phase conversions are weak (for example, 73947930, 74001081, 73961291), some show other phases diverging off (73827571). The saturated colors make it little bit challenging to judge how sharp the peaks of converted phases are in the correlograms. If an onset is too emergent, you can follow a peak or trough and you can measure the relative variation of differential traveltimes of the converted phases because they are coherent from channel to channel but then the interpretation is from a wave packet that is fraction of a second width (> 0.2 s) even though your travel time variations are < 0.1 s.

- All waveforms and record sections look somewhat bandlimited to me in the figures. Even though it is mentioned many times that you use high-frequency measurements (> 6 Hz) (L295,L302), I didn't find any mention of a bandpass filter anywhere (my apologies if I have missed it). Please write if you are applying any filter.

- A lot of quality-related terms have been used (L214: precision, L215: uncertainty, L243: very-high quality, L391: signal-to-noise ratio) without any quantitative definition anywhere.

Minor comments -

- For discussion of relative amplitudes (L149-204) from synthetics, I didn't find any info on the density and attenuation structure assumed (my apologies if I missed). L457: FK integration requires layered structure I think, write what is the thickness of layers used to discretize the models in Fig 3b. Write source duration.
- L82: Gorda slab off the Northern California coast
- L93: similar source mechanism and similar depths
- L103: surface waves scattered off the direct arrival near the surface, rather than the direct arrival
this is just to indicate this is not the main surface wave getting scattered
- L110: earthquake wavefield -> P-wave wavefield ?
- L126: number not reported in other studies -> add reference here
- L176: little bit counter-intuitive to me why marine sediments and finite fault thickness would make it faster. maybe expand/explain in a sentence.
- L226: cite Fig S4 here
- L238: cite Fig 3b here
- L331: write how much is the Vp/Vs ratio in the model in the top 5 km below the array so people have a reference on how good or bad it is
- L389: 100 km spatial window is a bit vague,
- L394: write the name of the catalog. Cite Table S1 here.
- L414: make it clear whether this is observed pick or pick predicted by the velocity model
- L424-425: this can be dangerous, if two events are too far apart or have different mechanisms, different phases can have different polarities (like, P waves have same polarity but converted waves may have different polarity)
Figure 1a. What are the black lines? How do you convert the conversion points to the squares? Add degree symbol to geographic coordinates
Figure 1c. Write if t=0 is origin time
L611, L616 scattered wavefields -> P wave window
Figure 4b. This plot maybe more informative if it has two subplots with one showing all Vp models plotted on top of each other and one showing all Vp/Vs models. Move the Vs to electronic supplement as your primary parameters for constructing the models are Vp and Vp/Vs. Minor grid so people can judge values.
Figure 4c. The right two dotted lines are for ? The subscripts in names of converted phases are not explained - wz, conv, refl
L645 lateral sensitivities -> conversion points
Figure 5d write what is the range of differential travel times in the text for producing this range of perturbations of slab interface depth

Figure S1b plot both positive or negative difference rather than absolute value
Figure S1c maybe difference in backazimuth from array to the two earthquakes is more meaningful?
Figure S4 I am not sure how the quantity in colobar (depth) is different from event depth and slab depth (two axes) ?
Figure S5 Which 1D model was assumed? What was the source depth?
Figure S6 source-receiver distance and source depth?

Version 1:

Reviewer comments:

Reviewer #2

(Remarks to the Author)
This is my second review of 'Sharp Structural Variability of the Gorda Slab Imaged by a Fiber Array' by James Atterhold and co-authors.

The manuscript was challenged by three reviewers, where the comments of the other two reviewers pertained mostly to methodological aspects while my own review focused on the interpretation. From reading the rebuttal letter, I feel the authors have undertaken an apropiate effort to resolve all reviewer's concerns. The manuscript has methodological emphasis, with a clear motivation and very well demonstrated application.

As the other reviewers noted, the method is often heuristic (time window selection, peak detection, selection of high-quality observations). For this reason, I would argue it is warranted to evaluate the quality of the results in a similarly heuristic manner and would like to ask the authors to exhibit more than only the best results, that is show the data of figure R2, at least in the supplement, if not in the main text.

Finally, some parts of the manuscript still have an overly colloquial tone that sometimes makes it difficult to understand the full meaning of what is written. Examples of this are given below.

After the correction of these minor imprecisions I would like to recommend the manuscript for publication in Nature Communication.

Examples of inaccurate language:

l. 58 phase conversions off the slab interface -> I think 'off' should this rather be 'of'? Please double check

l. 59 are *of* high-frequency

l. 70 'increasingly ubiquitous' is a tautology

l. 153 its conversion is *of* very high amplitude

l. 222 search this interpolant for the slab parameter depth -> lookup interface depth?

l. 259 a combination of these things -> of these processes

l. 454 We chose 10 km because we wanted to ensure -> We chose 10 km to ensure

l. 498 omit 'for this'

l. 507-508 This sentence should be expressed in propper language: 'The grid points that minimize the P to grid + S from grid and the S to grid + P from grid travel times yield approximate Ps and Sp converted phase travel times.'

perhaps as:

The grid points that minimize the travel time of (a) the P-wave to grid plus the S-wave from the grid and (b) the S-wave to grid plus the P-wave from the grid travel times yield approximate Ps and Sp converted phase travel times.

In a similar manner ll. 517 - 523 may be better expressed as a propper list.

Reference 2 is ill formated

Reference 16 was unavailable at the time of review.

Ref 17 is missing a Journal

Reviewer #3

(Remarks to the Author)
This is my 2nd review of the study "Sharp Structural Variability of the Gorda Slab Imaged by a Fiber Array" by Atterholt et al. This revised paper is significantly improved. The authors have worked hard to address the reviewers' comments and concerns. I recommend only minor revisions (typos and minor clarifications) after which the paper should be accepted. I don't need to look at this paper again.

MINOR COMMENTS

- L166-169: the roman numerals should come before the description of the model for consistency with elsewhere in the paper
- L179: faster forearc crust?

- L279: by two events on different channels
can you write the specific range of channels/along-fiber distance for these two events here in parentheses.
- L328: geology -> geology and seismic velocities
- L408: an -> and
- L517-525: similar to how you changed the abbreviated titles of the models in Fig 4b in response to reviewer 2's comments, I recommend similar changes here as well to clarify if the crust is forearc or slab crust. For example, slow crust -> slow forearc crust, etc.
- L553: interface -> interface and a frequency of ~xx Hz.
- Figure 3 caption, L724-726: Green/black picks/dotted lines, I think this was for the previous version of the figure. The caption should be corrected.
- Figure 3 caption, L725: 3D raytracing -> 3D raytracing and the reference interface model.
You should make it clear whether you are using the reference interface model or model incorporating your new corrections for raytracing, otherwise readers will be confused.
Same for Figure 5c.
- L739: S8 -> S9
- L742: 12-13 km along-fiber distance

ELECTRONIC SUPPLEMENT

- L39: line -> polygon
- Fig S5: The phase symbols sP, PS in both x-axis and y-axis need to be corrected to be consistent with elsewhere in the paper
- Fig S5 caption, L65: equivalent -> numerically equal ?
- Fig S5 caption, L67: magnitude -> amplitude or size
- Fig S6 caption, L77, L79: inaccuracy -> it would be good to change it to underestimation or overestimation (based on the numbers) to be clearer
- Fig S6 caption, L79: cersus -> versus
- Table S1: Mark the 11 events actually used for the phase conversions with a star.

Reviewer #4

(Remarks to the Author)
This manuscript by Atterholt & coauthors presents an innovative application of DAS to detect slab-converted phases and infer slab structures. The quasi-autocorrelogram stacking technique is interesting and the study area is relevant. However, I'm not 100% convinced this manuscript is a contribution at the Nature Communications level, as I believe more observations in different contexts, maybe with longer cables might be needed to define conclusive these findings. Nevertheless, as far as I know, this is the first tentative imaging of slabs with converted phases recorded by DAS, thus impressive, if one thinks about the possibility that opens for monitoring oceanic slabs with typical offshore cables (perpendicular to the trench) and onland cables (parallel).

Major Comments
1. Observations might be inconclusive for the claims
The primary results rest on converted phase observations from 11 events, however with high quality imaging tentative from effectively 2 events (nc75070761 and nc73947920; Figures 5 and S2). The correspondence between these two estimations of the slab topography bump is encouraging. As it stands, the paper demonstrates the method has the potential to image slab structures, but I would suggest relaxing the conclusions: this method must be demonstrated in other contexts. As far as I know, there are other oceanic DAS cables above subduction zones. There might be a lack of converted phases in these contexts, but the authors might mention this possibility. If demonstrated in another context this would be really a major scientific contribution!
2. Circularity in the spatial interpretation of conversion points for raytracing
The conversion points are computed by raytracing to the Slab2 interface model. However, one of the results is that Slab2 is too deep by 1 -3 km (fig 1 c). I might have misinterpreted this result, but it's a sort of circular problem, isn't it? I had some difficulties in trusting the described topographic slab bump, which is smaller (or similar) in dimension to this mismatch. If the reference model depth is found not correct, then the incidence angle is supposedly incorrect and as a consequence also the conversion podsint. Is there a way to estimate the slab geometry error or iterate the inversion to address this?
3. DAS-specific variability in amplitude and polarity underestimated
The authors interpret variations in converted phase amplitude and polarity as evidence for fluid distribution in the slab. From my understanding, when working with DAS, especially telecom cables, lateral variations in amplitude and coherence are complex to model, either source related, but also installation dependent (e.g., near surface effects). Thus, the amplitude and polarity based interpretations (Figure 4) remain vulnerable to the azimuthal sensitivity issue (did authors take into consideration this?). An analysis of fiber azimuth variations and their expected effect on the DAS correlogram polarity for each event might be useful.

4. Manuscript structure
The Results section is largely interpretation of the results, thus subjective. In my understanding, resuylts must be objective, then the discussion should built on the results the subjective descriptions, thus supporting conclusions. That's what readers expect. The Discussion section is too long, making the paper a bit hard to follow, especially at a first read. Maybe using more subchapters might help.
In practice, results should present observations and measurements (correlograms, picks, and inverted slab depths), while

the Discussion should contain physical interpretation, comparison with other studies, limitations, and implications.

Minor comments
Figure 5 panel c: The converted phase lines are difficult to see against the correlogram background. Please increase the line weight or use a higher-contrast color scheme for these lines
DAS installation: Data should include a more detailed description of the fiber geometry.
line 195: ..

Version 2:

Reviewer comments:

Reviewer #4

(Remarks to the Author)
This is my second review of the manuscript.

I thank the authors for their responses to my previous comments, which have clearly improved the manuscript. Regarding point 3, however, after reading again both the response and the revised manuscript, I remain somewhat hesitant.

Main issue: kilometer-scale interface roughness and its implications in the overall picture of the manuscript
I still cannot accept the manuscript in this version because the implications regarding kilometer-scale interface roughness are not well supported by the data. I am satisfied with the observations of converted phases on autocorrelograms, which clearly demonstrate the advantage of having such a high-resolution tool as DAS. However, I am not convinced by the interpretation based on only one, or at most two, events of the relative changes in depth of the slab along the profile (the slab bump). The authors should exclude the possible effect of near-surface variations in the velocity structure, which could potentially explain differences of 0.01–0.05 seconds in the observed-minus-expected times. Additionally, it's unclear to me how these phases were picked…. by hand?
I noted that the signal anomalies being interpreted fall within the tens-of-milliseconds range (Figure S5), how do they relate to picking uncertainty? Furthermore, the potential effect of coupling variability along the telecom cable has not been discussed. What do authors think about the possibility that spatially-irregular cable coupling can generate anomalies in the correlograms that mimic deeper structural variations?
As you might understand, my concern is that I would like to exclude the near surface effects in potentially providing similar time differences to deeper (at the slab interface) lateral topographic anomalies. Why? Because being capable of image such slab topography at such fine scale has large potential implications for subduction zone and earthquake rupture models. Either authors relax their claims on this or support it with more data excluding near surface effects.
A possible suggestion to the authors to exclude influence of the near surface: Do you have DAS recordings of shallower events, not intraslab? Do you see lateral variations in the frequency content of such signals (e.g., power spectral densities) or systematic delays in arrival times of direct phases in such events? If you see similar trends (not absolute values of course, but similar relative shapes) to what you observe on predicted-observed times this might be an indication of the influence of near-surface. I would say that also with different shapes but similar relative changes in the traveltimes it would be alarming for the claims on the slab origin of lateral variations in delay times compared to the expected times. If instead the shapes are different and the influence of the near surface is smaller, these results might support the claims on the deeper origin of the anomalies.
I would also recommend providing zoomed time windows around the picks in Figure 3, which is a key for better understanding the spatial coherence on the converted phases and the uncertainties in the picks.
Some examples from the text:
Regarding lines 271–276, the authors argue that the similar bump observed in two profiles recorded on different fiber segments excludes a near-surface origin. This reasoningt needs to be explained more clearly: why is it self-evident that two events recorded on partially overlapping fiber segments rule out a near-surface structural effect?
More broadly, the manuscript is inconsistent in how it presents the robustness of the slab topography interpretation. In some sections the roughness finding is addressed as uncertain (e.g., lines 392-), while in others it reads as a well-established result, for example:
Lines 291–293 state: "This inversion makes clear that fiber arrays can be used to image sub-kilometer megathrust topography at deeper depths than are accessible with standard techniques."
And also in the abstract (lines 19–21): "...which reveals kilometer-scale interface roughness."
The terms “makes clear” and “reveals” are typically assured facts, not uncertain.

Version 3:

Reviewer comments:

Reviewer #4

(Remarks to the Author)
Review #2 Atterholt Nature comm. 2026

Atterholt and coauthors provided a very detailed response to my concerns. I am satisfied with the answers. I suggest reporting these additional pieces of evidence (e.g., the figures with the near surface velocity), if possible, in the supplementary material of the final version of the manuscript. This imo would strengthen the support for the results discussed in the manuscript.
I also appreciate the more cautious tone of the revised manuscript, which now reflects that the results suggest, rather than clearly demonstrate, that converted phases, using the proposed methodology and within the analyzed context, can image heterogeneities in the slab topography. This is important, as in my opinion, there is space for other studies in other contexts, and with a longer cable, to further confirm this.

Further minor revisions (no need to send the manuscript back to me but I'd be happy if authors try to implement them):
Line 271:
"In particular, we resolve a bump that is 300 m high and 3 km wide." →
"In particular, the results point to the possible presence of a bump approximately 300 m high and 3 km wide."
These values carry uncertainties, so they should not be stated without clarifying that they are estimates.
Figure 1a-b
I would suggest highlighting the conversion points and their epicentral location to better understand the square points in a) and the corresponding model in b). In practice, make crystal clear that those square points are indeed the "epicenters" of the conversion point at depth.
Figure 1c-d
Please make sure that from the figure itself it is clear that those lines are synthetic traveltimes. I know this is already stated in the caption, but with the new AI pickers for DAS data sometimes it's not immediately clear the difference between picked phases and synthetic traveltimes.
Figure 4b
The color for the caption Vp and Vs are almost invisible. It might help using bold characters to highlight the color.

Open Access This Peer Review File is licensed under a Creative Commons Attribution 4.0 International License, which permits use, sharing, adaptation, distribution and reproduction in any medium or format, as long as you give appropriate credit to the original author(s) and the source, provide a link to the Creative Commons license, and indicate if changes were made.
In cases where reviewers are anonymous, credit should be given to 'Anonymous Referee' and the source.
The images or other third party material in this Peer Review File are included in the article's Creative Commons license, unless indicated otherwise in a credit line to the material. If material is not included in the article's Creative Commons license and your intended use is not permitted by statutory regulation or exceeds the permitted use, you will need to obtain permission directly from the copyright holder.
To view a copy of this license, visit https://creativecommons.org/licenses/by/4.0/

REVIEWER COMMENTS

Note: All line numbers reference the tracked changes documents.

Reviewer #1 (Remarks to the Author):

The manuscript presents an innovative approach to imaging slab structure using DAS recordings of crustal seismicity and autocorrelation-based methods. The authors develop a correlation-stacking strategy to enhance weak converted phases and apply a 3D ray-based inversion to infer slab interface and Moho depths. The study addresses a crucial and challenging topic in subduction zone seismology. I commend the authors for their creative use of DAS and careful handling of difficult-to-observe conversions. The manuscript is well-written but technically overambitious. Several critical methodological and interpretational issues need to be addressed to enhance the robustness and clarity of the work.

We thank you for taking the time to review our manuscript and providing these thoughtful comments. We have tried to address each point directly and in doing so have made several modifications to the manuscript. We feel the manuscript is stronger as a result.

Major comments:

1. Justification of ray-based inversion strategy: The authors apply a pseudo-bending raytracing method combined with a grid search to determine likely conversion points for Ps and sP phases. While this is an efficient and commonly used technique, the method assumes conversion occur at travel-time-minimizing points, which may not reflect the distributed nature of scattered energy or the complexity of curved interfaces. I recommend clarifying how interface curvature and dipping slab geometries might influence this assumption and whether the authors consider finite-frequency or waveform-based alternatives.

Thank you for this important comment. We agree that our approach is a simplification of finite-frequency behavior, especially in the presence of complex structure. In this study we compute high-frequency Ps and Sp paths using Fermat rays (computed via a grid search over pseudo-bending solutions), and we acknowledge finite bandwidth by smoothing the recovered interface from the subsequent inversions with a kernel whose scale is set by the local wavelength of the converted phase. This is a pragmatic simplification of "fat-ray" effects that account for wavelength-scale averaging of structure in a neighborhood of the geometric path (e.g., wavelength smoothing in Lomax, 1994). As is shown in wave-equation sensitivity work (e.g., Sava & Biondi, 2004), this type of method can become inaccurate, especially when the interface topography or curvature is on the same spatial scale as the phase wavelength. The reason for this breakdown is that wavepaths are frequency dependent (Biondi, 2006), and the resultant sensitivity kernel is the combination of different focusing and defocusing effects from wavelengths that are sensitive to distinct structures. So, when lateral variations approach the converted-phase wavelength, sensitivity can be shifted or distributed away from the geometric path. We therefore restrict interpretation of interface topography or curvature to scales greater than the wavelength and treat our smoothing as a conservative approximation rather than a substitute for full finite-frequency kernels. In general, the overall dip of the slab is very shallow at the wavelengths used in this study. Finite-frequency kernels for Ps or Sp in 3D at the

frequencies used here would be preferable but are computationally prohibitive and we consider overcoming this computational limitation beyond the scope of this work. To address your suggestion, we 1. made our methodology and smoothing choice explicit, and 2. added a discussion of the limitations and applicability of our ray-based approximation. We have added the following text to the discussion:

"Additionally, the time-minimization geometric raytracing that we use is an approximation that does not include finite-frequency effects. To partially account for the finite bandwidth of the wavefield, we smooth the inverted interface by the wavelength of the converted phase in the neighborhood of the conversion. But this approach, which is a pragmatic simplification of commonly used methods in tomography[35], can be inaccurate in the presence of strong structural heterogeneity. In some cases, structural complexity sensed over a finite bandwidth can shift the distribution of sensitivity away from the time-minimization path. This problem is especially acute when the interface topography varies at scales similar to the converted phase wavelength of 750 m[36]. For this reason, we are careful to only interpret structures that are several multiples larger than this scale. Using Sp and Ps finite-frequency kernels would produce a more accurate result[37]. Frechet kernels for Sp an Ps amplitudes typically have high and uniform amplitude over scales of a few km[38]. The computational expense of computing these kernels at the frequencies used in this study is large and we do not yet have a high-resolution starting velocity model to work with. We thus save the implementation of that approach for future work."

Where: Lines 401-415

2. Velocity-depth trade-offs in differential phase inversion: The inversion for slab interface and Moho depths relies on travel-time differences between direct and converted phases. This strategy removes some source side uncertainty but still suffers from a velocity-depth trade-off, especially when crustal and slab velocity are uncertain and very laterally. If the velocity model in inaccurate, the conversion point prediction can be off, even if timing fits are good. The manuscript would benefit from sensitivity tests or uncertainty bounds showing how slab depth estimates shift with reasonable variations in assumed Vp and Vs, particularly above the conversion interface.

Thank you for this comment. In the initial submission, we performed a couple sets of sensitivity tests with this tradeoff in mind. These results are summarized in Figure S5. These tests modified Vp/Vs (which produces the strongest tradeoffs for the phase differential times) in the shallow crust. We chose the shallow crust for these tests because that's the location of the velocity model that is least constrained, but the implications are generalizable to velocity uncertainty throughout the forearc:

"Relatedly, P-Ps differential times are highly sensitive to the uncertainties in the Vp/Vs ratio in the forearc crust. We perform our inversion using 3D raytracing using a velocity model with a well resolved Vp/Vs ratio, but this model has poor sensitivity in the shallowest ~5 km22. The Vp/Vs ratio and the slab depth trade off with each other, but very large perturbations to the Vp/Vs ratio are necessary to account for the differences in slab depth we resolve with our inversion. For example, we resolve a large change in the absolute slab depth in Figure 5 (~1.3 km). To get this change in the absolute slab depth, we would need the Vp/Vs ratio to be

inaccurate by ~10% throughout the entirety of the shallowest 5 km of the crust (Figure S5). The roughness measured on the megathrust fault in Figure 5 is of lower amplitude than the absolute change in slab depth (~300 m). To produce this peak, there would need to be a sharp-gradient inaccuracy in the relative Vp/Vs ratio along the array. This inaccuracy would likely need to be kilometers deep and narrow (Figure S5). This is conceivable, but given that our observations can be verified across events and at different channels (Figure S2), we prefer the interpretation that the most prominent features are indeed slab topography. However, these tradeoffs show that when inferring short-wavelength slab topography, which will be important for the next generation of slab models, accurate and precise 3D velocity models with sensitivity to the full crustal section will be fundamentally important."

Where: L364-381

3. Identification of converted phases: A key methodological step involves interpreting peaks in correlograms as slab interface or Moho conversions. Given the complexity of scattered wavefield in DAS and the potential for interference from crustal multiples or site-specific scattering, I recommend more discussion (or synthetic validation) of phase identification reliability. How do the authors distinguish between slab-top Ps, slab-Moho Pms and crustal reverberations in their stacked results?

Thank you for this comment. This is an important point. We dedicated substantial space in the original manuscript to discussing the assumption that these phases are associated with either the slab interface or the slab Moho and describing how we were able to distinguish between Sp and Ps phases in this study:

"In this study, we make clear observations of secondary amplitude peaks and attribute them to conversions related to slab structure. There are some potential sources of nonuniqueness that require assumptions to resolve. The first assumption is that these phases are related to slab structure, rather than other structures at similar depths. This assumption is well-justified. The slab is a first order structural feature that expectedly imposes sharp impedance contrasts at depth. We also have good estimates of the approximate depth of the slab and slab crustal thickness, and 3D raytracing suggest that these conversions all happen within a few kilometers of either the expected slab interface or slab Moho (Figure 3)."

Where: L302-309

"The classification of the sense of conversion, Ps or Sp, is sometimes more challenging. In many cases, the expected arrival times of these two phases are very different, and the classification is easily made by evaluating which expected arrival time more closely matches the data. If the expected arrival times are similar, then the array-side moveout of the observed phase compared to the expected phase arrivals may be employed towards classification. This would be a stronger constraint for longer arrays (ours is ~15 km). If the expected moveout is also similar, then source-receiver distance can also be used to determine whether a phase is unlikely to be Sp, because Sp is expectedly weak at source-receiver distances beyond 60 km (Figure 3). These methods of distinction cover all cases included in this study, though it is possible that in future studies, new methods for distinguishing between phases will be needed. These could include

using a priori knowledge of the geology to anticipate the expected phase polarity or using collocated 3-component measurements to observe the phase polarization (Figure 4)."

Where: L319-331

However, we agree that the specific points you mention should also be discussed, namely distinguishing between Moho and interface phases and addressing crustal multiples. The Moho phases for this dataset are always expected to arrive at much different times than the interface phases, assuming Slab2 is not inaccurate to a large degree (> 7 km). We assume you were talking about multiples in the slab crust, because the differential times for forearc crustal multiples and depth phases (at these hypocenter depths) would expectedly arrive much later than any of the phases used in this study. While we cannot discount the presence of these multiples, we now explicitly state that it is an assumption that we are observing direct mode conversions. This assumption is justified by the expected (though not universal) relative reduction in amplitude of reverberations due to energy partitioning. We have added the following text to the discussion above:

"When we observe a secondary phase off the interface, we assume it is the direct conversion rather than a multiple within the slab crust. This is justified by the expectation that, while not always true, energy partitioning generally causes amplitudes of multiples to be smaller than the direct conversion[32]. We can easily distinguish between slab interface and slab Moho phases, because $S_{M}p$ expectedly arrives much earlier than Sp and $P_{M}S$ expectedly arrives much later than Ps. This requires the assumption that the errors in Slab2 are not very large (lower than the slab crustal thickness); this is reasonable given the model's estimated errors[29]."

Where: L311-318

Site specific scattering effects are inherently removed by the cross-correlation. For this method, the more complicated the near-surface scattering in the DAS data we observe, the better, because this produces clearer peaks in the cross-correlation when that scattering is reproduced by a secondary phase.

4. Synthetic modelling strategy: The authors use four simplified 1D models to test how different velocity structures affect converted phase time. These models are insightful and highlight the importance of Vp/Vs contrasts. However, I suggest including observational support for these gradients. Also, could any of the models represent a locked interface or shear-zone structure, how would this appear in the waveform?

Thank you for this comment. We have modified the initial description of these models to include citations to observations (from Delph et al. (2018), Guo et al. (2021), and Bloch et al. (2023)) whose models inform choices. We also now note that the 1D model is an average of the 3D Model from Guo et al. (2021) and that these models contain Vp and Vp/Vs contrasts that are of the same order, though slightly exaggerated, as contrasts in refraction results and in Vp/Vs tomography (Guo et al. (2021) and Beaudoin et al. (1994)). The modified text is:

"We consider four 1D models (modified from an average of Guo et al. (2021)22) that mimic or vary observations from prior studies and produce a range of observations (see Methods; Figure 4): a model with slow, hydrated forearc crust[22,23] (i), a model with slow forearc crust and hydrated slab crust[22] (ii), a model with fast forearc crust[23] (i.e., a smoother transition from slab to forearc) and hydrated slab crust (iii), and a model with slow, hydrated forearc crust and a weak upper slab crust[7] (iv)."

Where: L163-168

"Though slightly exaggerated, these Vp and Vp/Vs contrasts are of the same order as observed in refraction results and travel-time tomography.[22,26]"

Where: L532-533

Regarding the shear zone, we imply that the weak zone from the observation from the M5.3 is possibly a shear zone:

"This is within the range of expected thicknesses for subducted marine sediments[25,26] or finiteness of the megathrust fault due to shear[4,6], but these layers are generally expected to have lower velocities than the overlying forearc crust[4,27]."

Where: L184-187

However, the M5.3 conversion points are outside the locked zone. Characteristic structure within the locked zone in a 1D model is difficult to determine with our dataset, because the observations within that zone are all expectedly due to a single interface (because there is only one observable peak). We do, however, note our observations within the locked zone are consistent with a hydrated slab crust, as observed elsewhere, and that the interface roughness is also within the locked zone.

"All other observations of Sp have positive relative polarities and all observations of Ps have negative relative polarities. This would suggest that the other observations are more consistent with phase conversions generated by a model of a fast crust and hydrated slab (model iii; Figure 4). The explanation for this difference may be that the Mw5.3 earthquake is located to the east of all other intraslab earthquakes used and outside the high slip deficit patch that encompasses most of the events and phase conversion locations in this study18 (Figure 1). Previous studies have observed a correlation between slab hydration and interface locking22, expectedly because the slab is dehydrated as it transitions into a ductile regime. The results of this study support this observation, and relatedly supports the conclusion that, within the locked zone, the pore fluid pressure in the slab crust is elevated."

Where: L205-215

"The profile shown in Figure 5 has sensitivity to a narrow portion of the locked zone of the Southern Cascadia megathrust (Figure 1). More extensive observations within the locked zone

and comparable observations outside the locked zone are needed to determine if and how structural variability in this region may be connected to megathrust locking."

Where: L287-290

We also note that by leveraging more fiber, we anticipate being able to observe the transition from locked to unlocked more clearly:

"The results shown in Figure 4 suggest a connection between the locked zone and the hydration of the slab, but the analysis is limited by the fact that few observations are outside the locked zone. The connection between slab hydration and megathrust locking could also be further clarified by more extensive and diverse measurements of phase conversion polarity and amplitude. These outstanding problems could be addressed in this region following an expansion of the fiber array used in this study that leverages a larger portion of the Middle Mile Broadband Initiative fiber (Figure S8)[34]."

Where: L422-428

5. Stacking pseudo-autocorrelograms: The manual picking of peaks may introduce potential bias. Also, the scattered wavefield can change with source radiation, takeoff angles, and paths. This introduces uncertainty in stacked interpretations unless source effects are modeled or corrected for.

Thank you for this comment. This was unclear in the description of the methodology, but we manually select a time window around the zero-phase peak and then objectively select the peak using a local maximum (or minimum). This ensures we are not accidentally making picks on peaks that are not zero-phase peaks, but we are still using an objective method for picking that minimizes bias in the alignment. We have added the text to the description of the methodology:

"To achieve this alignment, we visually identify zero-phase peak and manually choose a narrow time window that encompasses this peak. We then pick the zero-phase peak times by selecting a maximum (or minimum) within this window."

Where: L489-491

This source effect is possible, and we account for it both by not using templates that do not produce a strong zero-phase correlation peak (suggesting the scattered wavefields are different), and by correcting for differences in polarity by multiplying templates that produces a negative zero-phase correlation peak by negative 1.

"If the correlation between P-waves is poor, we do not use the template. If this zero-phase time is symmetric around a negative peak, we assume this is due to source radiation differences and multiply the correlogram by negative one."

Where: L494-496

Minor comments:

1. It would be helpful to include a schematic summarizing how the inversion functional space was constructed and searched. Elaborate on the inversion method and discuss its potential uncertainties and limitations.

Thank you for this comment. Firstly, we have changed the language from "functional space" to "linear interpolation function" to be more descriptive and accurate. We dedicate substantial space in the discussion to discuss potential limitations of this inversion method that includes synthetic tests (Figure S7) that show how the inversion fails to reproduce true structure:

"The inversion for slab topography was performed using a linear interpolation function defined by computing phase differential times for several slab interfaces, offset by absolute values. This inversion is an approximation, performed for computational reasons, that fails to account for ray-bending effects caused by short-wavelength interface topography. Ray-bending has a smoothing effect on the differential time measurements. For bumps that cause shallowing of the slab interface, rays are preferentially directed to the bump to minimize the travel time, resulting in a bias in our inversion towards an artificially wider bump. For bumps that cause a relative deepening of the slab interface, rays preferentially avoid the bump, resulting in a bias in our inversion towards an artificially lower amplitude bump. The effect of these biases are mild and examples are shown in Figure S7. With greater computational resources, these biases could be avoided by performing an iterative inversion for the full interface, rather than inverting for slab depth for each channel individually."

Where: L390-401

This discussion is immediately followed by the discussion of the potential pitfalls of not including finite-frequency kernels in the inversion (see above). Since this is ultimately just a linear interpolation function, we think a schematic would be unnecessary.

2. Since DAS records horizontal strain or strain rate along the fiber axis, the data represent a single-component measurement. Please discuss how this directional limitation might affect the autocorrelation results compared to data from traditional triaxial seismometers. In particular, how might this influence the detectability, polarity, or amplitude of the converted phases and scattered wavefields used in your analysis?

Thank you for this comment. Because the scattered surface waves are so strong and pervasive in DAS datasets, and because these surface waves propagate concentrically from the local scatterer, there is usually a component of motion that is polarized in the direction of the fiber. We have added a few sentences to the methods discussing this:

"Moreover, even if the polarization of the incoming converted wave is oriented at an unfavorable angle relative to the cable azimuth, the locally generated scattered energy will likely include some energy that propagates along the cable azimuth and be well recorded."

Where: L473-476

We also note that we discuss the potentially utility of having a 3-component measurement, especially for phase classification in the discussion:

"These methods of distinction cover all cases included in this study, though it is possible that in future studies, new methods for distinguishing between phases will be needed. These could include using a priori knowledge of the geology to anticipate the expected phase polarity or using collocated 3-component measurements to observe the phase polarization (Figure 4)."

Where: L327-331

Specific comments:
1. P3 L102-103, "Picking can be even more … dominated by scattered surface waves …": Are you referring to earthquake-generated surface waves or those from ambient noise? Why surface waves rather than scattered body waves?

Here we are referring to scattered surface waves from the interaction of energy generated by earthquakes with shallow structure. There are likely scattered body waves present, but the primary observable scattered waves in this case are surface waves. This is because strainmeters are especially sensitive to slow apparent velocity phases, and thus slow surface waves propagating at the surface dominate. Previous backprojection results have verified that these scattered waves are surface waves by showing that they are dispersive (Atterholt et al., 2021).

2. P5 L162-163, "… because this conversion produces no observable energy in the horizontal nodal data …": Why doesn't this conversion produce energy in the horizontal component? Did you compare the horizontal component projected along the direction of the cable?

Our interpretation is that this does not produce energy in the horizontal component because it is a near-vertically polarized longitudinal wave (i.e., it's Sp). We did make this comparison, which is now shown in this document in response to another reviewer comment below. There is not energy in both the N component and the E component. We now include both polarities in Figure 4 for a more complete comparison.

3. Figure 1(a): The legend for the solid squares is missing from both the figure and the caption.

We have added a more explicit description of these points to the Figure 1 caption:

"Also plotted as square points are changes to Slab2 resolved in this study (Figure 4)."
And we have added these points to the legend.

4. P7 L245-246, "… they have much wider sensitivity to …": what does 'wider sensitivity' mean? why?

Thank you for this comment. We mean to say that for a single event measured by the array, Ps conversions will cover a larger segment of the slab than Sp conversions. This is because of how travel-minimization works differently for Ps versus Sp conversions: Sp conversions minimize travel time by converting near the source, resulting in all the conversion points clustering above

the hypocenter, whereas Ps conversions minimize travel time by converting near the receiver. Since the receivers are spread out along the array, the conversion points for Ps are spread out as well, mimicking the array geometry. This means Ps conversions cover a larger area. We have modified this sentence for clarity:

"…for an individual event, they are sensitive to longer segments of structure at the slab interface (Figure 5a)."

Where: L266-268

5. P8 L276-277, "The first assumption is … at similar depths." What about anisotropy?

Thank you for bringing this up. You are right, we also assume the effects of anisotropy, which could affect the timing, are minimal:

"We also assume that the effects of anisotropy, which could produce small shifts in Ps arrival time on a single horizontal component, are minimal."

Where: L309-311

6. P11 L414, "we take a 2 s window": Why is the template window selected around the first arrival rather than starting after it? Does this mean the window includes the direct P-wave arrival itself? What is the rationale for using a 2-s window, and how sensitive are the results to the choice of window length?

Thank you for this comment, this gets to the heart of why we use the templates. The window is indeed meant to encompass the first arrival. The first arrival (and the seconds after it) include the scattered surface waves generated by the P-wave. The point of this approach is to reproduce the results of a straightforward autocorrelation (i.e., autocorrelated an event's P-wave scattered energy with the scattered energy from a converted wave) with a different noise distribution. Thereby, stacking reduces the noise. We also need to reproduce the zero-phase peak for the timing correction of the quasi-autocorrelogram.

The 2-second window is meant to be long-enough to capture the scattered surface waves from the first arrival, while remaining targeted, to the degree possible, to only include the first arrival scattered waves. The reason we choose only the first arrival is of practicality. The first arrival is often weak or incremental, and windowing the first arrival scales its contribution in the resultant correlogram, amplifying differential time peaks from correlations with the first arrival, which is what we want. The choice of 2 s is a balance. Choosing 1 s does not capture the full complexity of the P-scattered waves and also requires very precise picks of the first arrival, which is less practical. Windows longer than 2 s capture later, higher amplitudes and diminish the contribution of the first arrival in the correlogram. However, so long as the peaks corresponding to the converted phase are identifiable, the differential times do not change. We have added the text:

"We specifically window around the first arrival to amplify the correlation peaks with the P-wave, even if the P-wave is weak or incremental. The choice of 2 s balances our intention of

capturing the full complexity of the scattered wavefield associated with the first arrival while minimizing the contribution of later, stronger scattered waves."

Where: L483-487

Reviewer #2 (Remarks to the Author):

I read 'Sharp Structural Variability of the Gorda Slab Imaged by a Fiber Array' by James Atterhold and co-authors with great interest. The authors use local earthquake waveforms recorded at a fibreoptic cable deployed in Northern California to derive various structural parameters of the subducting slab crust at a smaller spatial scale than was previously possible. They first enhance signal-to-noise ratio of secondary phases using the pseudeo-autocorrelogram technique, then perform manual phase association and arrival time picking guided by sophisticated forward travel time modeling, and ultimately model the subsurface slab structure first in terms of slab surface depth and then in terms of spatial variability of the slab surface depth.

The work leaves me with fascination and a renewed sense of the great capabilities that fiberoptic sensing facilitates. I think it is valuable contribution to Nature Communications in the sense that it implements a variety of valid and correctly applied specialized processing techniques to the DAS data and gives impulses along various tracks of research that can build on top of this manuscript.

The work lacks some more detailed discussion of previous work, especially regarding the nature of the here imaged plate interface. Processing of some additional seismograms for the small-scale interface structure (Figs. 5 and S2) would greatly benefit the manuscript and illustrate the applicability of the method better. I know that this may be a lot to ask, but to my impression the demonstration of the capability of the method to deliver consistent and reliable results hinges on the comparison of the image of the (about) same structure using various, independent earthquake waveforms.

Finally, minor but important specifications regarding their phase nomenclature and spatial smoothing are required before publication.

We thank you for your kind words and helpful suggestions. We have endeavored to address each of your comments directly, and in doing so feel the manuscript is much improved.

* Important clarifications and specifications:

I find the phase nomenclature slightly confusing: A lowercase 's' appears to denote a direct, an uppercase 'S' a converted S-wave. No such distinction is made for 'P', where uppercase is used irrespective of conversion history. Perhaps the authors want to denote the direct P-phase with a lower-case 'p' as well? Moreover, the Moho conversion is denoted with a subscript 'M', but no such letter is given for the top of crust conversion. Perhaps a subscript 'T' (top) or 'C' (crust) would be appropriate?

Thank you for this comment. For simplicity, we have changed the phase nomenclature throughout the text and figures. Now, we define 4 phase conversions Ps (P-to-S off the top of the slab), Sp (S-to-P off the top of the slab), P_Ms (P-to-S off the slab Moho), and S_Mp (S-to-P off the slab Moho). We do not include a subscript for the "top of the slab" conversions, because whether that interface is the top of the slab crust, the top of the slab sediment, etc. is still ambiguous. However, we now state explicitly in the text, where each of these phases are first introduced, what these phases refer to.

"From here on, we refer to P-to-S and S-to-P conversions occurring near the top of the slab as Ps and Sp, respectively."

"From here on, we refer to P-to-S and S-to-P conversions near the slab Moho as P_Ms and S_Mp, respectively."

Where: L103-104 and L139-140

In line 475 the authors explain how they arrive at the spatial smoothing parameter, yet the smoothing that is actually applied is not given. As this parameter is very important to interpret Figures 5 and S2, I would like to ask the authors to provide some numbers regarding which spatial smoothing was actually applied.

Thank you for this comment. The defined length scale is 750 m. This is set according to the S-wave wavelength at 6 Hz near the interface (which has an S-wave velocity of ~4.5 km). The way this is incorporated is through fitting the Gaussian process. We have amended the text:

"This spatial smoothing is accomplished by fitting a Gaussian process to the data with a prescribed length scale equal to the wavelength, which we define to be 750 m (assuming a shear wave velocity of ~4.5 km/s near the interface)."

Where: L561-564

The notion of subducted sediments has been brought forward long ago, most recently e.g. by Han et al. (2016), Carbotte et al. (2023), Bloch et al. (2023). I think it would be important to introduce this concept of a more complex subduction interface already in the introduction of the article. Some formulations in the introduction are too vague (see general comments, below), and I think it would be important to introduce this issue early on, as it comes up in the discussion and can help to streamline this part. The discussion regarding the qualitatively different 'smooth' and 'rough' bathymetry might also be of relevance (e.g. Han et al. (2016), Carbotte et al. (2023))

Thank you for this comment, we agree that complexity at the interface beyond roughness is valuable to include earlier, and have modified the text to account for these other features that require high frequencies to resolve:

"Beyond roughness, complexity at the interface imposed by subducting sediments and shear further complicate the strength of the interface[4–6]. Characterizing fine-scale structure of the

subduction interface is necessary to address this problem, because it allows for the direct comparison of observed slip behavior to megathrust topography and rheology."

Where: L41-45

In a related issue, the authors already compare their larger-scale slab model with other studies in Fig. 3b. Yet, I think there are more comparisons that are possible and perhaps meaningful. Carbotte et al. (2023) imaged similar horst-and-graben structure similar to the one that the authors are imaging in their closest profiles PD19 and TD1918. It would be interesting to see if the lateral and vertical dimensions agree, by also plotting their data in figure 5 / S2. The picked bathymetry is openly available (Carbotte et al, 2024). Bloch et al. (2023) provide Receiver Function station measurements for the two nearby stations BK.ARC and BK.JCC. How does the depth of the two provided interfaces (b and t) compare to the depth of the found interface? Can we learn something from this comparison about the nature of the here imaged interface?

Thank you for these excellent suggestions. We have plotted the profiles in Carbotte et al. (2023) and have included those plots in this document. We do not see a clear "roughness" structure in profile TD19D18, other than the Rogue Propagator Shear Zone referenced (and annotated) in Figure 5 in Carbotte et al. (2024). However, we do see the clear horst-and-graben structure you reference in profile PD19. We focus on this structure because it is more analagous to the structure we describe in our study. Using the stack from Carbotte et al. (2023), which yielded more detail than the picked bathymetry we estimate the depth of the graben-like structure to be ~0.6 km in amplitude and ~6 km wide. This is certainly the same order of magnitude as our feature and a similar ratio of height to width as our observation. Because it's a different structure observed in a different part of the subduction zone, we choose not to plot it on the same plot as our profiles in Figures 5 and S2. However, we do plot it as a separate supplementary figure and discuss its similarities to our structure in the text:

"Similar structures have been observed in high-resolution, offshore active source profiles as well. For example, an active source profile off the coast of southern Oregon shows a narrow deepening of the plate interface[5] with a similar wavelength and amplitude to the bump observed in this study (Figure S3). This shows that these types of structures are present in images made using independent techniques and suggests that the bump we observe in our study is not unusual in Cascadia."

Where: L281-286

Added new Figure S3 to the supplement.

REDACTED

Figure R1: Plot of seismic reflection data for profile TD19D18 from Carbotte et al. (2023).

REDACTED

Figure S3. Short-wavelength interface roughness in seismic reflection data. Top: west-to-east depth-migrated reflection data from Profile PD19 in Carbotte et al. (2023) and Carbotte et al. (2024). Black dotted line outlines zoomed in section shown in the bottom plot. Bottom: Zoomed in view of the reflector associated with the plate interface in a zone of high interpreted interface roughness. Parallel white dotted lines, which are 1 km apart, bound a pronounced depression in the plate interface. Amplitude and wavelength of this depression are ~0.6 km and 6 km, respectively.

We appreciate the suggestion to look at BK.ARC and BK.JCC. The depth of BK.ARC for the t and c interfaces is 17.7489+-2.1 and 18.0057+-1.4 km, respectively. The depth of BK.JCC for these layers is 17.8141+-2.1 and 20.4539+-2.1 km, respectively. Both these interfaces are slightly deeper what we resolve with the DAS to the southwest (~15-16 km deep) (nearby stations south of the array are much shallower than BK.ARC and BK.JCC). What all these stations show us is that our results are much more in line with the t layer in Bloch et al. (2023). We now add this to the discussion:

"In general, when looking at receiver function studies that image both the top of the oceanic crust and a shallow layer interpreted to be the subduction interface[4], our results are more consistent with the interface depths."

Where: L352-354

The authors process fine-grained slab structures using secondary arrivals from two earthquakes. I personally find Figure S2 much more appealing than figure 5d, as I think it delivers a good sense of the capabilities of the method, comparing independent data. For how many more earthquakes are the authors able to compute a short-wavelength slab structural image? What is the 'worst' data quality that can still be processed? These are important tests for the more wide-spread application that the authors are suggesting in the discussion section. Also a comparison to data from Carbotte's (2023) profile PD19 and TD1918 data would be interesting and meaningful (see above).

Thank you for this question. It is certainly possible to compute a short-wavelength slab structural image for all earthquakes with Ps picks. However, the true strength of this methodology is in the relative slab depth within a profile generated from a single event. The other events with Ps observations do not have observations across the entire array, and so only produce short segments of slab depth variability. As is shown in Figure R2, these shorter profiles are not necessarily inconsistent with the longer profiles, but the first order feature in the comparison is the difference in absolute value, which we expect is due to the velocity uncertainty in the forearc (Figure S5). For this reason, we prefer to keep Figure S2 as-is, because the progression of the relative values is comparable between those two events due to the number of quality picks. We agree your suggestion to include Carbotte's profiles in the text is a very good one, and we have thus included a plot of one of these profiles in the paper (see comment above).

Note, the error bars for these curves are computed for the relative slab depths within each event, not the absolute slab depths. This is why the error bars do not overlap in Figure R2.

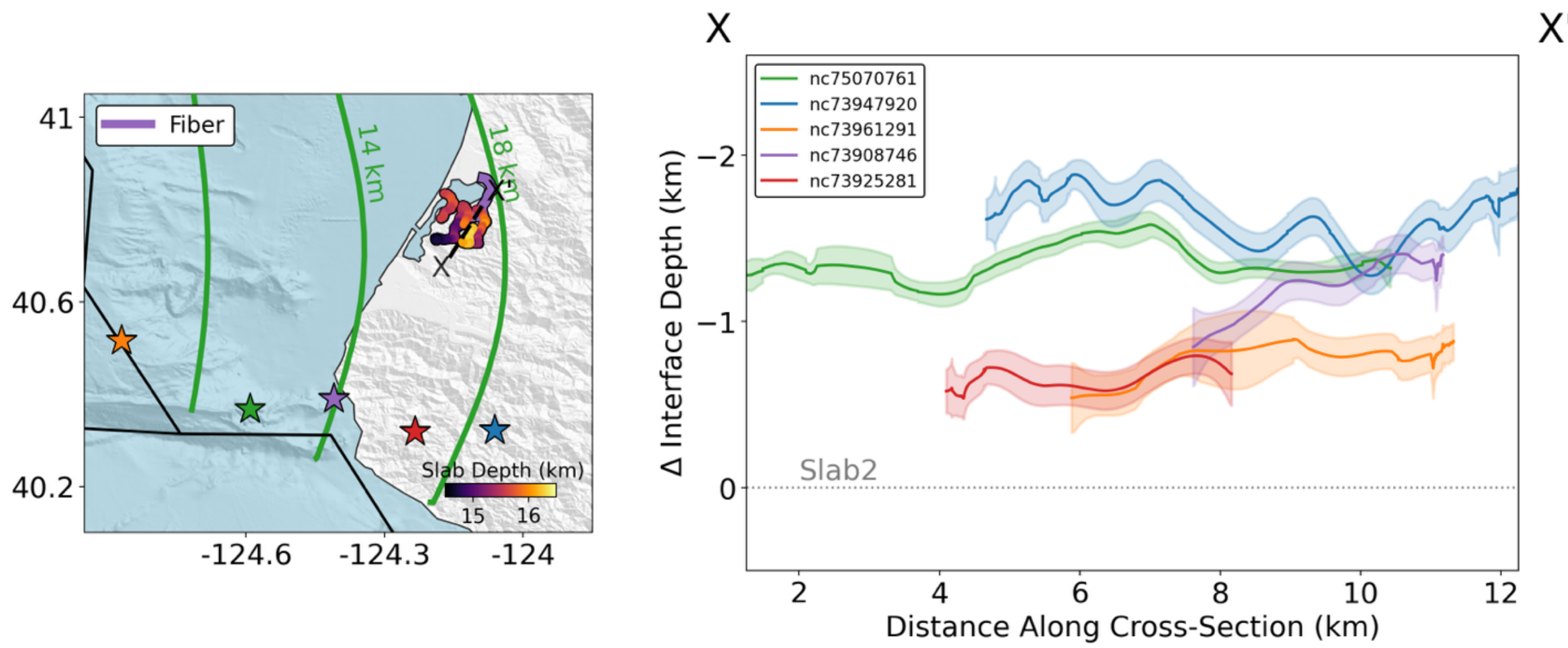

Figure R2. Same as Figure S2, but with additional events from Figure 3 included.

* Additional issues worth considering:

1. A-priori slab structure

The authors assert in various places that the Slab2 model is too deep in the area, yet they plot it prominently in most of their figures. I understand that it is important to be consistent with the processing (e.g. in Fig. 5d it is mandatory to reference Slab2, as this is the parameter they inverted for). Yet, to guide future studies, would the authors suggest to use another, better suited background model?

Thank you for this suggestion; indeed, it seems our results are most consistent with the recent study, Bloch et al. (2023), which both requires a shallower model than Slab2 (as we observe) also includes an estimate of the oceanic crustal thickness + an overlying layer that matches our interface-to-Moho thickness. This is also consistent with Beaudoin et al. (1994), though that is not a comprehensive Cascadia slab model. We now note this in the text:

"Overall, our results are most consistent with slab models that are slightly shallower than Slab2 and produce Moho-to-interface thicknesses that are larger than presubducted oceanic crust[4,26]."

Where: L261-263

2. Structural imaging of the Moho

The authors note in various places that they are also able to identify the Moho conversion. Is it possible to extend their analysis of the small-scale structures also to the Moho interface? I understand that this is a lot to ask, and likely goes beyond the scope of this study. Still, it would be good to know how far the authors have come and what would be needed to also construct a Moho model. One could use the Moho-model by Bloch et al. (2023) or Beaudoin et al. (1994) as

a starting point to compute synthetic PmS and smP (old phase nomenclature, please revise) arrivals. I would expect to see a much smoother image than in Fig. 5d.

Thank you for this interesting suggestion. Indeed, it is certainly possible to construct a profile like this for the Moho using our picks. However, we note that the Moho observations are lower frequency, do not cover the same distance along the array, and have higher scatter than the best conversions off the top of the slab (used in Figure 5 and Figure S2). Thus, the Moho observations generated in this study will not provide sufficient constraints to provide informative relative values of slab Moho depth. So, indeed we would like to leave this for future work with longer cables when better Moho observations are present.

* General comments:

l. 44-46. This last sentence of the paragraph is a bit unspecific to me. If space permits, can the authors give examples of what 'megathrust earthquake phenomenology' they are hoping to be able forecast through their findings?

Thank you for this suggestion. The preceding paragraph discusses the potential connection between interface structure and strength of the interface. The implication here is that by characterizing the interface, we can better understand the strength of the fault, which in turn, controls how ruptures might behave. To avoid redundancies, we would prefer to keep the paragraph as is.

l. 65 'enables meter-scale coverage over large distances': I think it would be important to be more specific regarding the state of the art realized in this paper. Over how 'large' distances are the authors able to achieve a meter-scale coverage? I think it is about 10km, judging from Fig. 5.

Thank you for this suggestion. In the methods, where we describe the data, we note that the array used in this study is ~15 km-long. But, the length of this array is limited by the fiber used, not the technique, which is capable of sensing over 100 km with just one unit (and there are telecom fibers that are continuous over these distances). We now give a more specific sense of the sensing distance the DAS units are capable in this section:

“This technique is powerful because it enables meter-scale coverage over tens to hundreds of km…”

Where: L67-68

l. 75 'Arcata, CA and Eureka, CA.' I think it would be good for outside-US readers to write out the 'California' abbreviations at least once.

Thank you for this comment, we now write out “California” instead of using the abbreviation.

l. 117: 'We can thus use the first arrivals of many earthquakes as template': Are the many template earthquakes not intraslab-earthquakes? It would be good to specify 'many' as 60, as suggested in the caption of Figure S1.

Indeed, the template events need not be intraslab earthquakes. We now note this in the text. We choose not to note that "many" means 60 here, because not all the templates were used for each event, and saying 60 here would be imprecise. We do, however, in the methods section, note that there are 60 template events.

l. 133: 'One' -> Once

Thank you, corrected.

l. 228: 'evidence that the slab near the array is too deep': it is the slab model (ref. 28) that is too deep, not the slab.

Thank you for catching this, corrected.

l. 242: Other explanations would be the addition of subducted sediments, as the authors asserted previously, or underplating of slab material (Sammis & Bostock, 2021; Bloch et al. 2023)

Thank you, we have modified the text accordingly to include these scenarios:

"These high measured thicknesses is could be due to subducted sediments, the altering of material in the forearc crust at the interface, the underplating of slab material, or a combination of these things, producing an apparently thicker crustal section."

Where: L259-261

l. 257: 'this bump is not present due to shallow structural effects' -> the bump is present, so delete 'present': 'this bump is not due to...'

Done, thank you.

l. 312: 'It also suggests' -> Reference of 'it' is unclear. Please write out.

Done, thank you.

l. 390: '100 km spatial window' -> Do the authors mean within 100km distance?

Indeed, this is what we meant. We have modified the text for clarity.

l. 418 'can be easily automated' -> I assume devil's in the detail here, and so would omit the 'easily'

This is fair, and we have replaced this phrase in response to another comment.

l. 433: 'the pseudo ray bending method employed in TomoDD'. I would ask the authors to give a

reference to the code used. Is it the same ray tracer used by 'simul' family (Eberhart-Phillips et al, 2024)?

Thank you for this comment. Indeed, this is the same ray tracer, but from the original version of the code. This is now referenced in the data acknowledgements section.

l. 461 - 465: The last two sentences are abbreviated beyond understanding. I would like to ask the authors to be a bit more verbose in their explanations here.

Thank you, this was indeed confusing. We have endeavored to make the description more clear:

"For these tests, we determine Ps arrival times by defining a model with P velocities below the interface and S velocities above the interface such that the time minimization path is equivalent to the Ps path through a standard model. For the Sp arrival times, we do the same but with S velocities below the interface and P velocities above the interface. For the Sp test, we ensure the source-receiver distance is sufficiently low such that the transmitted phase is the first converted Sp arrival. The model we use is the same 1D average model perturbed to generate the synthetic waveforms[22] with an initial Vp/Vs ratio of 1.73."

Where: L541-549

* Figure comments:

Fig. 1c) It would be good to plot the predicted 'sP' and 'PS' arrivals in as dotted lines, so the reader has a better chance to judge the data quality themselves.

Thank you for this suggestion, however dotted lines in this case have very poor visibility with the seismic color scale, so we prefer to keep solid lines for the arrival times.

Fig. 3a) The lines for sP and smP are indiscernible in the correlograms. I suggest using different colors / shades for each phase. The dotted, semi-transparent lines are fine, though. What is the solid black line in the correlograms? In the legend, I only see two black lines (PS and PmS), but on the correlograms I see three or even four. I would ask the authors to make the phase association clearer in the figures. It might be a good idea to place the labels directly next to the phases lines.

Thank you for these suggestions. We have modified the linestyles so that they are much more legible and distinct, and we have indicated the black lines (which are actually scatter points) are correlogram peak picks in the legend.

3b) I overlooked the legend at first sight. I think it would be important to place it in the frist subfigure as well and adapt the correct and complete phase nomenclature (pmS, SmP, and so on). The figure is missing a y-axis label.

Thank you for this suggestion. There is no y-axis label because these are density plots (and are all therefore normalized). To avoid confusion, we now include y-axis ticks and a y-label

indicating that this axis is density. As for the legend in 3b, we prefer to keep the legend as is, to avoid making the plots too busy.

Fig. 4b) The abbreviated velocity model titles are misleading. I suggest to refrain from using the ambiguous term 'crust' (it is unclear whether this refers to the forearc or slab crust), but use 'forearc' and 'slab' or 'oceanic' and 'continental' instead. Also, please provide vertical grid lines as to make the comparison of velocity models easier.

Thank you for these good suggestions. We have modified figure 4 accordingly.

* References

Eberhart-Phillips et al. (2024) Simul2023: a flexible program for inversion of earthquake data for 3-D velocity and hypocenters or 3-D Q doi.org/10.5281/zenodo.5746046

Sammis & Bostock (2021) A Granular Jamming Model for Low-Frequency Earthquakes https://doi.org/10.1029/2021JB021963

Carbotte et al., (2024), Depth to top of subducting Juan de Fuca/Gorda/Explorer plate crust and plate boundary fault within the offshore Cascadia seismogenic zone, 42°N to 50°N. Marine Geoscience Data System (MGDS). doi:10.60521/331666

Reviewer #3 (Remarks to the Author):

This review is for manuscript by "Sharp Structural Variability of the Gorda Slab Imaged by a Fiber Array" by Atterholt et al. The study analyzes DAS seismic data recorded by a 15 km segment of fiber optic cable originally deployed for telecommunication. It analyzes seismic records of 11 intraslab earthquakes in Cascadia subduction zone off the coast of Northern California. It identifies converted waves (PS, sP) from the slab interface and slab Moho. The technique used to improve visibility of these converted phases is a slightly modified version of a technique the authors have previously published about a different seismic phase. The dense spatial sampling of DAS data over a 15 km length allows to map the differential travel times (between converted phases and direct P wave) to continuous changes in topography on the slab interface which maybe useful to further study the role of roughness of slab interface in subduction zones in nucleation of megathrust earthquakes. The study also makes interpretations on amplitudes of the converted waves by comparing with synthetics calculated with different 1D velocity models that may reflect different state of the forearc crust and slab near the interface.

I recommend major revisions. The objectives and the results are noteworthy and significant, though some results (based on differential times) feel more reliable than others (based on amplitudes which may depend on lot more factors like attenuation and 3D structure) but I have nothing concrete against anything. The method is similar to the method authors have previously published (Atterholt and Zhan) but the observations and findings are new and different in context and study area (subduction zone vs southern CA).
The study is kind of vague and silent on many technical details which kind of make me slightly doubt the reliability of results.

Thank you for your review of this manuscript and for providing helping comments. We have substantially amended the discussion and methods section to improve the clarity and completeness of the technical details in the manuscript. We think the manuscript is improved as a result.

Major comments -

- The inversion part is the most confusing and vague part of the study.

L467-472: I didn't understand what functional space means. My interpretation is that they have calculated differential times for 3 different slab depths and are simply linearly interpolating the observed differential time to a slab depth measure.

Thank you for catching this, you are right that functional space is not a good descriptor of the way we perform the mapping. We have changed "functional space" to "linear interpolation function" or "linear interpolant" throughout the text.

"To invert for a modified slab geometry, we use expected arrivals from 3D raytracing for a range of slab depths to define a linear interpolation function that maps slab interface depth and slab Moho depth to phase differential time. We can then search this interpolant for the slab parameter depths that best fit our observations (see Methods).

Where: L220-223

"We again search a linear interpolant to determine slab depth…"

Where: L268

"We use these computed differential times to define a linear interpolation function between absolute slab depth and phase differential time at each conversion point."

Where: L555-557

L473-476: There is some smoothing applied to the results along the array but it doesn't say what the wavelength of the smoothing is. Figure 5d: Some uncertainty (shading) is shown around the results but I didn't find anywhere how it was calculated.

Thank you for pointing out that this information was missing. We have modified the methods to include these details:

"This spatial smoothing is accomplished by fitting a Gaussian process to the data with a prescribed length scale equal to the wavelength, which we define to be 750 m (assuming a shear wave velocity of ~4.5 km/s near the interface). The standard deviation on the picks, which constrains the Gaussian process error, is assumed to be 1/4 the period of the correlation peak (assuming a frequency of 6 Hz) for the best constrained picks (nc75070761) and 1/2 the period of the correlation peak for the more poorly constrained picks (nc73947920)."

Where: L561-567

Figure 3b, L211-212: I didn't understand how measurements from individual channels are turned into these distributions. There is no y-axis on the plots. What does "slab parameters" mean here? For the other studies that are cited in Figure 3b, how did you get the ranges? Did you extract the slab depth at your conversion points from the models in these studies and are plotting the range?

Thank you for requesting these clarifications. Firstly, we'd like to clarify that the distributions are density distributions (thus normalized and comparable between events). For clarity, we now plot the density y-axis for these plots. The distributions are obtained by performing the inversions for change in slab depth and crustal thickness using every observation from an individual event, which yields a range of values. To clarify these points, we have also added the line to the figure caption:

"Each distribution is shown as a normalized density and includes inversion results from all observations from an event."

We have also changed the "slab parameter" language to make the referenced lines more clear:

"For all autocorrelograms, we combine all the inverted slab interface and Moho depths into a distribution of solutions that together can fit all the data. This approach prioritizes the high temporal frequency phases that were confidently identified and classified using the spatial density of DAS to infer slab properties with high precision (~half the period of the correlation peak) and a measure of uncertainty."

Where: L225-230

To get the ranges from other studies shown in Figure 3b, we took a narrow spatial window that includes the locations of the interface measurements shown in Figure 1a, and subtracted Slab2 from these referenced models to get a range of differences between models for the area of interest. We now include the text:

"We can compare these results to previous studies by subtracting Slab2 from those models in a narrow spatial window encompassing the measurements made in this study. We find that our results are consistent with..."

Where: L238-241

- Figure 4c: The comparisons of waveforms is not really fair as the DAS waveform is actually a pseduo-autocorrelogram whereas the synthetics are strain synthetics. For apples-to-apples comparison, you should correlate the P-wave with the entire waveform for the synthetics and show that.

Thank you for this suggestion. Indeed, the DAS is a correlation-based measurement and the nodal data shows the inertial waveforms. We agree that the synthetics are better represented as autocorrelograms, and we have modified Figure 4c and 4d accordingly. For the nodes, because

they are inertial measurements, we feel that the autocorrelogram representation, without the strong scattered wavefield which is only present in strain data, does effectively show the phase conversions, and so we choose to represent the nodal data as-is.

In this figure, the DAS waveform is aligned with the peak of P-wave packet at t=0 but the synthetics are aligned with the first onset at t=0. This is inconsistent and may make the reader doubt the reliability of the results.

These are actually consistent. One way to think about this is that the differential times in the autocorrelograms correspond to the difference in onset time between the P-wave and the secondary phase. So, the zero-phase peak in the correlogram should be paired with the onset of the P-wave and the subsequent peaks in the correlogram will correspond to the onset times of secondary phases. That said, the synthetics are now autocorrelograms, and the zero-phase peak for those is now aligned at time zero. The nodal data are still represented such that the initial onset of the P-wave is at time zero.

L637-638 Please show the actual waveform you are using to make the measurements instead of a 1 km average. Otherwise it paints a false picture of quality of measurements

We appreciate this suggestion, but this particular plot is not meant to represent data quality (for that, we represent almost all data used in this study in Figure 3), but instead is meant to capture the differential times and relative correlation peak amplitudes of the phases. For this, a 1 km average produces a cleaner, more representative result.

L169: You are drawing conclusions about the M4.8 event here using waveforms for M5.3 event. Given you have shown that composition of the wavefield could depend on source-receiver distance (Figure 4d) and those two events are at different distances, maybe you should also show similar synthetics for the M4.8 event in the electronic supplement. I don't think the results will change but you should do it to dispel all doubts.

Thank you for this suggestion. We note that the difference between the M4.8 and M5.3 synthetics are marginal as is shown in the attached figure (reproduction of Figure 4d).

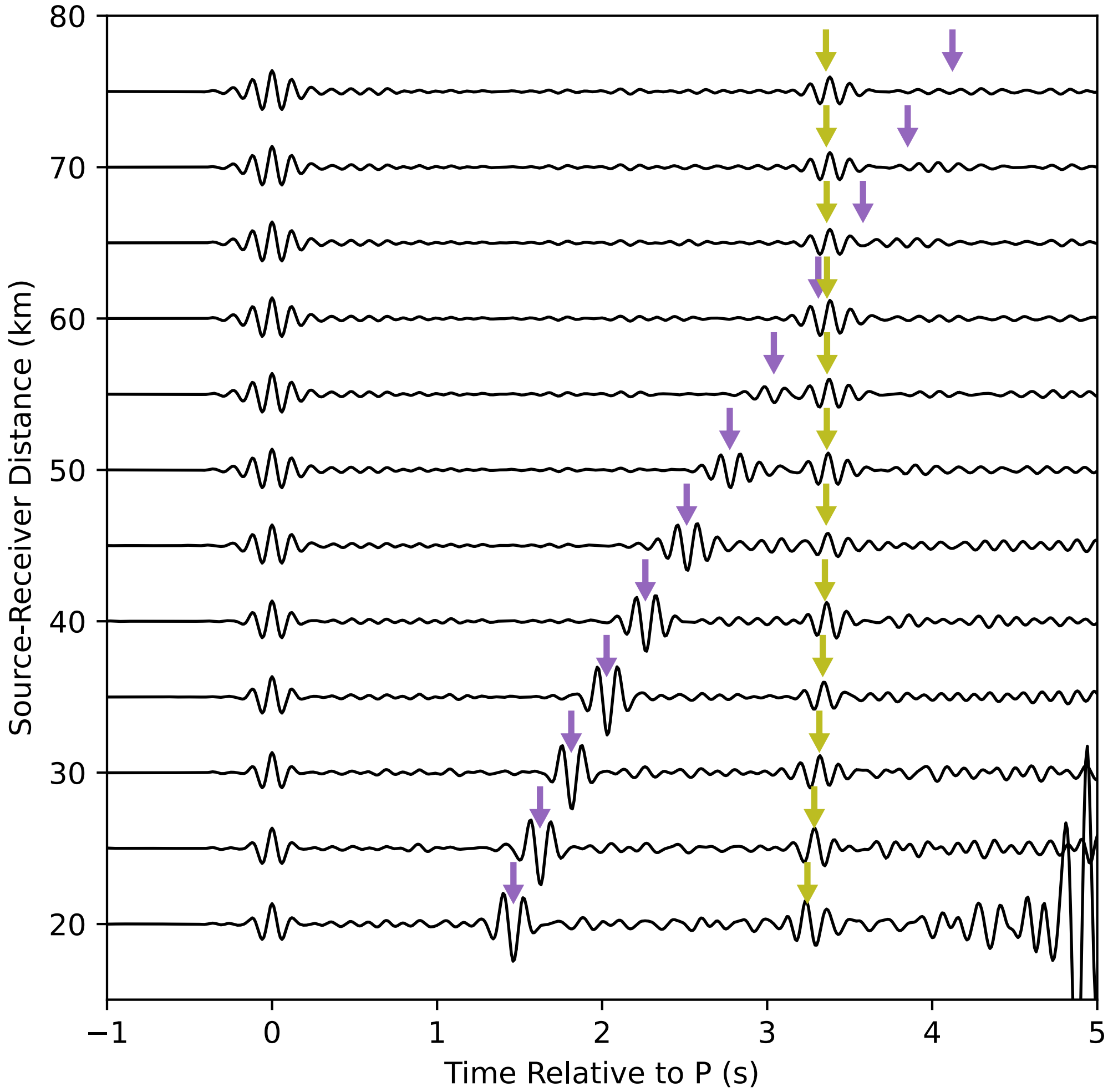

Figure R3. Reproduction of Figure 4d with the focal mechanism and depth of the M4.8 earthquake.

We thus choose not to include this in the paper, but we note that this raises an important point that we now include in the paper. The source-receiver distance between the M4.8 and M5.3 are different enough to be significant in terms of the observability of Sp. We now note this in the text as an alternative explanation to a sharp difference in the distribution of fluids:

"We expect, then, that for earthquakes more than 50 km away from the array, we would be much more likely to observe Ps rather than Sp. This matches our observations well and makes comparing the amplitudes of Sp between events challenging. This also provides an additional phase classification metric for when the expected arrival times of Sp and Ps are coincident. This also offers an alternative explanation for lack of observable Sp phase in the Mw4.8 earthquake. The Mw4.8 is further away from our array than the Mw5.3 (~46 km versus ~55 km). The

distance range between 46 and 55 km encompasses a the sharp decay in amplitude of Sp observed in our synthetics in Figure 4d."

Where: L196-203

L172: "for most of our observations", L190-191: The synthetics shown are for an event that is an exception. You should also show synthetics for an earthquake that represents most of your observations in an electronic supplement.
For the nodal seismometer waveforms, all three components must be shown.

Thank you for this comment. We chose to represent the M5.3 in Figures 4a and 4c because this earthquake happened during the nodal deployment. However, our synthetics are not only representative of this exceptional event. The set of models in Figure 4b were included so that we could have a large enough diversity of synthetics to explain the range of observations in this study. The synthetics from model (iii) included in Figure 4b (the waveform for which is shown in Figure 4c) reproduces most of our data. That is this model produces a positive relative polarity Sp peak and a negative relative polarity Ps peak, explaining our other interface conversion observations shown in Figure 3. This is noted in the text:

"This would suggest that the other observations are more consistent with phase conversions generated by a model of a fast crust and hydrated slab (model iii; Figure 4)."

Where: L206-208

- If possible and considered useful by authors, I would like to have a short discussion added on the consequences of ignoring finite-frequency effects. Even though you are saying your observations are high-frequency (> 6 Hz) and wavelength is <750 m (L302), this interpretation is probably meaningful for direct body waves. Your measurements are correlations (P waves and remaining wavefield) and stacks of correlations across different earthquakes. Correlations are known to result in sidelobes instead of a simple wave packet if the assumptions are not satisfied making it difficult to precisely pick an onset. The coda not only contains contribution of heterogeneities at the receiver position, but also at the source position that may be different for earthquake pairs with different locations and radiation patterns. Some phase conversions are weak (for example, 73947930, 74001081, 73961291), some show other phases diverging off (73827571). The saturated colors make it little bit challenging to judge how sharp the peaks of converted phases are in the correlograms. If an onset is too emergent, you can follow a peak or trough and you can measure the relative variation of differential traveltimes of the converted phases because they are coherent from channel to channel but then the interpretation is from a wave packet that is fraction of a second width (> 0.2 s) even though your travel time variations are < 0.1 s.

Thank you for this comment. To your first point, our incorporation of finite frequency effects is a pragmatic simplification of these effects with limitations, and we now include a lengthy discussion of when this approximation might result in inaccurate results and how our interpretation is informed by this (see response to major comment 1 from Reviewer 1 above):

“Additionally, the time-minimization geometric raytracing that we use is an approximation that does not include finite-frequency effects. To partially account for the finite bandwidth of the wavefield, we smooth the inverted interface by the wavelength of the converted phase in the neighborhood of the conversion. But this approach, which is a pragmatic simplification of commonly used methods in tomography[35], can be inaccurate in the presence of strong structural heterogeneity. In some cases, structural complexity sensed over a finite bandwidth can shift the distribution of sensitivity away from the time-minimization path. This problem is especially acute when the interface topography varies at scales similar to the converted phase wavelength of 750 m[36]. For this reason, we are careful to only interpret structures that are several multiples larger than this scale. Using Sp and Ps finite-frequency kernels would produce a more accurate result[37]. Frechet kernels for Sp an Ps amplitudes typically have high and uniform amplitude over scales of a few km[38]. The computational expense of computing these kernels at the frequencies used in this study is large and we do not yet have a high-resolution starting velocity model to work with. We thus save the implementation of that approach for future work.”

Where: Lines 401-415

We also appreciate your second point, which we interpret as a question of whether or not we are picking the true correlation peak or side lobes. We would first note that our stacking across different earthquakes is robust. This can be seen very clearly in the zero-phase correlation peaks. These peaks are the basis for the stacking we perform, and as is shown in Figure 3, these zero phase peaks are, broadly, highly concentrated on the central peak and symmetric about this peak. From this, we expect that the stacking was successful and the ambiguity that exists would arise from which peak (or trough) we pick the maximum (or minimum) of on the often much weaker secondary phase differential time peaks. For the strong peaks, this is not a problem, but for the weakest peaks, this is a source of uncertainty. This is partially why we show as much of the data used for the slab imaging as possible in Figure 3, so that the reader can see the variability in differential time peak amplitude and clarity. We agree that noting this in the text is useful, and have added the following discussion:

“In some cases, uncertainty can arise due to the picks themselves. While picking correlation peaks is highly precise, for the weakest differential time peaks, for example Sp in event nc73947930 in Figure 3, there is some ambiguity in whether we are picking the true correlation peak or an adjacent side lobe. In these instances, there is generally a clear candidate for the true correlation peak based on the amplitude of the peak and the symmetry of the side lobes about the peak. While the relative differential times within the event are minimally affected by this, this ambiguity can lead to uncertainty in the relative polarity of the secondary phase and the absolute differential time, generally by half the period of the secondary phase.”

Where: L381-389

- All waveforms and record sections look somewhat bandlimited to me in the figures. Even though it is mentioned many times that you use high-frequency measurements (> 6 Hz) (L295,L302), I didn't find any mention of a bandpass filter anywhere (my apologies if I have missed it). Please write if you are applying any filter.

Thank you for catching this omission, we are using a filter, and now include the filtering window in the text:

"After applying spectral whitening and bandpass filtering the data between 1 and 10 Hz…"

Where: L480-481

- A lot of quality-related terms have been used (L214: precision, L215: uncertainty, L243: very-high quality, L391: signal-to-noise ratio) without any quantitative definition anywhere.

Thank you for pointing these terms out. We have made some changes to better articulate what these terms mean:

For "precision," we now include in parentheses: "(~half the period)" to clarify

Where: L229-230

"Uncertainty" refers to the width of the "distribution of slab solutions" referenced in the preceding sentence and shown in Figure 3b.

For the choice of "very-high quality" we add that this was "determined visually"

Where: L264

For "signal-to-noise ratio," we have changed that sentence to be clearer:

"We visually evaluated each of these earthquake wavefields, and we selected 60 earthquakes with clear P-wave onsets and little traffic noise as template events to be used in the subsequently described methodology."

Where: L453-454

Minor comments -

- For discussion of relative amplitudes (L149-204) from synthetics, I didn't find any info on the density and attenuation structure assumed (my apologies if I missed). L457: FK integration requires layered structure I think, write what is the thickness of layers used to discretize the models in Fig 3b. Write source duration.

Thank you, we now include this information in the methods:

"Each model is defined using 1 km-thick layers, a uniform attenuation structure ($Q_\alpha = 2Q_\beta = 1000$), and a delta source time function."

Where: L521-522

- L82: Gorda slab off the Northern California coast

Thank you for this suggestion, we have removed "the southern portion of" but, since the array is onshore, we chose to keep this as "above the Gorda slab"

- L93: similar source mechanism and similar depths

Thank you, we have made this change.

- L103: surface waves scattered off the direct arrival near the surface, rather than the direct arrival this is just to indicate this is not the main surface wave getting scattered

Thank you, we have modified the sentence accordingly:

"Picking can be even more difficult in DAS data, because phase arrivals are often dominated by surface waves scattered from the direct phase due to shallow structure, rather than the direct arrival."

Where: L106-108

- L110: earthquake wavefield -> P-wave wavefield ?

Corrected, thank you:

"…earthquake wavefield following the P-wave…"

- L126: number not reported in other studies -> add reference here

Thank you, we have added citations for this part.

- L176: little bit counter-intuitive to me why marine sediments and finite fault thickness would make it faster. maybe expand/explain in a sentence.

Thank you for pointing this out, we agree with your intuition and the expectation for these layers is that they are slower than the overlying forearc crust is determined empirically. We have modified our discussion to recognize this accordingly:

"This is within the range of expected thicknesses for subducted marine sediments[25,26] or finiteness of the megathrust fault due to shear[4,6], but these layers are generally expected to have lower velocities than the overlying forearc crust[4,27]. More work needs to be done to determine the nature of this boundary; however, this suggests that the high frequency content of our measurements allow for imaging narrow layers at depth…"

Where: L184-189

- L226: cite Fig S4 here

Done. Thank you.

- L238: cite Fig 3b here

Done. Thank you.

- L331: write how much is the Vp/Vs ratio in the model in the top 5 km below the array so people have a reference on how good or bad it is

Thank you, we now include this information in the methods section. The Vp/Vs ratio of the initial model is uniform:

"The model we use is the same 1D average model perturbed to generate the synthetic waveforms[22] with an initial Vp/Vs ratio of 1.73."

Where: L548-549

- L389: 100 km spatial window is a bit vague,

Thank you for this comment. We changed the language to "within a distance of 100 km" for clarity.

- L394: write the name of the catalog. Cite Table S1 here.

Thank you, we now reference the catalog (NCSN) in a preceding sentence and cite Table S1 here.

- L414: make it clear whether this is observed pick or pick predicted by the velocity model

Thank you, we have clarified this in the text. These are computed picks, but we allow for shifting of the picks following visual inspection:

"After applying spectral whitening and bandpass filtering the data between 1 and 10 Hz, we take a 2 s window around the computed first arrival, which is shifted by visual inspection if necessary, of each of our template events and candidate events …"

Where: L480-482

- L424-425: this can be dangerous, if two events are too far apart or have different mechanisms, different phases can have different polarities (like, P waves have same polarity but converted waves may have different polarity)

Since only the P-wave wavefields from the templates are used, correcting the polarity according to the correlogram between the template P-wave and the intraslab earthquake P-wave is sufficient. In other words, we are only correlating P-waves from a set of events with *one* event wavefield that may contain observable converted phases. All the P-waves used will have the same polarity to uncover the converted phase observation that comes from the event of interest, and so what the polarities of possible converted phases in the template event wavefields does not matter.

Figure 1a. What are the black lines? How do you convert the conversion points to the squares? Add degree symbol to geographic coordinates

Thank you for pointing these things out. We have modified the figure caption:

"Black lines are plate boundaries defined in Bird et al. (2003)[44]. Also plotted as square points are changes to Slab2 resolved in this study (Figure 3b[4]) at the average location of the conversion points used to make the estimate (see Methods)."

Figure 1c. Write if t=0 is origin time

Done. Thank you.

L611, L616 scattered wavefields -> P wave window

Corrected. Thank you.

Figure 4b. This plot maybe more informative if it has two subplots with one showing all Vp models plotted on top of each other and one showing all Vp/Vs models. Move the Vs to electronic supplement as your primary parameters for constructing the models are Vp and Vp/Vs. Minor grid so people can judge values.

Thank you for this suggestion, but we would prefer to keep the plot as-is, in-particular because we think the representation of models (ii) and (iii) are more informative when you can see both profiles.

Figure 4c. The right two dotted lines are for ? The subscripts in names of converted phases are not explained - wz, conv, refl

Thank you for noticing that these were not described. We now define the purpose of these lines and explain the phases included in the figure caption.

L645 lateral sensitivities -> conversion points

Done. Thank you.

Figure 5d write what is the range of differential travel times in the text for producing this range of perturbations of slab interface depth

Thank you for this suggestion, we now include this information in the figure caption:

"The differential time picks ranged from 2.7 to 2.9 seconds."

Figure S1b plot both positive or negative difference rather than absolute value

Thank you for this suggestion; however, we prefer to keep the absolute value, because it allows us to use points backed by larger data subsets.

Figure S1c maybe difference in backazimuth from array to the two earthquakes is more meaningful?

From my understanding of this comment, I think this is what we are doing. We compute the azimuth of the template source to the array and the event source to the array and take the difference.

Figure S4 I am not sure how the quantity in colobar (depth) is different from event depth and slab depth (two axes)?

Thank you for pointing this out, this was indeed confusing. The colorbar represents the magnitude of the change in depth at a given point, and each of the axes represent the change if differential time if you change the hypocenter depth or the slab depth by the value given by the colorbar, respectively. We have added the following text for clarification:

"The axes represent the change in differential time due to changing depths, and the colorbar represents the magnitude of the change."

Figure S5 Which 1D model was assumed? What was the source depth?

Thank you, we now include this information in the methods section:

"The model we use is the same 1D average model perturbed to generate the synthetic waveforms[22] with an initial Vp/Vs ratio of 1.73."

Figure S6 source-receiver distance and source depth?

Thank you, we have added this information to the figure caption:

"For these tests, the source depth was set to 26 km and the source-receiver distances ranged between 55 and 75 km."

Reviewer #2 (Remarks to the Author):

This is my second review of 'Sharp Structural Variability of the Gorda Slab Imaged by a Fiber Array' by James Atterhold and co-authors.

The manuscript was challenged by three reviewers, where the comments of the other two reviewers pertained mostly to methodological aspects while my own review focused on the interpretation. From reading the rebuttal letter, I feel the authors have undertaken an apropiate effort to resolve all reviewer's concerns. The manuscript has methodological emphasis, with a clear motivation and very well demonstrated application.

Thank you for your thoughtful review and additional comments. We have endeavored to address all comments made.

As the other reviewers noted, the method is often heuristic (time window selection, peak detection, selection of high-quality observations). For this reason, I would argue it is warranted to evaluate the quality of the results in a similarly heuristic manner and would like to ask the authors to exhibit more than only the best results, that is show the data of figure R2, at least in the supplement, if not in the main text.

Thank you for this comment. We now include this figure as a supplementary figure and note that the other Ps observations are not extensive enough to corroborate the bump in the text:

New Figure S3.

"Resolving this feature requires precise measurements of relative differential times with sensitivity across the bump. Other Ps observations, while not inconsistent with this feature, are not extensive enough to corroborate it (Figure S3)."

Where: Tracked changes document: L284-286

Finally, some parts of the manuscript still have an overly colloquial tone that sometimes makes it difficult to understand the full meaning of what is written. Examples of this are given below.

After the correction of these minor imprecisions I would like to recommend the manuscript for publication in Nature Communication.

Examples of inaccurate language:

l. 58 phase conversions off the slab interface -> I think 'off' should this rather be 'of'? Please double check

We have changed "off" to "from" here.

l. 59 are *of* high-frequency

We have changed "are high-frequency" to "have high frequency contents."

l. 70 'increasingly ubiquitous' is a tautology

We have changed “increasingly ubiquitous” to “increasingly widespread.”

l. 153 its conversion is *of* very high amplitude

Changed. Thank you.

l. 222 search this interpolant for the slab parameter depth -> lookup interface depth?

For precision, we would prefer to the language as is, because the method is a grid search inversion and the interpolant references the linear interpolation function described in the preceding sentence.

l. 259 a combination of these things -> of these processes

Done. Thank you.

l. 454 We chose 10 km because we wanted to ensure -> We chose 10 km to ensure

Done. Thank you.

l. 498 omit 'for this'

Done. Thank you.

l. 507-508 This sentence should be expressed in propper language: 'The grid points that minimize the P to grid + S from grid and the S to grid + P from grid travel times yield approximate Ps and Sp converted phase travel times.'

perhaps as:

The grid points that minimize the travel time of (a) the P-wave to grid plus the S-wave from the grid and (b) the S-wave to grid plus the P-wave from the grid travel times yield approximate Ps and Sp converted phase travel times.

We have changed this sentence to:

“The grid points that minimize the travel time of the P-wave to the grid plus the S-wave from the grid and the S-wave to the grid plus the P-wave from the grid yield approximate Ps and Sp converted phase travel times, respectively.”

Where: Tracked changes document: L521-523

In a similar manner ll. 517 - 523 may be better expressed as a propper list.

We prefer to keep this part as-is because it introduces these models in a similar way to how they are represented in Figure 4.

Reference 2 is ill formatted

Thank you for identifying this. We have corrected it.

Reference 16 was unavailable at the time of review.

The data repository this is referencing is now online.

Ref 17 is missing a Journal

Corrected. Thank you.

Reviewer #3 (Remarks to the Author):

This is my 2nd review of the study "Sharp Structural Variability of the Gorda Slab Imaged by a Fiber Array" by Atterholt et al. This revised paper is significantly improved. The authors have worked hard to address the reviewers' comments and concerns. I recommend only minor revisions (typos and minor clarifications) after which the paper should be accepted. I don't need to look at this paper again.

Thank you very much for your additional review and helpful comments. We have addressed each comment below.

MINOR COMMENTS

L166-169: the roman numerals should come before the description of the model for consistency with elsewhere in the paper

Done. Thank you.

L179: faster forearc crust?

Yes, thank you for catching this. We have changed it.

L279: by two events on different channels can you write the specific range of channels/along-fiber distance for these two events here in parentheses.

Done. Thank you.

L328: geology -> geology and seismic velocities

Done. Thank you.

L408: an -> and

Done. Thank you.

L517-525: similar to how you changed the abbreviated titles of the models in Fig 4b in response to reviewer 2's comments, I recommend similar changes here as well to clarify if the crust is forearc or slab crust. For example, slow crust -> slow forearc crust, etc.

Thank you for catching this. We have made these changes.

L553: interface -> interface and a frequency of ~xx Hz.

Done. Thank you.

Figure 3 caption, L724-726: Green/black picks/dotted lines, I think this was for the previous version of the figure. The caption should be corrected.

Corrected. Thank you.

Figure 3 caption, L725: 3D raytracing -> 3D raytracing and the reference interface model. You should make it clear whether you are using the reference interface model or model incorporating your new corrections for raytracing, otherwise readers will be confused. Same for Figure 5c.

Done. Thank you.

L739: S8 -> S9

Corrected. Thank you.

L742: 12-13 km along-fiber distance

Done. Thank you.

ELECTRONIC SUPPLEMENT

L39: line -> polygon

Corrected. Thank you.

Fig S5: The phase symbols sP, PS in both x-axis and y-axis need to be corrected to be consistent with elsewhere in the paper

Corrected. Thank you.

Fig S5 caption, L65: equivalent -> numerically equal ?

Corrected "equivalent" to "equal."

Fig S5 caption, L67: magnitude -> amplitude or size

Done. Thank you.

Fig S6 caption, L77, L79: inaccuracy -> it would be good to change it to underestimation or overestimation (based on the numbers) to be clearer

Thank you, we have changed "inaccuracy" to "underestimation."

Fig S6 caption, L79: cersus -> versus

Fixed. Thank you.

Table S1: Mark the 11 events actually used for the phase conversions with a star.

Done. Thank you for this suggestion.

Reviewer #4 (Remarks to the Author):

This manuscript by Atterholt & coauthors presents an innovative application of DAS to detect slab-converted phases and infer slab structures. The quasi-autocorrelogram stacking technique is interesting and the study area is relevant. However, I'm not 100% convinced this manuscript is a contribution at the Nature Communications level, as I believe more observations in different contexts, maybe with longer cables might be needed to define conclusive these findings. Nevertheless, as far as I know, this is the first tentative imaging of slabs with converted phases recorded by DAS, thus impressive, if one thinks about the possibility that opens for monitoring oceanic slabs with typical offshore cables (perpendicular to the trench) and onland cables (parallel).

Thank you for taking the time to review our paper and for providing such thoughtful comments. We have taken steps to address each of your comments.

Major Comments

1. Observations might be inconclusive for the claims

The primary results rest on converted phase observations from 11 events, however with high quality imaging tentative from effectively 2 events (nc75070761 and nc73947920; Figures 5 and S2). The correspondence between these two estimations of the slab topography bump is encouraging. As it stands, the paper demonstrates the method has the potential to image slab structures, but I would suggest relaxing the conclusions: this method must be demonstrated in other contexts. As far as I know, there are other oceanic DAS cables above subduction zones. There might be a lack of converted phases in these contexts, but the authors might mention this possibility. If demonstrated in another context this would be really a major scientific contribution!

Thank you for this comment. We agree that the applicability of this method more broadly can be made clearer in the paper. Indeed, southern Cascadia is not the only place where these converted phases from local earthquakes are observable, and we now reference over a dozen studies that demonstrate the observability of these phases in different, important subduction zones (Alaska, Mexico, Japan, New Zealand) and other parts of Cascadia. We also now include references to several examples of other current and past DAS deployments in the vicinity of subduction zones (central Cascadia, Alaska, Japan, and Chile). We believe this demonstrates there is already great potential to apply this method more broadly.

“Moreover, there are thousands of kilometers of onshore and offshore fibers in subduction zones that could potentially be transformed into DAS arrays, several of which are already being sensed or have been in the past[40–43]. This technique can be applied wherever there are intraslab earthquakes and structural discontinuities at the slab interface, which is not unique to southern Cascadia. These phases have been observed in Alaska[44–47], Mexico[48,49], Japan[50–53], New Zealand[54,55], and other segments of Cascadia[56,57]. This means that repeatable high frequency measurements of the slab interface can be made at depth in subduction zones with diverse geology and phenomenology.”

Where: Tracked changes document L438-443

We would argue that we give extensive treatment to the uncertainties and challenges of the methodology in the “Challenges and Uncertainties” section of the paper. This includes thorough descriptions of phase classification nonuniqueness, tradeoffs with the velocity model and hypocenter location, finite frequency effects, and correlogram picking uncertainty. In the context of all of these, we describe the bump interpretation as our preferred explanation for the data, but not the only explanation. Here’s an example:

“This inaccuracy would likely need to be kilometers deep and narrow (Figure S8). This is conceivable, but given that our observations can be verified across events and at different channels (Figure S2), we prefer the interpretation that the most prominent features are indeed slab topography. Uncertainty in absolute slab depth can map into uncertainties in relative slab depth, because the absolute slab depth controls the locations and incidence angles of the conversion points. However, these effects are minor and do not change the results of this study (Figure S5). These tradeoffs show that short-wavelength slab topography, which may be important for the next generation of slab models, is best resolved with accurate and precise 3D velocity models with sensitivity to the full crustal section.

Where: Tracked changes document L382-390

The fundamental observations, though, can be made with high confidence from the results in this study. That is: 1. We are observing a diversity of converted phases attributable to the slab interface across many events. We verify this extensively with 3D raytracing in a region where these phases are known to populate earthquake wavefields (Gong & McGuire, 2021). 2. We have developed a methodology to amplify and pick the arrival times and polarities of these converted phases with high precision. The methodology itself is very simple and close to the data (i.e., minimal processing is required, and the observations are visible in the raw data). This methodology is demonstrated through the representation of many wavefields and correlograms. *Together, these contributions extend photonic seismology to subduction zone imaging.* Given the challenges involved in imaging megathrust faults and processes, the importance of these faults for great earthquakes, and the opportunities presented by telecommunication networks for imaging these structures in hard-to-reach places, we feel this study constitutes a contribution at the Nature Communications level.

2. Circularity in the spatial interpretation of conversion points for raytracing
The conversion points are computed by raytracing to the Slab2 interface model. However, one of the results is that Slab2 is too deep by 1 -3 km (fig 1 c). I might have misinterpreted this result, but it's a sort of circular problem, isn't it? I had some difficulties in trusting the described topographic slab bump, which is smaller (or similar) in dimension to this mismatch. If the reference model depth is found not correct, then the incidence angle is supposedly incorrect and as a consequence also the conversion podsint. Is there a way to estimate the slab geometry error or iterate the inversion to address this?

Thank you for this comment. There are two points we'd make in response. 1. The way we do the inversion is we compute the expected slab conversion arrivals for multiple slab depths (that is, we shift the Slab2 model up and down and perform 3D raytracing to compute arrival times for interfaces at these different depths). By computing the expected arrivals for different slab depths, we account for differences in incidence angle across different slab depths directly. 2. Indeed, the uncertainty in the absolute depth of the slab is higher than the relative uncertainty. This is because of tradeoffs between the absolute slab depth and both the hypocenter depth and long wavelength inaccuracies in the 3D velocity model. We discuss this at length in the paper and perform corresponding sensitivity tests.

However, there would be a bump along the profile regardless of the absolute depth. Changing the absolute depth can affect long wavelength behavior across the profile, but the short wavelength variability is preserved. To demonstrate this, see Figure R1. This figure shows the expected arrival times from raytracing for very different slab depths, which produces clearly different absolute differential times. However, if we subtract our observed differential times from each of the differential time profiles for different slab models, the short wavelength variability in the difference is preserved regardless of the initial slab model. In particular, the bump along the profile is informed by the depression in the difference between observed and expected times indicated by the gray arrow along the profile. This feature is preserved regardless of the absolute slab depth. An iterative inversion is something we are exploring for follow-up studies. However, the main drivers of uncertainty in the absolute depth are the hypocenter location and the velocity model. An iterative inversion would need to update the event location, the velocity model, and the slab depth. This requires building a new tomography code, which is beyond the scope of this study.

We now mention this in the text and have added a supplementary figure (Figure S5) to show this.

"Uncertainty in absolute slab depth can map into uncertainties in relative slab depth, because the absolute slab depth controls the locations and incidence angles of the conversion points. However, these effects are minor and do not change the results of this study (Figure S5)."

Where: Tracked changes version: L385-388

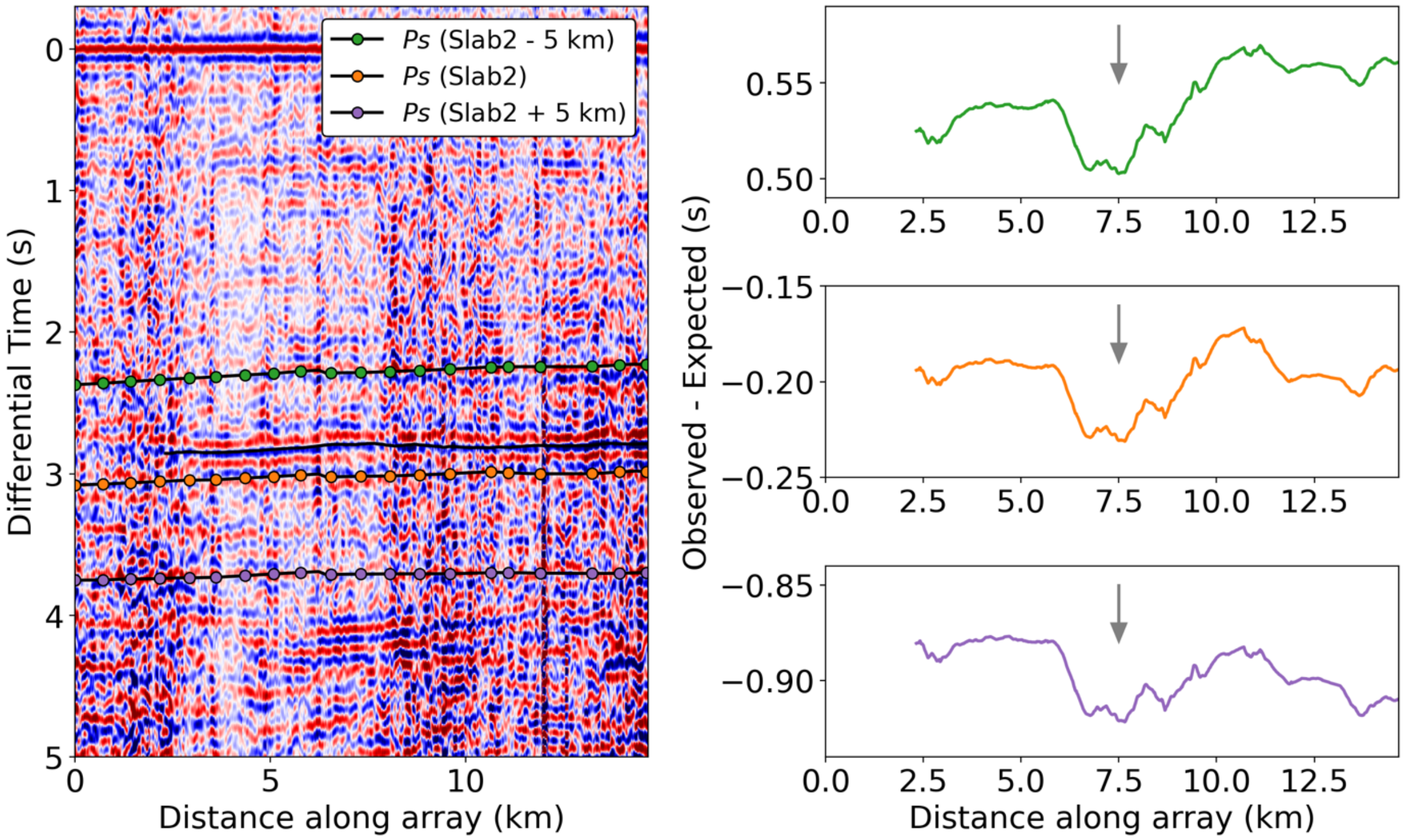

Figure S5. Representation of the difference between the correlogram peak picks and the expected arrival times for Ps conversions from Slab2, Slab2 made shallower by 5 km, and Slab2 made deeper by 5 km, computed using the 3D raytracing technique described in the paper. Left: the correlogram for nc75070761 with the expected arrival times of Ps for different slab depths plotted. The black line indicates the picks of the correlogram peaks. Right: representation of the differences between the Ps picks and the expected arrival times for Ps for the different slab depths. Colors correspond to the model changes indicated in the correlogram legend. The vertical scales of these plots are the same but shifted. Note that the narrow, negative dip in differences between observed and expected indicated by the gray arrow is present regardless of the absolute Slab2 depth being used to compute the expected arrival times.

3. DAS-specific variability in amplitude and polarity underestimated
The authors interpret variations in converted phase amplitude and polarity as evidence for fluid distribution in the slab. From my understanding, when working with DAS, especially telecom cables, lateral variations in amplitude and coherence are complex to model, either source related, but also installation dependent (e.g., near surface effects). Thus, the amplitude and polarity based interpretations (Figure 4) remain vulnerable to the azimuthal sensitivity issue (did authors take into consideration this?). An analysis of fiber azimuth variations and their expected effect on the DAS correlogram polarity for each event might be useful.

Yes, we took this into account. There are a couple of things to consider here. 1. We interpret polarities from an autocorrelation. This means that polarities of secondary phases are given

relative to the polarity of the first arrival recorded by the same station. This corrects for any polarity changes due to azimuthal variability in the orientation of the DAS array at the surface, because the azimuth of measured strain will be the same for the first arrival and any secondary arrival measured by the same channel. 2. The response of a strainmeter to an incoming plane wave is azimuthally dependent. However, because the onsets of the direct phases are actually dominated by locally scattered surface waves (e.g., Figure 1), and these scattered surface waves propagate concentrically from a secondary source near the array, it is expected that a strong component of particle motion will propagate parallel or subparallel to the array. Thus, if there is energy propagating to the surface, these secondary sources will be activated and will be observed at the surface, even if the incident energy from the direct wave is broadside. This was recently demonstrated in another study using data from this array, which showed that the S/P ratios of DAS data were comparable to those of collocated inertial seismometers. This suggests that the scattered surface waves are the dominant signal near the onset of incident energy in DAS and minimize the complications associated with the different directional sensitivity of uniaxial strain, at least for the application presented in our study. This study is now cited in the text.

Statement in the text on the polarization of incoming energy:

"Moreover, even if the polarization of the incoming converted wave is oriented at an unfavorable angle relative to the cable azimuth, the locally generated scattered energy will likely include some energy that propagates along the cable azimuth and be well recorded.[59]"

Where: Tracked changes version L484-486

Skoumal, R. J., Atterholt, J., Barbour, A. J. & Hardebeck, J. L. S / P Amplitude Ratios with Distributed Acoustic Sensing and Application to Earthquake Focal Mechanisms. Geophysical Research Letters 52, e2025GL116373 (2025).

The presence of these scattered waves is one of the challenges this paper seeks to overcome. By cross correlating the wavefields, we obtain a simplified wavefield with a pickable peak capturing the differential time between the direct waves converted waves (by correlating identical scattered wavefields generated by these waves).

4. Manuscript structure

The Results section is largely interpretation of the results, thus subjective. In my understanding, resuylts must be objective, then the discussion should built on the results the subjective descriptions, thus supporting conclusions. That's what readers expect. The Discussion section is too long, making the paper a bit hard to follow, especially at a first read. Maybe using more subchapters might help.

In practice, results should present observations and measurements (correlograms, picks, and inverted slab depths), while the Discussion should contain physical interpretation, comparison with other studies, limitations, and implications.

Thank you for this comment. We feel that this paper is best structured by including some discussion of the results where the results are introduced. To ameliorate this confusion, we have removed the “Results” title and preserved the titles within the results section. We agree the discussion section was too long, and we have broken down what was formerly the discussion section into two parts: “Challenges and Uncertainties” and “Implications for Future Studies.”

Minor comments
Figure 5 panel c: The converted phase lines are difficult to see against the correlogram background. Please increase the line weight or use a higher-contrast color scheme for these lines

Thank you for identifying this, we have corrected the figure accordingly.

DAS installation: Data should include a more detailed description of the fiber geometry.

The details of the installation and its metadata are discussed in the USGS data release (ref 16) and a previous paper (ref 17). We have expanded on the introduction of the array to describe the cities it connects and its azimuthal diversity:

“In May of 2022, a DAS array was deployed along approximately 15 km of telecommunications fiber north of Cape Mendocino and above the Gorda Slab (Figure 1)[16,17]. The array traverses an azimuthally diverse path between Arcata and Eureka, California.”

Where: Tracked changes document L85-87.

line 195: ..

Corrected. Thank you.

I thank the authors for their responses to my previous comments, which have clearly improved the manuscript. Regarding point 3, however, after reading again both the response and the revised manuscript, I remain somewhat hesitant.

Thank you for carefully reading our manuscript and providing your thoughts. We have endeavored to address each of your comments.

Main issue: kilometer-scale interface roughness and its implications in the overall picture of the manuscript
I still cannot accept the manuscript in this version because the implications regarding kilometer-scale interface roughness are not well supported by the data. I am satisfied with the observations of converted phases on autocorrelograms, which clearly demonstrate the advantage of having such a high-resolution tool as DAS. However, I am not convinced by the interpretation based on only one, or at most two, events of the relative changes in depth of the slab along the profile (the slab bump). The authors should exclude the possible effect of near-surface variations in the velocity structure, which could potentially explain differences of 0.01–0.05 seconds in the observed-minus-expected times.

We appreciate this concern, and we have done extra work to address it. Firstly, we understand your concerns about some of the specific language used that implies certainty about this interpretation (listed below) and have amended each of those statements.

We can also add a couple of things here that might alleviate your concerns further. We note that in a separate study of ours, which is now in press in GRL, we characterized the near surface structure beneath the array (top 1 km). We can compare this model to the changes in slab depth as measured across the array (Figure R1). This figure shows that there is not a correlation between the slab-informing measurements and the near-surface velocity structural heterogeneity. This figure also shows that bump is recorded at very different segments of the array between events. If the bump were resulting from shallow structure, we would expect the same segment of the array to be informing the bump between events (because the raypaths between the events should be similar at shallow depths).

We now cite this study, which includes the velocity model and discuss its implications for shallow structural effects:

“This is also indirectly supported by the observation that shear velocity heterogeneity in the shallowest 1 km beneath this array is not correlated with the perturbations in the Ps differential times that inform the bump.”

Where: L380-382

REDACTED

Figure R1. Comparison between a near surface velocity model (Vs) computed via ambient noise cross correlation in Atterholt et al. (in press) and the computed changes to slab depth from the events in Figure S2 represented where the in-situ P-to-Ps differential times are made along the array.

We additionally plotted the power spectral density from shallow events (as requested) below. This PSD plot does not show lateral heterogeneity that is correlated with the plots shown in Figure R1.

We would also point to several items in the original draft that address this concern directly. In the original manuscript we performed synthetic tests to evaluate the sensitivity of the results to near surface structural heterogeneity necessary to produce a bump on the scale observed in this study (Figure S8):

“The roughness measured on the megathrust fault in Figure 5 is of lower amplitude than the absolute change in slab depth (~300 m). To produce this peak, there would need to be a sharp-gradient inaccuracy in the relative Vp/Vs ratio along the array. This inaccuracy would likely need to be kilometers deep and narrow (Figure S8).”

Where: L374-377

We then go on to say that this is possible, and our interpretation is only preferred because of the multiple observations of the bump across events.

"This is conceivable but given that our observations can be verified across events and at different channels (Figure S2), we prefer the interpretation that the most prominent features are indeed slab topography."

Where L377-380

Given the array geometry and uncertainty in the velocity model, we cannot easily decouple the effects of velocity from the effects of slab geometry. We noted this in the original manuscript:

"These tradeoffs show that short-wavelength slab topography, which may be important for the next generation of slab models, is best resolved with accurate and precise 3D velocity models with sensitivity to the full crustal section."

Where: L385-388

Citation:

Atterholt, J., Barbour, A.J., McGuire, J.J., Moschetti, M.P. Investigating Meter-Scale Wavefield Complexity at the Basin Edge with a Fiber-Optic Array. *Geophys. Res. Lett.* (in press).

Additionally, it's unclear to me how these phases were picked.... by hand?

Regarding how the picks are made, this is described in the main text of the manuscript. The picks are made by choosing a local maximum within a time window around the correlation peak. They are thus made objectively.

"Once classified, we make picks on the phase differential times by finding a local maximum or minimum within a time window of the expected arrival time of the phase of interest. Whether the maximum or minimum is used is decided by visual inspection for an axis of symmetry in the stacked autocorrelogram peak (or trough)."

Where: L137-141

I noted that the signal anomalies being interpreted fall within the tens-of-milliseconds range (Figure S5), how do they relate to picking uncertainty?

This is an interesting question. Because many picks are made within the spatial window encompassed by a wavelength, the error is greatly reduced through fitting the curve with a

Gaussian process. As stated in the main text, the uncertainty is conservatively estimated in way that is related to the period of the correlation peak:

"This approach prioritizes the high temporal frequency phases that were confidently identified and classified using the spatial density of DAS to infer slab properties with high precision (~half the period of the correlation peak) and a measure of uncertainty."

Where: L222-225

We also state in the text that this translates to a standard deviation of the picks of ¼ the period of the correlation peak for the highest quality correlograms:

"The standard deviation on the picks, which constrains the Gaussian process error, is assumed to be 1/4 the period of the correlation peak (assuming a frequency of 6 Hz) for the best constrained picks (nc75070761) and 1/2 the period of the correlation peak for the more poorly constrained picks (nc73947920)."

Where: L570-573

This translates to an uncertainty of 0.05 s (or ~5 samples) for the highest quality picks. This, in turn, translates into an approximate depth uncertainty of 0.25 km per pick. The reason we can constrain the bump is because we have many picks per wavelength which, together, tamp down the overall uncertainty on the slab curve. The Gaussian process pools information across nearby picks under a smoothness prior whose length scale is set to the approximate wavelength of the phase, matching the spatial resolution limit of the data. Within one length scale, the posterior depth is informed by many picks, which reduces the posterior variance well below the per-pick uncertainty. This is how we get the error bars on the curve in Figure 5 and elsewhere.

We amended the text to include this more explicit description of how the Gaussian process is getting the uncertainty:

"That is, we impose a spatial correlation length equal to the expected wavelength, consistent with the intrinsic resolution of the converted phase. Prescribing this length scale allows the Gaussian process to pool information across nearby picks, reducing the posterior uncertainty below the per-pick uncertainty."

Where: L566-570

Furthermore, the potential effect of coupling variability along the telecom cable has not been discussed. What do authors think about the possibility that spatially-irregular cable coupling can generate anomalies in the correlograms that mimic deeper structural variations?

Thank you for this question on coupling. The primary effect of poor coupling is on amplitude (that is, diminished amplitudes). This does not affect our arguments that are dependent on phase differences. In general, empirically, phase has been shown to be captured accurately and precisely by DAS (Lindsey et al., 2020). There are theoretical studies that have suggested that very strong site variability can affect phase arrivals. We can discount this for a couple of reasons. 1. The correlation is capturing the difference in phase between two arrivals recorded at a single site, largely cancelling out any potential phase delay due to site effects. 2. We know that this particular array is not subject to very strong coupling variability. In the GRL paper (in press) cited above, we also calculate the array response of the fiber using a collocated nodal array. That is, we convert the strain as measured by the DAS to ground motion and compare those ground motions to ground motions measured by well-coupled nodes. The result is this curve, which shows that across the entire array there is not really an extreme case of poor coupling:

REDACTED

Figure R2. Comparison of DAS measurements converted to ground motion using the curvelet conversion methodology outlined in Atterholt et al. (2021) and Yang et al. (2021) compared to ground motion measurements from a collocated nodal array. This plot is shown in Atterholt et al. (in press).

Citations:

Atterholt, J., Zhan, Z., Shen, Z., Li, Z. A Unified Wavefield Partitioning Approach for Distributed Acoustic Sensing. Geophys. Journal Int. 228. 1410-1418. (2021).

Yang, Y., Atterholt, J. W., Shen, Z., Muir, J. B., Williams, E. F., & Zhan, Z. Sub-Kilometer Correlation Between Near-Surface Structure and Ground Motion Measured with Distributed Acoustic Sensing. Geophysical Research Letters, 49. e2021GL096503 (2022).

Lindsey, N. J., Rademacher, H., & Ajo-Franklin, J. B. On the Broadband Instrument Response of Fiber-Optic DAS Arrays. Journal of Geophysical Research: Solid Earth, 125. (2020).

Atterholt, J., Barbour, A.J., McGuire, J.J., Moschetti, M.P. Investigating Meter-Scale Wavefield Complexity at the Basin Edge with a Fiber-Optic Array. Geophys. Res. Lett. (in press).

As you might understand, my concern is that I would like to exclude the near surface effects in potentially providing similar time differences to deeper (at the slab interface) lateral topographic anomalies. Why? Because being capable of image such slab topography at such fine scale has large potential implications for subduction zone and earthquake rupture models. Either authors relax their claims on this or support it with more data excluding near surface effects.

We agree this is important. We would stress that this paper is not making an argument that this allows for the resolution of fine-scale slab topography absent a very good velocity model. Rather, we argue that by making high frequency measurements of phases from the slab measured at high spatial frequencies at the surface possible, that it is possible to take advantage of good velocity models to get fine-scale slab structure at depth, whereas with lower frequency, lower density measurements it is not. We took note of the specific concerns about the language used in reference to our resolution of the bump below and amended those phrases accordingly.

A possible suggestion to the authors to exclude influence of the near surface: Do you have DAS recordings of shallower events, not intraslab? Do you see lateral variations in the frequency content of such signals (e.g., power spectral densities) or systematic delays in arrival times of direct phases in such events? If you see similar trends (not absolute values of course, but similar relative shapes) to what you observe on predicted-observed times this might be an indication of the influence of near-surface. I would say that also with different shapes but similar relative changes in the traveltimes it would be alarming for the claims on the slab origin of lateral variations in delay times compared to the expected times. If instead the shapes are different and the influence of the near surface is smaller, these results might support the claims on the deeper origin of the anomalies.

Thank you for these suggestions. We hope that providing the shallow velocity model and showing that it does not correlate with the observations informing the slab bump in Figure R1 alleviates the reviewer's concerns about the influence of near-surface structure. For completeness, in response to this request, we have computed the PSD for each of the channels along the array. While somewhat noisy, we note that the PSD across the array does not produce patterns that correspond to the bumps observed by the array (compare with Figure R1).

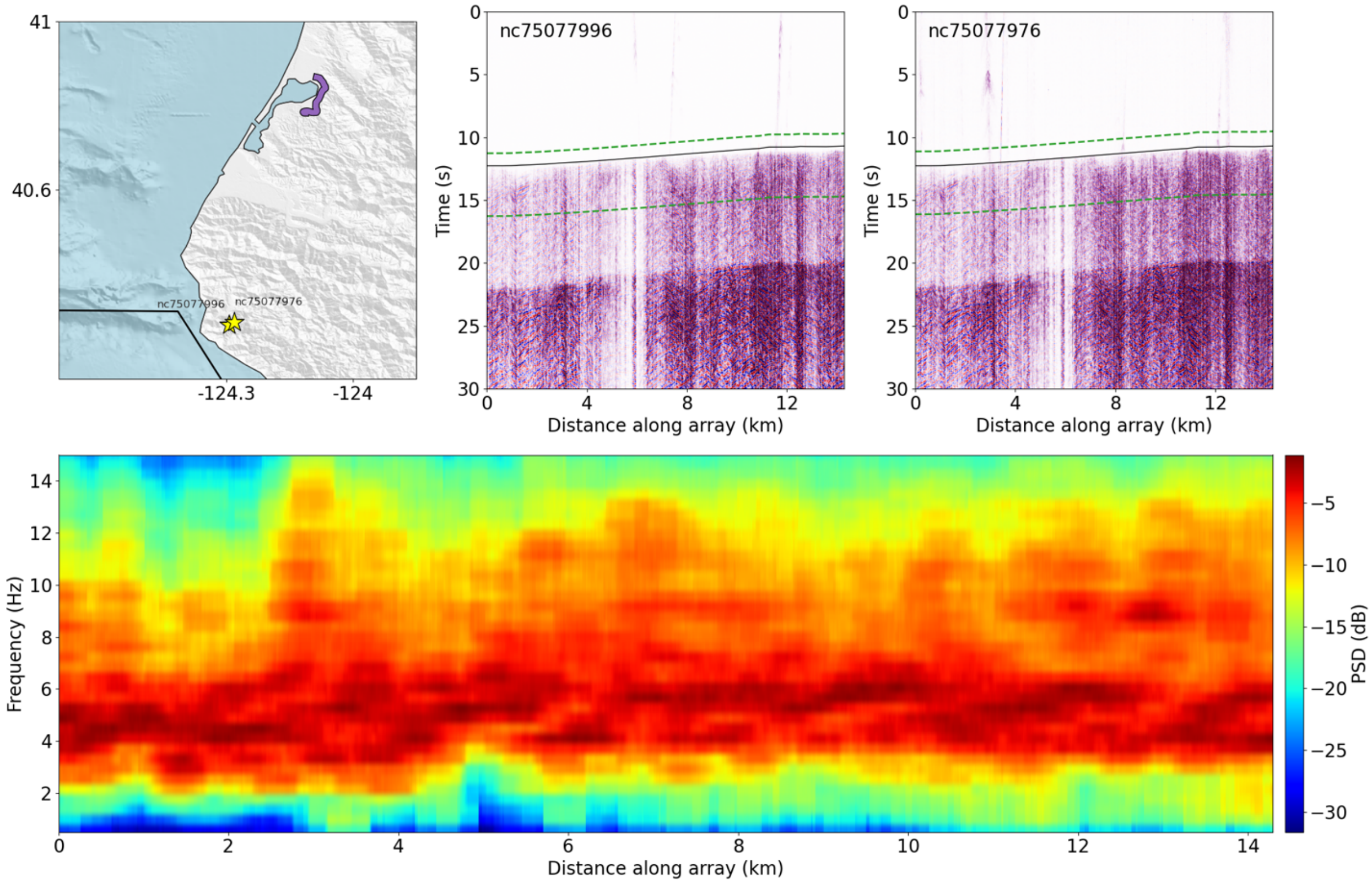

Figure R3. Representation of the normalized stacked power spectral density across two shallow events (~9 km depth) with epicenters in the vicinity of deeper, intraslab events used to image the slab in Figures 5 and S2. Note there are not clear PSD anomalies along the sections of the array that inform the bump on the slab (6-10 km and 8-13 km).

I would also recommend providing zoomed time windows around the picks in Figure 3, which is a key for better understanding the spatial coherence on the converted phases and the uncertainties in the picks.

Thank you for this recommendation. We considered adding zoomed panels but felt they would duplicate information already visible in Figure 3 (scatter and correlation peak quality), so we would prefer to leave that representation as-is.

Some examples from the text:

Regarding lines 271–276, the authors argue that the similar bump observed in two profiles recorded on different fiber segments excludes a near-surface origin. This reasoning needs to be explained more clearly: why is it self-evident that two events recorded on partially overlapping fiber segments rule out a near-surface structural effect?

This supports our argument because shallow features influence the wavefield at a position that is close to the array. So, even for different events at different locations, the raypaths near the array from both events will be very similar and therefore a shallow feature should be observed by both events at approximately the same channels. Because the bumps between those two events are observed at distinct channel windows, this suggests the feature causing these perturbations is some distance from the array. We have added clarification in the text:

"For one, this suggests that this bump is not due to shallow structural effects because it is recorded by two events on different segments of the fiber (6-10 km along fiber for nc75070761 and 8-14 km along fiber for nc73947920). This is because raypaths from different events converge as they approach the array, so shallow structure beneath the fiber is sampled in common by both events and would impose a similar signature in each."

Where: L275-278

More broadly, the manuscript is inconsistent in how it presents the robustness of the slab topography interpretation. In some sections the roughness finding is addressed as uncertain (e.g., lines 392-), while in others it reads as a well-established result, for example:

We appreciate your concerns, and we address each of them below.

Lines 291–293 state: "This inversion makes clear that fiber arrays can be used to image sub-kilometer megathrust topography at deeper depths than are accessible with standard techniques."

We have modified this with the caveat that there needs to be a sufficiently accurate velocity model to get this topography:

"This inversion makes clear that, with a sufficiently accurate velocity model, fiber arrays can be used to image sub-kilometer megathrust topography at deeper depths than are accessible with standard techniques."

And also in the abstract (lines 19–21): "…which reveals kilometer-scale interface roughness."

In this case, we have changed the problem word “reveals” to “suggests”

The terms “makes clear” and “reveals” are typically assured facts, not uncertain.

Indeed, we have adjusted this wording accordingly.

REVIEWERS' COMMENTS

Reviewer #4 (Remarks to the Author):

Review #2 Atterholt Nature comm. 2026

Atterholt and coauthors provided a very detailed response to my concerns. I am satisfied with the answers. I suggest reporting these additional pieces of evidence (e.g., the figures with the near surface velocity), if possible, in the supplementary material of the final version of the manuscript. This imo would strengthen the support for the results discussed in the manuscript.

I also appreciate the more cautious tone of the revised manuscript, which now reflects that the results suggest, rather than clearly demonstrate, that converted phases, using the proposed methodology and within the analyzed context, can image heterogeneities in the slab topography. This is important, as in my opinion, there is space for other studies in other contexts, and with a longer cable, to further confirm this.

Thank you for taking the time to review our manuscript and we appreciate the additional comments. We have considered each comment carefully and modified the manuscript accordingly. Regarding the near surface velocity figure, we prefer to just reference the near surface model so that the readers can evaluate the near surface structure in the same publication where the structural inversion is described.

Further minor revisions (no need to send the manuscript back to me but I'd be happy if authors try to implement them):

Line 271:

"In particular, we resolve a bump that is 300 m high and 3 km wide." →

"In particular, the results point to the possible presence of a bump approximately 300 m high and 3 km wide."

These values carry uncertainties, so they should not be stated without clarifying that they are estimates.

Thank you for this suggestion, we have made this change.

Figure 1a-b

I would suggest highlighting the conversion points and their epicentral location to better understand the square points in a) and the corresponding model in b). In practice, make crystal clear that those square points are indeed the “epicenters” of the conversion point at depth.

Thank you for this comment. We now reference 1b in the text describing location of the conversion points in 1a.

“Also plotted as square points are changes to Slab2 resolved in this study (Figure 3b) at the average location of the conversion points (which is distinct from the event epicenter as illustrated in 1b) used to make the estimate (see Methods).”

Figure 1c-d

Please make sure that from the figure itself it is clear that those lines are synthetic traveltimes. I know this is already stated in the caption, but with the new AI pickers for DAS data sometimes it’s not immediately clear the difference between picked phases and synthetic traveltimes.

Thank you for this suggestion, but we feel it is sufficient to state this in the figure caption.

Figure 4b

The color for the caption Vp and Vs are almost invisible. It might help using bold characters to highlight the color.

Thank you for this suggestion, we have changed the Vp and Vs text to make them bold in 4b.
